# Breast cancer burden risk attribution and productivity loss in Chinese women aged 25 to 49

**DOI:** 10.1016/j.isci.2026.117891

**Published:** 2026-10-14

**Authors:** Yang Bai, Jiaqi Zhang, Tian Tian, Ke Wang, Jian Zhang

**Affiliations:** 1 Department of Breast Surgery, The First Affiliated Hospital of Xi’an Jiaotong University, Xi’an, Shaanxi, China

**Keywords:** China, early-onset breast cancer, disease burden, population attributable fraction, exploratory feature ranking, mortality-related productivity loss, Bayesian projection

## Abstract

Breast cancer among Chinese women aged 25–49 years has substantial clinical and societal consequences. We integrated Global Burden of Disease (GBD) 2023 estimates for 1990–2023 with Global Cancer Observatory (GLOBOCAN) 2022, United Nations population projections, International Labour Organization labour-force participation, and World Bank indicators. Incident cases rose from 50,064 to 87,221, while the weighted death rate declined from 9.25 to 4.72 per 100,000. Demographic changes explained 63.0% of additional cases. In exploratory extreme gradient boosting (XGBoost) analyses interpreted with SHapley Additive exPlanations (SHAP), the fasting-plasma-glucose-related population attributable fraction (PAF) ranked first in several formulations but weakened after removing age and temporal structure; the selected high-body-mass-index (BMI) PAF series was constant at zero and unrankable. Potential mortality-related productivity loss was US$6.161 billion in 2023. Status-quo projections suggested increasing incidence through 2050 with very wide uncertainty, supporting surveillance and individual-level validation rather than immediate screening policy changes.

## Highlights

• Breast cancer cases rose among Chinese women aged 25–49 years, 1990–2023
• Demographic changes explained 63.0% of the increase in incident cases
• Fasting plasma glucose ranking partly depended on age and temporal structure
• Potential mortality-related productivity loss was US$6.161 billion in 2023

## Graphical abstract

**Figure:**
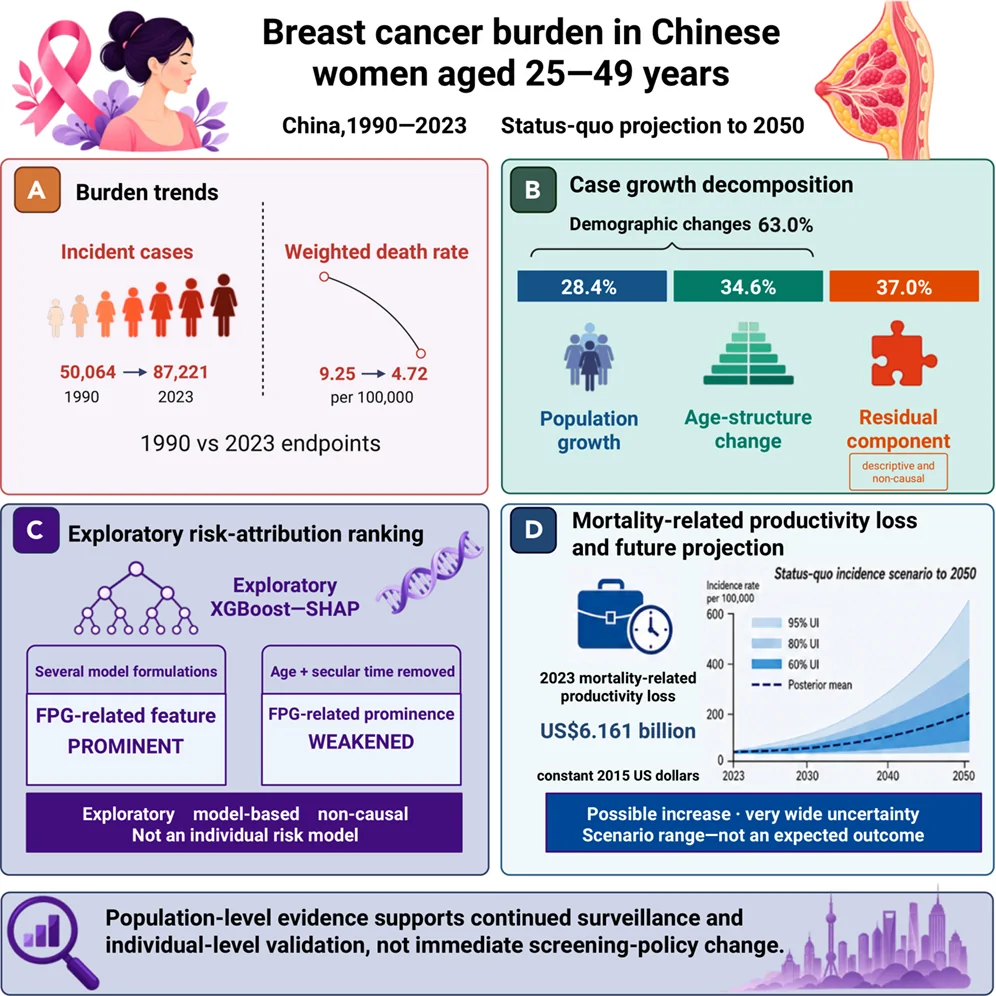

## Teaser

Health sciences; Medicine; Medical specialty; Oncology; Public health

## Introduction

Breast cancer is the most frequently diagnosed female malignancy and a leading cause of cancer death worldwide.^1^ Although the clinical and screening literature has long focused on postmenopausal disease, breast cancer diagnosed before age 50 is increasingly recognized for its distinct clinical and societal consequences.^2^ This burden is especially consequential in China, where a large proportion of affected women are of economically active age. Consequently, deaths occurring between 25 and 49 years contribute substantial years of life lost and may also have productivity consequences during economically active ages.^3^^,^^4^

Despite growing awareness, several critical questions remain insufficiently addressed. First, increases in absolute case numbers may reflect population growth, changes in age structure, and residual changes in age-specific incidence. Distinguishing these components is important because similar increases in case numbers may imply different public-health priorities.^5^ Second, elevated fasting plasma glucose and high body mass index are biologically plausible contributors to breast cancer, but their population-level patterns among younger and middle-aged women and their relative contributions within a selected set of GBD-derived population attributable fractions remain uncertain.^6^^,^^7^^,^^8^^,^^9^^,^^10^^,^^11^ Any model-based ranking of these features is exploratory and cannot establish individual-level or causal effects. Third, premature mortality in this age group has productivity consequences, yet few studies have integrated age-specific years of life lost with contemporary female labor-force participation and economic indicators.^3^^,^^4^ Finally, long-term projections require transparent assumptions and external comparison of the incidence baseline; agreement in age patterns or baseline levels should not be interpreted as formal validation of future trajectories.

We therefore analyzed breast cancer among Chinese women aged 25–49 years from 1990 to 2023 to distinguish demographic contributions from the residual epidemiological component of case growth, describe cohort-specific incidence patterns, exploratorily rank selected GBD-derived risk-specific population attributable fractions in relation to model-predicted disability-adjusted life years, and estimate mortality-related productivity loss. We also projected incidence through 2050 under a status-quo Bayesian age-period-cohort scenario and compared the 2022 incidence baseline with rates derived from GLOBOCAN case estimates. This integrated population-level analysis was designed to characterize burden, identify which exploratory signals were stable across model formulations, and quantify scenario-dependent future patterns; it was not designed to infer individual causal effects or to evaluate specific screening policies.

## Results

### Baseline burden and temporal trends

Baseline burden estimates revealed substantial differences in both absolute numbers and rate-based trends across SDI strata, globally, and in China. Globally, incident cases among women aged 25–49 years increased from 278,566 (95% UI, 244,818-318,547) in 1990 to 672,228 (95% UI, 560,147–806,448) in 2023, while the weighted ASIR increased from 36.39 (95% UI, 32.02–41.56) to 48.36 (95% UI, 40.28–58.02) per 100,000. In China, incident cases increased from 50,064 (95% UI, 37,131-64,200) to 87,221 (95% UI, 61,492-122,482), and the weighted ASIR increased from 28.06 (95% UI, 20.84–35.86) to 32.87 (95% UI, 23.19–46.18) per 100,000. However, the ASIR EAPC for 1990–2023 was −0.16% (95% CI, −0.36%–0.04%), indicating that the higher 2023 endpoint occurred within a non-linear long-term trajectory rather than a sustained monotonic increase. Over the same period, deaths decreased from 16,414 (95% UI, 12,412-20,953) to 12,611 (95% UI, 9,232-16,869), and the weighted ASDR decreased from 9.25 (95% UI, 7.00–11.78) to 4.72 (95% UI, 3.45–6.33) per 100,000 (EAPC, −2.83%; 95% CI, −3.09% to −2.57%) (Figure 1 and Table 1).

**Figure 1:**
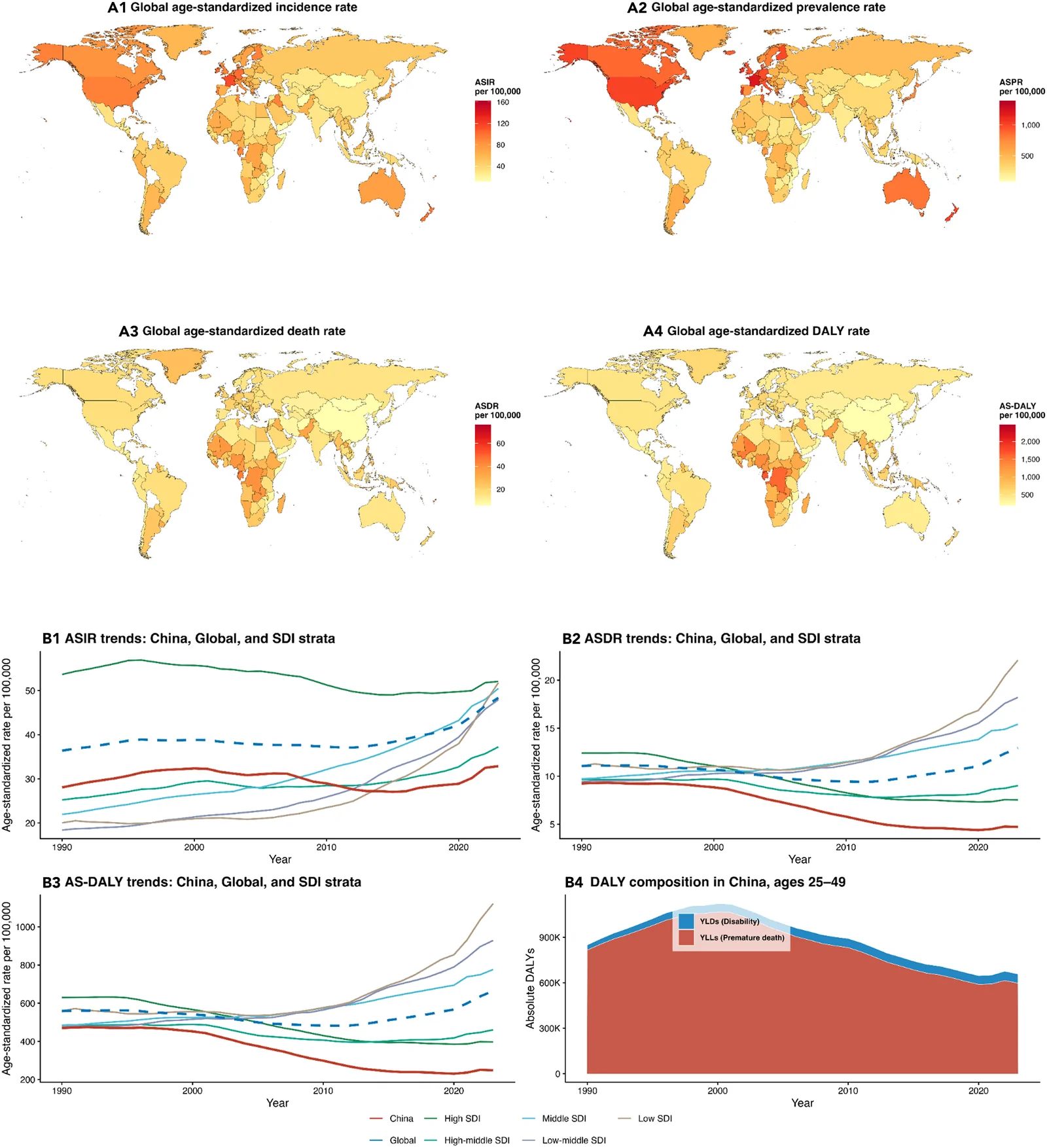
Breast cancer burden and temporal trends, 1990–2023 The upper images show the global distribution of all-age age-standardized incidence (A1), prevalence (A2), death (A3), and DALY (A4) rates in 2023. The lower images show weighted age-standardized incidence (B1), death (B2), and DALY (B3) rates among women aged 25–49 years across China, the global population, and the five SDI reference strata. B4 shows the YLL and YLD composition of DALYs in China among women aged 25–49 years. ASIR, age-standardized incidence rate; ASPR, age-standardized prevalence rate; ASDR, age-standardized death rate; AS-DALY, age-standardized disability-adjusted life-year rate; DALY, disability-adjusted life year; YLL, year of life lost; YLD, year lived with disability; SDI, Socio-demographic Index.

**Table 1:** Breast cancer burden and temporal trends among women aged 25–49 years, 1990–2023

A. Incidence burden and temporal trends					
Location	Incident cases, 1990 n (95% UI)	ASIR, 1990 per 100,000 (95% UI)	Incident cases, 2023 n (95% UI)	ASIR, 2023 per 100,000 (95% UI)	ASIR EAPC, % (95% CI)
China	50 064 (37 131 to 64 200)	28.06 (20.84–35.86)	87 221 (61 492 to 122 482)	32.87 (23.19–46.18)	−0.16 (−0.36 to 0.04)
Global	278 566 (244 818 to 318 547)	36.39 (32.02–41.56)	672 228 (560 147 to 806 448)	48.36 (40.28–58.02)	0.40 (0.22–0.57)
High SDI	184 164 (164 593 to 204 903)	53.65 (48.00–59.67)	250 244 (215 636 to 287 282)	52.08 (44.85–59.81)	−0.43 (−0.53 to −0.33)
High-middle SDI	40 701 (32 426 to 48 502)	25.23 (20.17–29.98)	114 514 (93 655 to 139 080)	37.25 (30.44–45.24)	0.75 (0.59–0.91)
Middle SDI	17 215 (13 896 to 21 317)	21.96 (17.71–27.19)	87 495 (68 965 to 110 045)	50.49 (39.79–63.51)	2.35 (2.20–2.51)
Low-middle SDI	17 211 (13 217 to 22 478)	18.38 (14.13–23.98)	94 277 (72 279 to 121 610)	47.93 (36.78–61.76)	2.78 (2.51–3.05)
Low SDI	18 960 (13 705 to 25 764)	20.03 (14.49–27.13)	125 121 (91 810 to 164 463)	51.79 (38.11–68.04)	2.46 (2.00–2.92)

B. Mortality burden and temporal trends					
Location	Deaths, 1990 n (95% UI)	ASDR, 1990 per 100,000 (95% UI)	Deaths, 2023 n (95% UI)	ASDR, 2023 per 100,000 (95% UI)	ASDR EAPC, % (95% CI)
China	16 414 (12 412 to 20 953)	9.25 (7.00–11.78)	12 611 (9 232 to 16 869)	4.72 (3.45–6.33)	−2.83 (−3.09 to −2.57)
Global	84 800 (75 013 to 94 443)	11.07 (9.80–12.30)	179 756 (147 225 to 215 234)	12.92 (10.58–15.48)	−0.03 (−0.30 to 0.24)
High SDI	42 525 (40 085 to 45 014)	12.40 (11.70–13.12)	36 344 (33 514 to 39 444)	7.53 (6.94–8.18)	−1.99 (−2.14 to −1.83)
High-middle SDI	15 466 (12 490 to 18 015)	9.65 (7.82–11.23)	27 810 (23 091 to 33 040)	9.02 (7.49–10.73)	−0.67 (−0.85 to −0.48)
Middle SDI	7 565 (6 143 to 9 324)	9.73 (7.89–12.00)	26 739 (21 078 to 33 211)	15.41 (12.15–19.14)	1.25 (1.11–1.39)
Low-middle SDI	8 760 (6 739 to 11 281)	9.42 (7.25–12.12)	35 719 (27 300 to 45 513)	18.21 (13.93–23.16)	1.83 (1.58–2.08)
Low SDI	10 386 (7 606 to 13 945)	11.07 (8.11–14.82)	52 991 (38 156 to 68 590)	22.09 (15.93–28.57)	1.67 (1.25–2.09)

Note: Values are absolute numbers and weighted age-standardized rates for ages 25–49 years per 100,000, with 95% uncertainty intervals. In both A and B, EAPC denotes estimated annual percentage change and is presented with 95% confidence intervals. ASIR, age-standardized incidence rate; ASDR, age-standardized death rate; EAPC, estimated annual percentage change; UI, uncertainty interval.

In 2023, the weighted ASIR remained highest in high-SDI regions at 52.08 per 100,000 and ranged from 37.25 to 51.79 per 100,000 across the other four SDI strata. Although the high-SDI ASIR was slightly lower than in 1990, middle-, low-middle-, and low-SDI regions showed positive EAPCs. In China, YLLs decreased from 816,139 (95% UI, 616,204-1,043,382) in 1990 to 597,996 (95% UI, 436,745–801,542) in 2023, whereas YLDs increased from 33,626 (95% UI, 21,684-50,127) to 60,262 (95% UI, 38,889-93,464). Total DALYs in 2023 were 658,257 (95% UI, 487,978-879,835), corresponding to a weighted DALY rate of 248.61 (95% UI, 184.07–332.76) per 100,000. YLLs accounted for approximately 90.8% of total DALYs (Figure 1 and Table S14).

### Decomposition analysis and cohort-specific patterns

To examine the increase in case numbers, the change in incident cases in China between 1990 and 2023 was evaluated using the sequential decomposition pathway shown in Table 2. Starting from 50,064 observed cases in 1990, replacing total population size increased the counterfactual count to 60,632, and subsequently replacing age structure increased it to 73,489. The remaining difference to the observed 87,221 cases in 2023 was assigned to the residual epidemiological component. The corresponding incremental contributions were 10,568 cases (28.4%) for population growth, 12,856 (34.6%) for age-structure change, and 13,732 (37.0%) for the residual component. Thus, demographic changes together accounted for 63.0% of the increase, while the remaining 37.0% was not explained by population size or age structure (Figures 2A and 2B and Tables 2 and S2).

**Table 2:** Sequential decomposition pathway of the increase in incident breast cancer cases among Chinese women aged 25–49 years, 1990–2023

Sequential stage	Component represented	Estimated incident cases, n	Incremental contribution, cases	Share of total increase, %
Observed 1990 baseline	–	50 064	–	–
After replacing total population size	Population growth	60 632	10 568	28.4
After additionally replacing age structure	Age-structure change	73 489	12 856	34.6
Observed 2023 endpoint	Residual epidemiological component	87 221	13 732	37.0

Note: Incident cases were sequentially decomposed using a prespecified replacement approach adapted from the framework described by Das Gupta.^12^^,^^13^ The observed 1990 case count served as the baseline. Total population size was replaced first, followed by age structure; the remaining difference between the demographic counterfactual and the observed 2023 case count was defined as the residual epidemiological component. The residual component is descriptive and should not be interpreted as a directly measured causal or metabolic effect. Stage-specific case counts, component contributions, and percentages were calculated from unrounded estimates and rounded separately for presentation; differences between adjacent displayed stage counts may therefore not exactly equal the displayed component contributions. Full results for China, the global population, and the five SDI strata are provided in Table S2.

**Figure 2:**
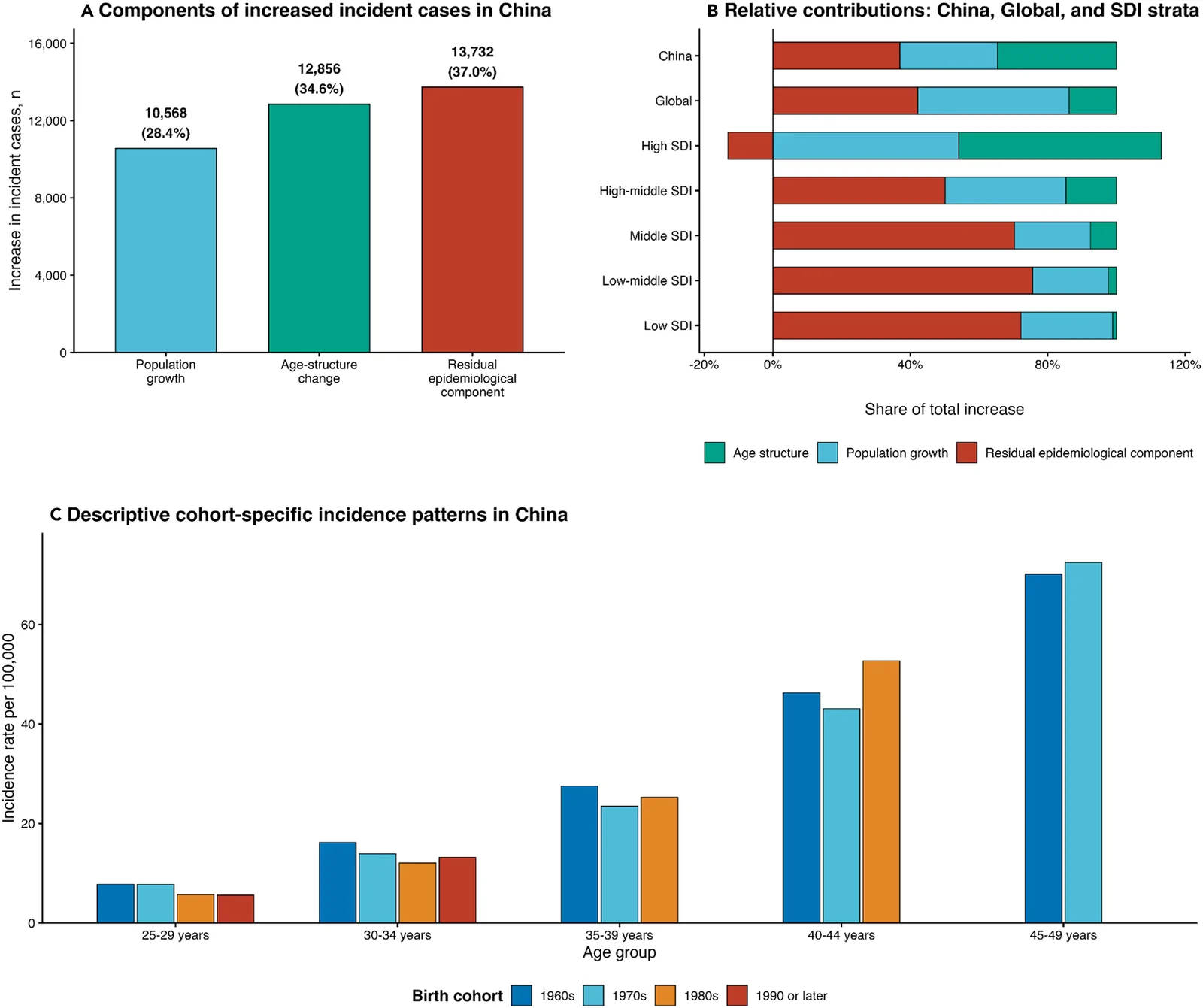
Sequential decomposition of increased incident cases and descriptive cohort-specific incidence patterns (A) Bar chart of the increase in incident cases in China between 1990 and 2023 attributable to population growth, age-structure change, and the residual epidemiological component. (B) Relative contributions of the three components in China, the global population, and the five SDI reference strata. (C) Grouped bar chart of descriptive cohort-specific incidence patterns in China. Later cohorts are shown only for age groups observable within the 1990–2023 study period; no interpolation or extrapolation was used for unobserved cohort-age combinations. SDI, Socio-demographic Index.

The cohort-specific descriptive analysis showed increasing incidence with attained age in each observable cohort. Women born in the 1960s and 1970s were observed through ages 45–49 years, whereas the 1980s and 1990-or-later cohorts were observed over progressively fewer older age groups. At comparable observed ages, the more recent cohorts did not reproduce the same degree of attenuation seen in some earlier cohort trajectories; however, direct comparisons at older ages were not possible because follow-up of the later cohorts was incomplete (Figure 2C).

### Risk attribution and exploratory feature ranking

Risk-attribution patterns differed by factor. The data audit identified no missing values in the DALY target or in any of the five prespecified China-specific PAF series across the 170 years-age observations. In 2023, the descriptive unweighted arithmetic mean of the five age-specific PAF estimates was highest for tobacco use (12.81%), followed by high FPG (4.02%), high alcohol use (1.45%), and low physical activity (1.26%). This mean was used only to summarize the five age groups and was not interpreted as an overall or population-weighted PAF for women aged 25–49 years. The FPG-related PAF increased with age, from 2.50% at ages 25–29 years to 5.56% at ages 45–49 years. Across 1990–2023, FPG-related PAFs declined through approximately 2010 and then increased modestly; tobacco-use PAFs declined gradually; and alcohol- and physical-activity-related PAFs showed smaller non-monotonic changes. The selected China-specific GBD breast-cancer DALY PAF series for high BMI was an observed zero in every year-age combination and therefore had zero variance across all 170 observations. This constant-zero pattern was a property of the selected risk-attribution series and was not an estimate of high-BMI prevalence among Chinese women (Figures 3A, 3C, 3D, and S3 and Tables S3, S7, S8, and S15).

**Figure 3:**
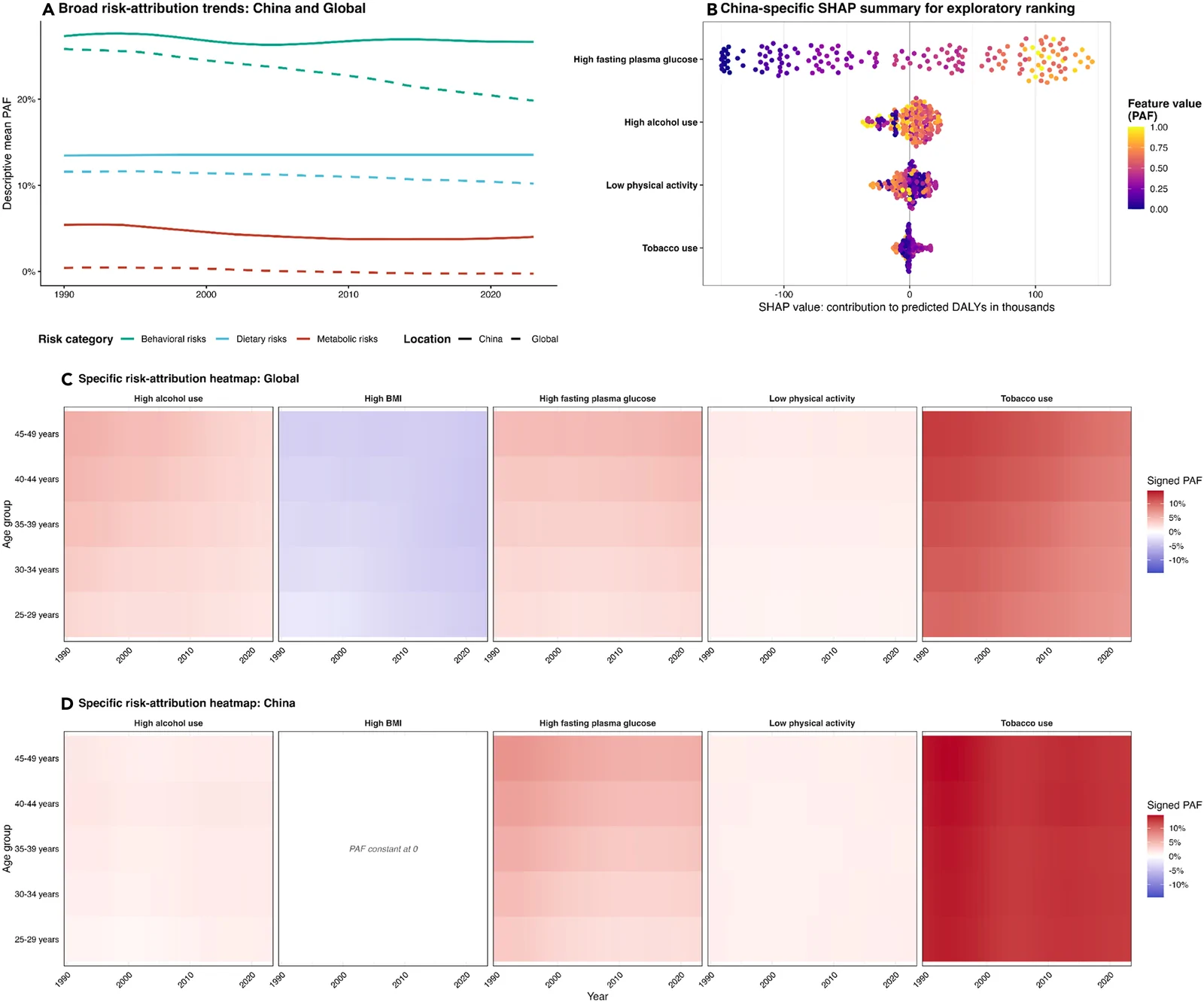
Risk-attribution patterns and exploratory feature ranking (A) Temporal trends in broad DALY PAF categories in China and globally; plotted values are unweighted arithmetic means across the five age groups and are used only as descriptive summaries, not as overall or population-weighted PAFs for women aged 25–49 years. (B) SHAP summary for the four nonconstant China-specific DALY PAF features. SHAP values represent contributions to model-predicted DALYs in thousands and do not indicate causal effects. (C and D) Signed age-specific DALY PAFs by calendar year globally and in China, respectively. The China-specific high-BMI PAF series was constant at zero and was retained descriptively but excluded from the XGBoost-SHAP ranking. DALY, disability-adjusted life year; PAF, population attributable fraction; BMI, body mass index; XGBoost, extreme gradient boosting; SHAP, SHapley Additive exPlanations.

The XGBoost-SHAP exploratory analysis produced a primary ranking among the four nonconstant features. High FPG ranked first with a mean absolute SHAP value of 85.3 thousand DALYs, followed by high alcohol use (12.1 thousand), low physical activity (8.8 thousand), and tobacco use (4.3 thousand). High BMI was retained in the descriptive PAF analyses but was excluded from the ranking because its constant-zero series could not contribute to a tree-based importance estimate. The difference between the tobacco-use PAF and its SHAP rank also showed that PAF magnitude and model-based contribution to variation in absolute DALYs were not equivalent quantities (Figure 3B and Tables S3 and S6).

Forward temporal validation showed that the leading feature differed in the earliest held-out period. High alcohol use accounted for 65.5% of normalized SHAP importance in Fold 1 (training, 1990–2005; testing, 2006–2011), whereas high FPG accounted for 23.5%. High FPG ranked first in Fold 2 (71.8%; testing, 2012–2017) and Fold 3 (83.7%; testing, 2018–2023). Held-out R-squared values were 0.477, 0.903, and 0.732, and Spearman correlations between observed and model-predicted DALYs were 0.772, 0.941, and 0.957, respectively. Across 200 grouped calendar-block bootstrap resamples, high FPG had a mean normalized importance of 77.1% and ranked first in 99.0% of resamples (Figure S1 and Table S9).

The FPG-related feature remained first in the full-sample age-specific DALY-rate, within-age-centered, and annual-first-difference formulations. In the corresponding grouped bootstrap analyses, it ranked first in 99.5%, 91.5%, and 78.0% of resamples, respectively. After age-group levels and smooth calendar-year trends were removed from both the target and features, high alcohol use ranked first in the full-sample model; high FPG had a mean normalized importance of 33.0%, a median rank of 2, and a top-rank frequency of 36.0%. The prominence of the FPG-related feature was therefore retained under several formulations but weakened after removal of age and secular temporal structure (Figure S2 and Table S10).

### MIR patterns and mortality-related productivity loss

MIRs differed across development levels. The weighted MIR among Chinese women aged 25–49 years decreased from 0.330 in 1990 to 0.144 in 2023. The crude deaths-to-incidence count ratio in 2023 was 0.145, closely approximating the rate-based MIR but representing a separate quantity. In 2023, China’s weighted MIR was lower than the global value of 0.267 and lower than the values observed in all five SDI reference strata (Figure 4A and Table S16).

**Figure 4:**
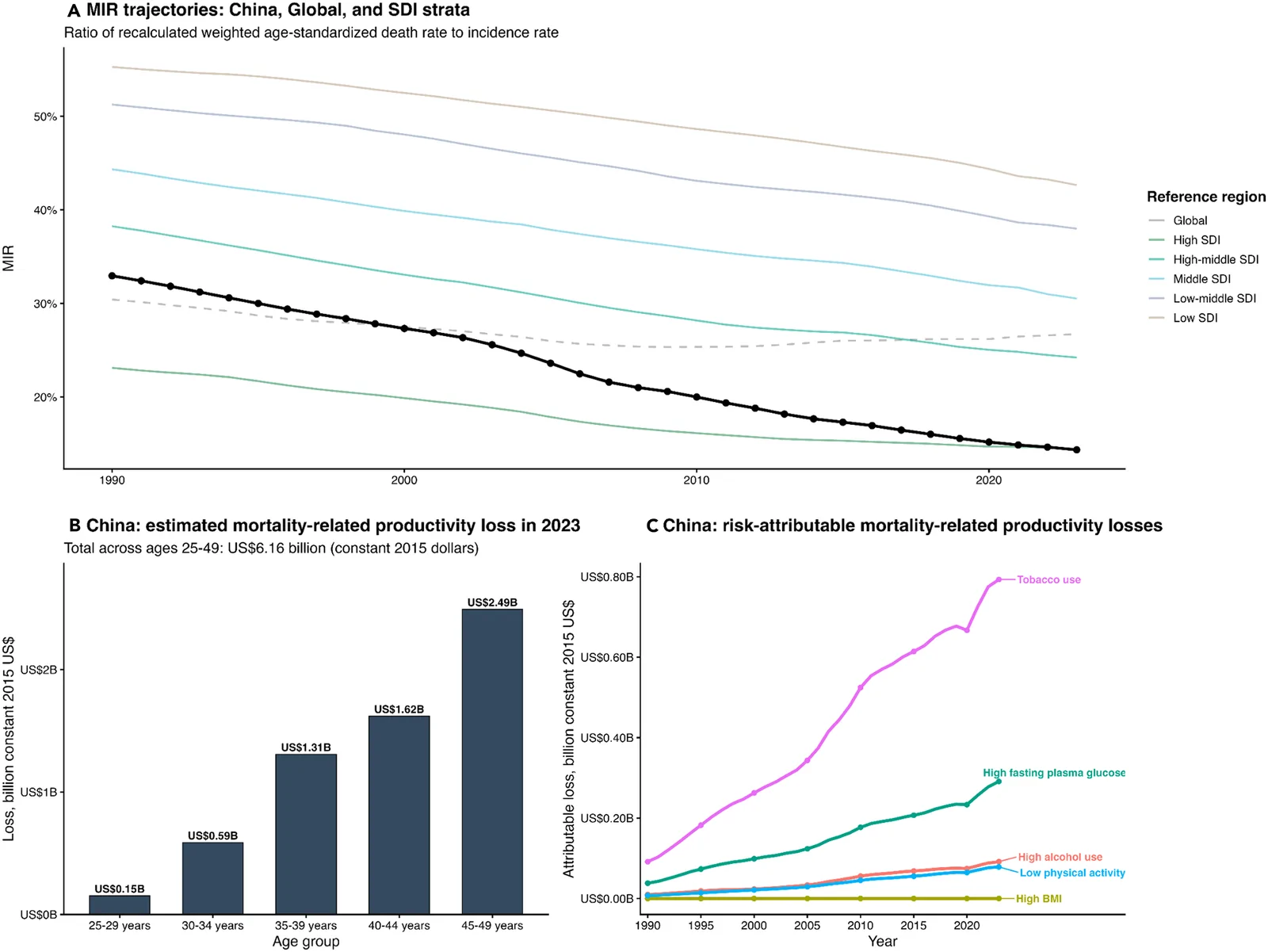
Mortality-to-incidence ratios and mortality-related productivity losses (A) Weighted MIR trajectories among women aged 25–49 years in China, the global population, and the five SDI strata. China is shown as a prominent black line with points, and the global population and SDI strata are shown as reference lines. MIR was calculated as weighted ASDR divided by weighted ASIR and should not be interpreted as an estimate of individual survival. (B) Age-specific productivity losses associated with premature breast-cancer mortality in China in 2023. (C) Risk-attributable productivity losses calculated using YLL-specific PAFs. Monetary values are reported in constant 2015 US dollars. MIR, mortality-to-incidence ratio; ASDR, age-standardized death rate; ASIR, age-standardized incidence rate; SDI, Socio-demographic Index; YLL, years of life lost; PAF, population attributable fraction; BMI, body mass index.

Premature breast-cancer mortality in China was associated with an estimated potential mortality-related productivity loss of US$6.161 billion in constant 2015 dollars in 2023 under the study’s Human Capital Approach assumptions. Age-specific losses increased from US$0.152 billion at ages 25–29 years to US$2.493 billion at ages 45–49 years. This age gradient primarily reflected the substantially larger aggregate YLL burden in the older age groups, rather than a higher productivity value assigned to older women. Tobacco use accounted for the largest risk-attributable loss (US$0.793 billion), followed by high FPG (US$0.291 billion), high alcohol use (US$0.092 billion), and low physical activity (US$0.079 billion); the high-BMI estimate was zero according to the source PAF series. Uniform 20% lower and higher productivity value scenarios produced total losses of US$4.929 billion and US$7.393 billion, respectively. The total-normalized age-graded scenario preserved the US$6.161 billion total while redistributing losses across age groups (Figures 4B, 4C, and S6 and Tables S4 and S13).

### Projections to 2050 and external comparison

To project future incidence, a Bayesian age-period-cohort model was applied. Under the 2023-anchored status-quo scenario, the posterior mean overall ASIR among Chinese women aged 25–49 years was 32.87 (95% posterior UI, 32.54–33.21) per 100,000 in 2023 and increased to 38.01 (95% posterior UI, 26.29–52.59) in 2030, 51.90 (95% posterior UI, 12.37–143.82) in 2040, and 106.87 (95% posterior UI, 4.23–551.55) in 2050. Point estimates increased in each age group, with the highest projected rates among women aged 45–49 years. The uncertainty intervals widened markedly after 2030, especially by 2050 (Figure 5 and Table S5).

**Figure 5:**
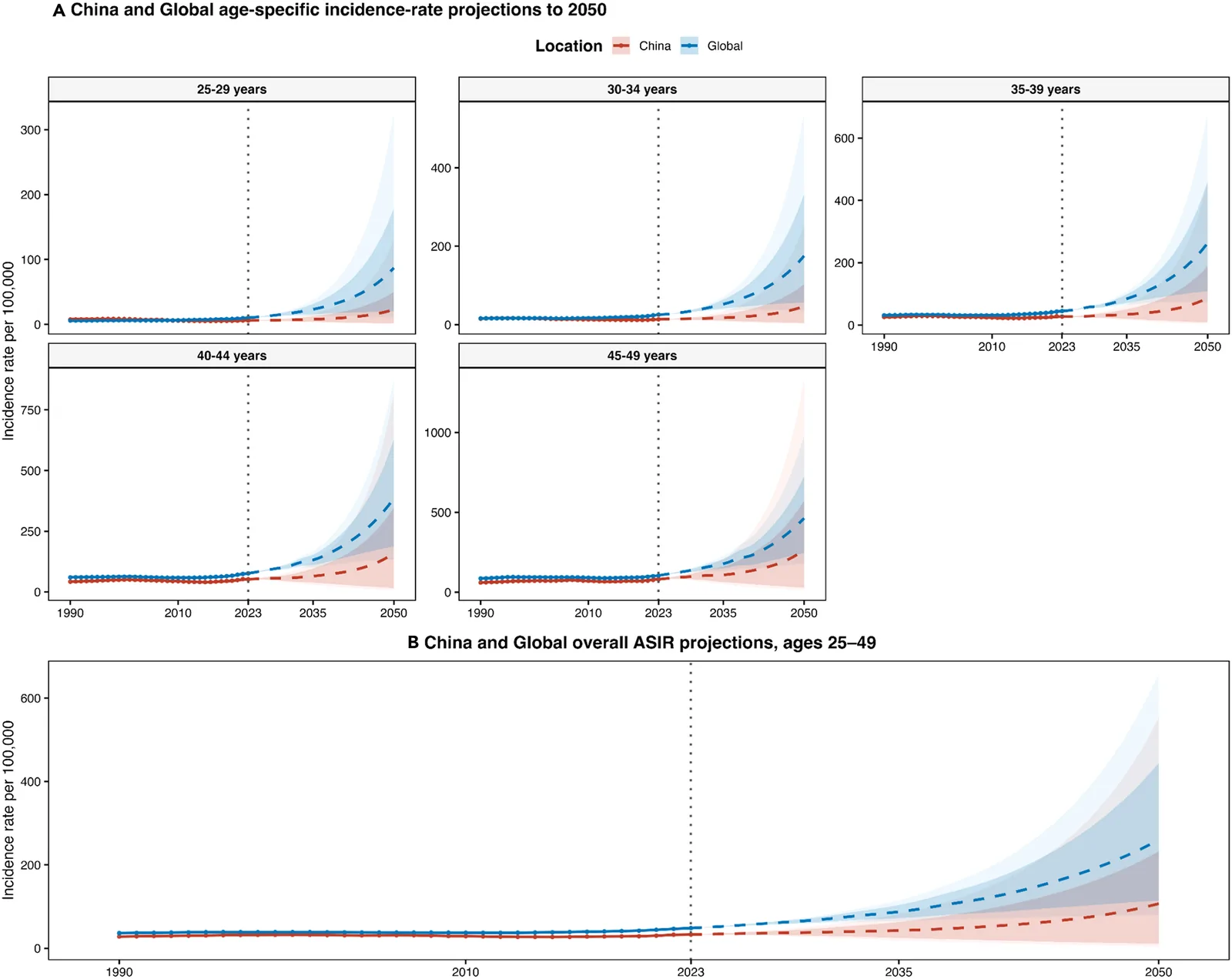
Status-quo Bayesian age-period-cohort projections of incidence to 2050 (A) China and global age-specific incidence-rate projections. (B) China and global overall weighted ASIR projections for ages 25–49 years. The vertical dotted line marks the 2023 transition from observed to projected years. Darker and lighter bands represent 80% and 95% posterior uncertainty intervals, respectively. Posterior means and interval bounds were multiplicatively anchored to the observed 2023 age-specific rates. Projections extrapolate historical age, period, cohort, and population patterns and are not deterministic forecasts. ASIR, age-standardized incidence rate.

Multiplicative anchoring to the observed 2023 boundary changed the China overall point estimates by less than 0.3% at each selected year. The anchored and unanchored 2050 estimates were 106.87 and 107.11 per 100,000, respectively, indicating that the boundary adjustment did not materially alter the projected direction or magnitude (Figure S4 and Table S11).

The GBD rates and rates derived from GLOBOCAN 2022 case estimates showed a strong correlation in age pattern across ten paired China and global age-specific estimates (Pearson *r* = 0.983). For China, the GBD rates were lower in all five age groups, with relative differences ranging from −13.0% at ages 25–29 years to −31.2% at ages 30–34 years. Global differences were smaller overall and ranged from −18.1% to +1.3% (Figure 6 and Table S1).

**Figure 6:**
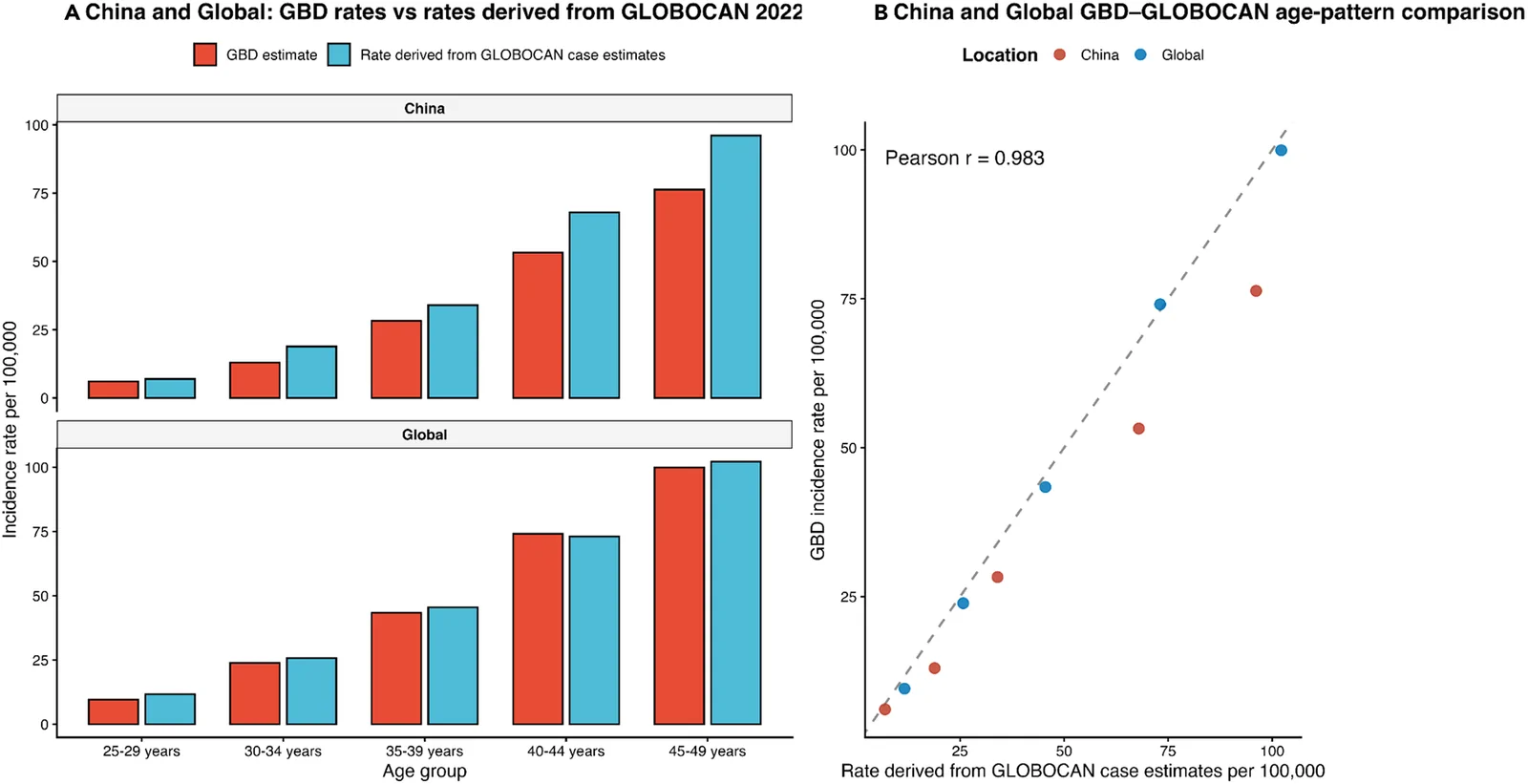
External comparison of GBD rates and rates derived from GLOBOCAN 2022 case estimates (A) Age-specific incidence rates among women aged 25–49 years in China and globally. Rates derived from GLOBOCAN case estimates were calculated using matched 2022 population denominators from the GBD-based analytic panel and are not official GLOBOCAN incidence rates. (B) China and global GBD-GLOBOCAN age-pattern comparison across ten paired estimates comprising five age groups for each location. The dashed line is the line of equality. Pearson’s correlation (*r* = 0.983) summarizes similarity in age pattern and does not imply agreement in absolute magnitude or formal validation of the projection model. GBD, Global Burden of Disease; GLOBOCAN, Global Cancer Observatory.

Applying the China age-specific case-estimate-derived-to-GBD baseline-scaling factors increased the projected overall ASIR by approximately 26.8% while preserving the increasing trajectory. The baseline-scaled point estimates were 48.17 (95% posterior UI, 33.33–66.65) per 100,000 in 2030, 65.80 (95% posterior UI, 15.68–182.34) in 2040, and 135.51 (95% posterior UI, 5.37–699.46) in 2050, compared with 38.01, 51.90, and 106.87 in the main GBD-based projection. These intervals were obtained by applying fixed scaling factors to the BAPC posterior bounds and did not incorporate uncertainty in the cross-source scaling factors themselves. The incidence baseline therefore affected the projected absolute level more than the direction of change (Figure S5 and Table S12).

## Discussion

Our findings indicate that the increase in breast cancer cases among Chinese women aged 25–49 years was not attributable to a single component. Incident cases increased between 1990 and 2023, whereas deaths and the weighted ASDR declined. Although the 2023 ASIR was higher than the 1990 value, the long-term ASIR trajectory was non-linear, and its EAPC was close to zero. Sequential decomposition identified the residual epidemiological component as the largest single component, but population growth and age-structure change together accounted for 63.0% of the additional cases. In the exploratory XGBoost-SHAP analysis, the FPG-related PAF was prominent in the primary model and several sensitivity formulations, whereas the high-BMI series was constant at zero and could not be ranked. Premature mortality was associated with substantial potential mortality-related productivity loss, and status-quo projections suggested a possible future increase in incidence with considerable long-term uncertainty.

The decomposition and cohort-specific descriptive results together help distinguish demographic contributions from broader temporal patterns. The increase in Chinese incident cases is consistent with the growing contribution of premenopausal and early-onset breast cancer to the global burden.^2^^,^^5^^,^^14^^,^^15^^,^^16^^,^^17^ However, the near-zero long-term ASIR EAPC shows that the endpoint increase did not represent a steady monotonic rise, and the decomposition does not support an exclusively epidemiological explanation because demographic changes jointly accounted for most of the additional cases. The residual epidemiological component captures the remaining change after the prespecified sequential replacement of population size and age structure and may reflect changing exposures, diagnostic intensity, registration completeness, health-care access, or other unmeasured influences. Because the allocation depends partly on replacement order, the 37.0% residual should be interpreted as a descriptive partition rather than a uniquely identified causal quantity. The cohort-specific patterns similarly describe observable trajectories but cannot separate age, period, and cohort effects, particularly because the more recent cohorts had not yet reached the older age groups.

The external comparison with GLOBOCAN adds a complementary source-based reference point. GBD and the rates derived from GLOBOCAN 2022 case estimates showed strong correlation in age pattern, but the GBD rates for China were 13.0%–31.2% lower across the five age groups. Such differences are plausible because GLOBOCAN emphasizes cancer-registry inputs, whereas GBD combines multiple sources within a broader disease-burden modeling framework.^1^^,^^14^^,^^15^^,^^18^^,^^19^^,^^20^^,^^21^ Applying the age-specific case-estimate-derived-to-GBD baseline-scaling factors increased the projected overall ASIR by approximately 26.8% without changing the upward direction. This cross-source difference does not establish that GBD underestimates the true incidence trajectory, because both sources are modeled and the future persistence of the 2022 ratios is uncertain. The baseline-scaled scenario held the source ratios constant through 2050, did not propagate uncertainty in the scaling factors, and was not an independent GLOBOCAN forecast or formal external validation. Similarly, 2023 anchoring changed the overall projection by less than 0.3%, indicating that boundary alignment was not responsible for the projected increase; the rapidly widening uncertainty intervals after 2030 remain the more important constraint on interpretation.

Recent GBD 2023 breast-cancer forecasts estimated female breast-cancer incidence and mortality through 2050 across 204 countries and territories using the GBD forecasting framework.^22^ That analysis covered the full female age range and both incidence and mortality, whereas the present study estimated incidence only among Chinese women aged 25–49 years using an author-implemented BAPC status-quo model with WPP 2024 population exposure, explicit 2023 anchoring, and an external-baseline scaling sensitivity analysis. Differences in absolute levels and trajectories may therefore reflect the different age scope, estimands, population inputs, and modeling frameworks, and the two sets of projections should not be treated as directly interchangeable.

The metabolic findings require cautious interpretation. High FPG ranked first in the primary absolute-DALY model and several sensitivity formulations, but high alcohol use ranked first in the earliest held-out period, and the FPG signal weakened after removal of age-group levels and smooth calendar-year trends. Thus, the FPG-related ranking was reproducible across several formulations but was not invariant to age and secular temporal structure. The selected high-BMI PAF series was zero in all 170 China year-age observations, preventing a valid FPG-versus-BMI comparison; this should not be interpreted as evidence that adiposity is biologically unimportant. Although the pattern is compatible with prior evidence linking diabetes, insulin resistance, inflammation, and hormonal pathways to breast cancer,^6^^,^^8^^,^^9^ the present analysis did not measure these exposures in individual women. Because SHAP values quantify contributions to model predictions rather than causal or mediated effects,^23^ the result should be regarded as a model-based, hypothesis-generating signal within a restricted set of GBD-derived PAF features.

The productivity-loss analysis adds a complementary perspective on burden in this age group. Under the Human Capital Approach,^3^^,^^4^ the estimated potential mortality-related productivity loss in China was US$6.161 billion in constant 2015 dollars in 2023 after accounting for age-specific female labor-force participation. The increase in age-specific loss from ages 25–29 to 45–49 years primarily reflected the larger aggregate YLL burden in the older age groups, rather than a higher productivity value assigned to older women. Importantly, the calculation used total GBD YLLs and did not truncate them at a prespecified retirement-age or working-age boundary; GBD YLLs represent years lost relative to standard life expectancy rather than observed years of employment. Uniform 20% changes in the value assigned to a productive life-year produced estimates from US$4.929 billion to US$7.393 billion, while the total-normalized age-graded scenario redistributed losses across age groups without changing the total. The use of national GDP per capita does not reproduce age- and occupation-specific wages, and the estimates exclude treatment expenditure, morbidity-related work loss, unpaid caregiving, household production, presenteeism, and quality-of-life effects. Because the Human Capital Approach can yield higher long-term estimates than friction-cost approaches that allow for worker replacement,^24^ the results should be interpreted as potential mortality-related productivity loss under the specified assumptions rather than observed labor-market earnings loss or the total economic burden of breast cancer.

For the analytic age group, the weighted MIR in China was 0.144 in 2023, and the crude deaths-to-incidence count ratio was 0.145. The two values were similar but represented different calculations. MIR can provide a population-level proxy when comparable survival data are unavailable, but it does not measure individual survival after diagnosis and cannot determine whether changes arise from earlier diagnosis, treatment, registration, health-care access, or other factors. The decline in the weighted MIR and ASDR therefore shows that the rise in incident cases was not accompanied by a parallel increase in the mortality rate, but the mechanisms behind this divergence cannot be identified from the present data.

The results do not justify a direct change in breast-cancer screening policy. This analysis did not compare screening thresholds, clinical benefit, overdiagnosis, individual risk discrimination, calibration, or cost-effectiveness. The first step would be individual-level prospective studies with baseline or repeated measurements of glucose-related biomarkers, BMI and body composition, and established reproductive, hormonal, genetic, dietary, and menopausal factors. These studies should evaluate confounding, mediation, and effect modification; tumor subtype could subsequently be examined as an outcome-heterogeneity characteristic among incident cases rather than as a pre-screening baseline predictor. If a prediction model is developed using variables available before screening, the incremental contribution of glucose-related markers should be assessed beyond established risk factors, followed by internal validation, evaluation of discrimination and calibration, and independent external validation. Such model-development and evaluation studies should be reported transparently,^25^ and clinical utility should then be assessed using decision-curve analysis,^26^ cost-effectiveness modeling, and prospective implementation or intervention studies before any change to targeted prevention or early-detection policy is considered.^27^^,^^28^

This study has several strengths. It used a common age-specific framework across burden estimation, decomposition, MIR, productivity loss, projection, and external comparison; recalculated weighted rates specifically for ages 25–49 years; and integrated population, labour-force, economic, and cancer-estimation sources. The revised analysis also included a complete missingness and variance audit, forward temporal validation, grouped temporal bootstrap resampling, multiple target and age-structure formulations, projection-boundary diagnostics, and an external-baseline sensitivity analysis. These additions distinguish results that were stable from those that depended on model structure or analytical assumptions.

### Limitations of the study

Several limitations should also be noted. First, GBD and GLOBOCAN are modeled population-level resources whose estimates depend on surveillance quality, data availability, redistribution procedures, and modeling assumptions.^1^^,^^14^^,^^15^^,^^18^^,^^19^^,^^20^^,^^21^^,^^29^ The age-specific lower and upper GBD bounds were aggregated using the same weights as the point estimates, but covariance among the underlying draws was not reconstructed. Second, the decomposition was sequential and order-dependent, and its residual component did not identify specific exposures or health-system mechanisms. The cohort analysis was descriptive rather than a formal age-period-cohort identification analysis. Third, the PAF feature set was narrow and omitted many factors relevant to breast cancer, including reproductive history, hormonal exposure, genetic susceptibility, menopausal status, tumor subtype, and dietary factors. The selected China high-BMI PAF series had zero variance, preventing a valid FPG-versus-BMI comparison; neither this source-data limitation nor the use of BMI as an imperfect adiposity proxy should be interpreted as evidence that obesity exposure or biological relevance was absent. SHAP values did not provide causal effects, mediation estimates, or validated individual prediction.^23^

Fourth, MIR was a descriptive proxy rather than a survival measure. The Human Capital Approach captured only mortality-related productivity loss, and risk-attributable estimates inherited uncertainty and potential overlap from the GBD PAFs. Total GBD YLLs were not truncated at a prespecified working-age or retirement-age boundary, so some years included in the valuation may extend beyond conventional working ages; the resulting estimates may therefore overstate strict labor-market productivity loss and should be interpreted as potential loss under the stated Human Capital Approach assumptions. Fifth, the Bayesian age-period-cohort model extrapolated historical age, period, cohort, and population patterns under a status-quo scenario.^30^^,^^31^ It did not explicitly model future changes in screening, treatment, reproductive patterns, metabolic exposures, or registration. Although anchoring had little effect on point estimates, the projection intervals became extremely wide at longer horizons, and the GLOBOCAN-based baseline-scaled results remained a sensitivity scenario rather than an independent forecast. The scaled intervals did not incorporate uncertainty in the GLOBOCAN-to-GBD scaling factors. These limitations preclude individual-level causal inference and require the 2050 estimates to be interpreted as scenario ranges rather than expected outcomes.

In conclusion, among Chinese women aged 25–49 years, incident breast cancer cases increased between 1990 and 2023 while the mortality rate declined. Population growth and age-structure change jointly explained most of the additional cases, with a residual epidemiological component accounting for the remainder. The FPG-related PAF was a prominent exploratory feature across several model formulations, but its ranking was partly dependent on age and secular temporal structure; the constant-zero high-BMI series did not permit a valid comparison between glucose- and adiposity-related effects. Under the specified Human Capital Approach assumptions, premature mortality was associated with substantial potential mortality-related productivity loss. Status-quo projections indicated a possible future increase in incidence, although absolute levels were sensitive to the incidence baseline and long-term uncertainty was considerable. These findings support continued age-specific surveillance and individual-level validation of metabolic markers rather than immediate changes to screening policy.

## Resource availability

### Lead contact

Further information and requests for resources should be directed to and will be fulfilled by the lead contact, Jian Zhang (zjxjtu14@163.com; ORCID: 0000-0003-1144-3627).

### Materials availability

This study did not generate new unique reagents or materials.

### Data and code availability

• The source datasets analyzed in this study are publicly available from the Global Health Data Exchange, Global Cancer Observatory, United Nations World Population Prospects, International Labour Organization, and World Bank. The corresponding sources, indicators, access information, and expected input files are described in the key resources table and the accompanying source-data documentation. Original third-party source files are not redistributed because they remain subject to the access and licensing conditions of their respective providers.
• The complete standalone R analysis pipeline, author-derived tables, figure source data, software-environment information, and quality-control outputs are publicly available in the associated GitHub repository at https://github.com/baiyangxjtu-sys/breast-cancer-burden-risk-attribution-productivity-loss-china-25-49 (release v1.1.10). A frozen reproducibility package for analysis version v1.1.10 is publicly archived on Figshare (https://doi.org/10.6084/m9.figshare.33467353) and is also supplied with this article as Data S1.
• Any additional information required to reanalyze the results reported in this paper is available from the lead contact upon reasonable request.

## Acknowledgments

This work was supported by the Clinical Research Fund of the 10.13039/100031934First Affiliated Hospital of Xi'an Jiaotong University (grant nos. HXIIT2025140 and XJTU1AF-CRF-2023-027) and in part by the Key R&D Program of Shaanxi Province (grant no 2026SF-YBXM-024 to Jian Zhang). The funders had no role in study design, data collection, analysis, interpretation, or manuscript preparation.

## Author contributions

Y.B.: Conceptualization, methodology, software, formal analysis, data curation, visualization, and writing – original draft. J.Z.: validation, data curation, and writing – review and editing. T.T.: validation and writing – review and editing. K.W.: conceptualization, supervision, project administration, funding acquisition, and writing – review and editing. J.Z.: conceptualization, supervision, funding acquisition, and writing – review and editing, lead contact.

## Declaration of interests

The authors declare no competing interests.

## Declaration of generative AI and AI-assisted technologies in the manuscript preparation process

During the preparation of this work, the authors used ChatGPT (OpenAI) for language refinement and manuscript organization. The authors reviewed and edited all outputs and take full responsibility for the manuscript content.

## STAR★Methods

### Key resources table

**Table:** 

REAGENT or RESOURCE	SOURCE	IDENTIFIER
**Deposited data**		

GBD 2023 breast-cancer burden estimates	Institute for Health Metrics and Evaluation	Global Health Data Exchange (GHDx); https://ghdx.healthdata.org/gbd-2023; downloaded May 12, 2026
GLOBOCAN 2022 age-specific case estimates	International Agency for Research on Cancer	Cancer Today, Global Cancer Observatory; https://gco.iarc.who.int/today; accessed April 7, 2026
World Population Prospects 2024	United Nations	Medium variant; https://population.un.org/wpp/; accessed April 7, 2026
Female labour-force participation rates	International Labour Organization	ILOSTAT; labour force participation rate by sex and age; accessed April 7, 2026
GDP per capita, constant 2015 US$	World Bank	World Development Indicators; indicator NY.GDP.PCAP.KD; downloaded May 12, 2026
Frozen reproducibility package for analysis version v1.1.10	This paper	Figshare: https://doi.org/10.6084/m9.figshare.33467353; also supplied with the article as Data S1
Analysis code and author-derived outputs	This paper	GitHub: https://github.com/baiyangxjtu-sys/breast-cancer-burden-risk-attribution-productivity-loss-china-25-49; release v1.1.10

**Software and algorithms**		

R	R Foundation for Statistical Computing	Version 4.5.1
tidyverse	CRAN	Version 2.0.0
dplyr	CRAN	Version 1.2.1
tidyr	CRAN	Version 1.3.1
forecast	CRAN	Version 9.0.2
INLA	R-INLA	Version 25.10.19
xgboost	CRAN	Version 3.2.1.1
shapviz	CRAN	Version 0.10.3
ggplot2	CRAN	Version 4.0.1
patchwork	CRAN	Version 1.3.2
cowplot	CRAN	Version 1.2.0
openxlsx	CRAN	Version 4.2.8

### Experimental model and study participant details

#### Study design and analytic population

We extracted incident cases, prevalent cases, deaths, disability-adjusted life years (DALYs), years of life lost (YLLs), years lived with disability (YLDs), and age-specific rates for breast cancer from the Global Burden of Disease Study 2023 through the Global Health Data Exchange (GHDx; https://ghdx.healthdata.org/; downloaded May 12, 2026).^18^ All data used in this study were aggregated population-level estimates; no individual-level exposure, clinical, or outcome data were available. Breast cancer was defined using ICD-10 code C50 and related subcodes. The analysis was restricted to women aged 25–49 years and divided into five 5-year age groups: 25–29, 30–34, 35–39, 40–44, and 45–49 years. Analyses were performed for China, the global population, and the five global Socio-demographic Index (SDI) strata. SDI is a composite measure based on income per capita, educational attainment, and fertility among women younger than 25 years.^18^ The SDI strata were included as global reference groups across levels of sociodemographic development and did not represent subnational regions within China.

All analyses used publicly available, aggregated, de-identified population-level estimates. The study did not involve recruitment, intervention, individual-level records, human tissue, or identifiable personal information; therefore, institutional ethics approval and informed consent were not required.

### Method details

#### Data sources and rate construction

Absolute numbers of incident cases, prevalent cases, deaths, DALYs, YLLs, and YLDs were aggregated across the five age groups. Because the analysis was restricted to ages 25–49 years, we recalculated age-standardized rates for this analytic age range using age-specific rates and GBD standard population weights, rather than relying on all-age standardized rows. The weights for the five age groups 25–29, 30–34, 35–39, 40–44, and 45–49 years were 0.0807, 0.0754, 0.0699, 0.0645, and 0.0585, respectively. The weighted age-standardized rate was calculated as:

ASR(t)=Σ[w(a)×r(a,t)]/Σw(a)

where r(a,t) denotes the age-specific rate in age group a and year t, and w(a) denotes the corresponding standard population weight. The same summation or weighting rules were applied to the point estimates and the reported lower and upper GBD uncertainty bounds; covariance among age-specific draws was not reconstructed. The maps in Figure 1 used the GBD all-age age-standardized rows for descriptive context only, whereas all longitudinal and analytical results were based on women aged 25–49 years. Values in Table 1 were therefore recalculated by the authors from age-specific GBD estimates rather than copied from the all-age summary rows.

Historical age-specific population estimates used in the decomposition and Bayesian age-period-cohort analyses were reconstructed from the matched GBD number and rate estimates. Future female population estimates for China were obtained from the United Nations World Population Prospects 2024,^32^ whereas future global age-specific populations were extrapolated from the historical reconstructed series. Female labour-force participation rates from the International Labour Organization and GDP per capita from the World Bank were used in the productivity-loss analysis.^33^^,^^34^ GLOBOCAN 2022 age-specific case estimates obtained from Cancer Today, Global Cancer Observatory, were used for external comparison of the 2022 incidence baseline.^1^^,^^19^ A summary of data provenance and the derivation of key study outputs is provided in Table S17.

#### Decomposition analysis

We decomposed the change in absolute incident cases between 1990 and 2023 into three components: population growth, change in age structure, and a residual epidemiological component, defined as the remaining change after sequentially accounting for population size and age structure. A prespecified sequential replacement approach adapted from the decomposition framework described by Das Gupta^12^^,^^13^ was used, with total population size replaced first and age structure replaced second. The analysis was conducted for China, the global population, and each of the five SDI strata using the same five age groups. For each age group, the population size was reconstructed from GBD estimates as:

P(a,t)=N(a,t)/[r(a,t)/100,000]

where P(a,t) is the estimated female population in age group a and year t, N(a,t) is the number of incident cases, and r(a,t) is the age-specific incidence rate per 100,000 population.

Total incident cases in 1990 and 2023 were calculated as:

C(1990)=ΣN(a,1990),C(2023)=ΣN(a,2023)

The counterfactual number of cases expected under population growth alone was estimated as:

C(pop)=C(1990)×P(2023)/P(1990)

where P(1990) and P(2023) are the total female populations aged 25–49 years in 1990 and 2023, respectively. The number of cases expected after additionally accounting for the 2023 age structure while retaining the 1990 age-specific incidence rates was estimated as:

C(dem)=Σ[P(a,2023)×r(a,1990)/100,000]

The three components were then calculated as:

 ΔC(population) = C(pop) − C(1990)

 ΔC(age structure) = C(dem) − C(pop)

 ΔC(residual epidemiological) = C(2023) − C(dem)

Component proportions were calculated by dividing each component by the total change in incident cases, C(2023) - C(1990). A negative value indicated that the corresponding component offset part of the total increase. Because sequential decomposition depends partly on the specified replacement order, the components were interpreted as descriptive partitions rather than uniquely identified causal quantities. The residual epidemiological component represents the remaining change after accounting for population size and age structure and may include changes in exposures, diagnostic intensity, registration completeness, health-care access, or other unmeasured influences. It was not interpreted as a directly observed causal or metabolic mechanism and was not further allocated among the five selected GBD risk-specific PAFs.

#### Cohort-specific descriptive analysis

To describe cohort-specific incidence patterns, birth year was approximated by subtracting the midpoint of each 5-year age group from the calendar year:

b(a,t)=t−m(a)

where b(a,t) is the estimated birth year, t is calendar year, and m(a) is the midpoint of age group a. Age midpoints were defined as 27, 32, 37, 42, and 47 years for the five age groups 25–29, 30–34, 35–39, 40–44, and 45–49 years, respectively. Birth cohorts were grouped as the 1960s, 1970s, 1980s, and 1990 or later. Cohorts born before 1960 were not included in Figure 2C because they were not observed across the younger analytic age groups during the 1990–2023 study period. For each cohort group and age stratum, mean age-specific incidence rates were calculated across the observable study years. No interpolation or extrapolation was used for unobserved cohort-age combinations; consequently, later cohorts contributed fewer older age groups because follow-up ended in 2023. We used this step only to describe cohort-specific age patterns and not to estimate independent age, period, or cohort effects.

#### Risk attribution and exploratory feature ranking

Population attributable fractions (PAFs) from the GBD Comparative Risk Assessment framework were used to summarize selected risk-related burdens.^29^ Three broad risk categories—behavioural, dietary, and metabolic risks—and five specific factors were examined descriptively. The evaluated factors were high alcohol use, high body mass index (BMI), high fasting plasma glucose (FPG), low physical activity, and tobacco use. The broad temporal trends and age-specific heatmaps were based on DALY-specific PAFs only. For the broad temporal trends, the five age-specific PAF estimates were summarized using their unweighted arithmetic mean for each year and location. This value was used only as a descriptive summary across the five age groups and should not be interpreted as a population-weighted or overall PAF for women aged 25–49 years. Signed PAF values were retained in the heatmaps.

For the exploratory feature-ranking analysis, the unit of observation was a year-age-group combination among Chinese women aged 25–49 years, yielding 170 observations. Risk-specific DALY PAFs for the five prespecified factors were initially considered as input features, and the absolute number of DALYs was used as the target. Before model fitting, all year-age-group combinations were audited for missing values, duplicate keys, observed zeros, and feature variance. All 170 observations had complete target and feature data, and no imputation was performed. The selected China-specific GBD breast-cancer DALY PAF series for high BMI was exactly zero in all 170 observations and had zero variance; it was retained in the descriptive analysis but excluded from the XGBoost-SHAP ranking. This constant-zero pattern was a property of the selected GBD risk-attribution series and was not an estimate of high-BMI prevalence among Chinese women. The final feature matrix was therefore defined as:

X(i)=[x(i,1),x(i,2),x(i,3),x(i,4)]

where i denotes a year-age-group observation and the four nonconstant features represented PAFs for high alcohol use, high FPG, low physical activity, and tobacco use. The target variable was defined as:

Y(i)=DALYs(i)

We used XGBoost regression to compare the relative importance of the four nonconstant PAF variables. This step was intended for exploratory feature ranking rather than for developing a validated prediction model.^35^ The model was fitted using a squared-error objective, with a maximum tree depth of 4, learning rate of 0.05, and 150 boosting iterations. These hyperparameters were fixed in the analysis pipeline and applied unchanged across the primary model, temporal folds, and sensitivity formulations. No hyperparameter tuning, early stopping, or model selection was performed using the held-out future periods. A fixed random seed of 2026 was used for reproducibility. The full 170-observation panel was used for the primary ranking, and the target was divided by 1,000 for graphical presentation.

SHapley Additive exPlanations (SHAP) values were calculated from the fitted XGBoost model to summarize the direction and magnitude of each feature’s contribution to model-predicted DALYs.^36^ Feature importance was summarized using the mean absolute SHAP value, excluding the XGBoost baseline contribution. SHAP values were expressed as contributions to model-predicted DALYs in thousands and were interpreted as model-based feature contributions rather than causal effects, independent effects, or mediation estimates.^23^ The magnitude of a source PAF and its SHAP-based importance were treated as different quantities.

Temporal stability was examined using three forward validation analyses: models trained on 1990–2005, 1990–2011, and 1990–2017 were evaluated in 2006–2011, 2012–2017, and 2018–2023, respectively. The model specification and four-feature set were unchanged across folds. For each fold, predictive performance and SHAP importance were calculated exclusively in the held-out future-period observations. Held-out predictive performance was summarized using root mean squared error, mean absolute error, R^2^, and Spearman correlation between observed and model-predicted DALYs. Separately, concordance of the SHAP feature ranking in each temporal fold with the full-panel feature ranking was assessed using Kendall’s tau. Because the analytic panel contained temporal and within-year dependence, structured validation and block resampling were used instead of random observation-level cross-validation.^37^ The 1990–2023 series was partitioned into six complete non-overlapping 5-year calendar blocks and one final 4-year block covering 2020–2023. In each of 200 resamples, seven blocks were sampled with replacement, all five age groups within each selected year were retained, the XGBoost model was refitted, and SHAP importance was evaluated on the fixed full-panel feature matrix to permit comparison across resamples. Normalized SHAP importance, feature ranks, top-ranked frequencies, and Kendall’s tau for ranking concordance were then summarized.

To assess dependence on population size, age-group differences, and long-term temporal structure, the analysis was repeated using five formulations: absolute DALYs, age-specific DALY rates, DALY rates and PAFs centered within age group, within-age-group annual first differences, and residuals after adjustment for age-group indicators and a natural spline for calendar year with 4 degrees of freedom. Each formulation used the same fixed XGBoost parameters and 200 grouped calendar-block bootstrap resamples. These analyses were used to assess specification dependence and were not interpreted as causal adjustment or validation of individual risk prediction.

#### Mortality-to-incidence ratio

For each location and year, the mortality-to-incidence ratio (MIR) for women aged 25–49 years was calculated as the recalculated weighted age-standardized death rate divided by the recalculated weighted age-standardized incidence rate:

MIR(t)=ASDR25−49(t)/ASIR25−49(t)

The crude deaths-to-incidence count ratio was calculated separately from aggregated death and incident-case numbers. The weighted MIR was used in Figure 4 to maintain a consistent age range and rate denominator. Neither measure was interpreted as an estimate of individual survival.

#### Mortality-related productivity loss

Productivity losses associated with premature breast-cancer mortality were estimated using the Human Capital Approach.^3^^,^^4^ In this study, premature mortality refers to breast-cancer deaths among women aged 25–49 years as represented by GBD YLLs, which quantify years of life lost relative to the GBD standard life expectancy. These YLLs represent years of life lost rather than observed years of employment or working life. For each year and age group, breast-cancer-related total YLLs were multiplied by GDP per capita and the corresponding age-specific female labour-force participation rate. The total GBD YLLs used in this calculation were not truncated at a prespecified retirement-age or working-age boundary; therefore, some YLLs may extend beyond conventional working ages. The age-specific productivity loss was calculated as:

L(a,t)=YLL(a,t)×GDPpc(t)×LFPR(a,t)

where L(a,t) is the estimated mortality-related productivity loss for age group a in year t, YLL(a,t) denotes years of life lost, GDPpc(t) denotes GDP per capita, and LFPR(a,t) denotes the age-specific female labour-force participation proportion. Annual GDP per capita was obtained from World Bank indicator NY.GDP.PCAP.KD and was expressed in constant 2015 US dollars.^34^ Age-specific female labour-force participation rates were obtained from the International Labour Organization.^33^ When annual values were unavailable, linear interpolation was applied within each age group, with the nearest observed value extended to boundary years when required.

Total productivity loss among women aged 25–49 years was calculated as:

L(t)=ΣL(a,t)

Risk-attributable productivity losses were estimated using YLL-specific PAFs, which were complete for all required year-age-group-risk combinations. DALY-specific PAFs were not substituted. The risk-specific loss was calculated as:

L(a,t,k)=L(a,t)×PAFYLL(a,t,k)

where PAFYLL(a,t,k) represents the YLL-specific population attributable fraction for risk factor k. Risk-specific losses were summed across age groups but were reported separately and not summed across risk factors because attributable fractions may overlap. Signed PAFs were retained in the source audit; under the prespecified monetary-loss rule, any negative PAF would be set to zero only for the monetary calculation.

Sensitivity analyses varied the monetary value assigned to one productive life-year by ±20%. An illustrative age-graded profile was also constructed using raw scenario multipliers of 0.75, 0.90, 1.05, 1.10, and 1.00 across the five age groups. These raw values were illustrative assumptions rather than empirical age-specific female wage estimates. The applied age multipliers were normalized using the 2023 main-loss distribution so that the age-graded scenario preserved the overall 2023 monetary scale while redistributing losses across age groups. Combined age-graded and ±20% scenarios were also examined. These scenarios were treated as assumption bounds rather than confidence intervals.

These estimates were interpreted as approximations of potential mortality-related productivity loss under the specified Human Capital Approach assumptions rather than observed labour-market earnings losses or comprehensive societal cost estimates, because treatment expenditures, morbidity-related work loss, unpaid caregiving, household production, presenteeism, and quality-of-life losses were not included.

#### Bayesian age–period–cohort projection and external comparison

We projected future incidence through 2050 using a Bayesian age-period-cohort model implemented with Integrated Nested Laplace Approximations.^30^^,^^31^ Historical age-specific incident cases and reconstructed population estimates from 1990 to 2023 were used to fit the model. Future female population estimates for China from 2024 to 2050 were obtained from the United Nations World Population Prospects 2024.^32^ This source was used because it provides an internally consistent annual age-specific female population series through 2050; Chinese census observations are available only at discrete time points, whereas life tables describe mortality and survival rather than annual population exposure. For the global population, future age-specific population counts were extrapolated using automatic ARIMA models fitted to the historical reconstructed population series.

For each location, age group, and year, incident cases were modelled using a Poisson likelihood:

Y(a,t)∼Poisson[P(a,t)×λ(a,t)]

where Y(a,t) is the number of incident cases, P(a,t) is the corresponding population, and λ(a,t) is the age-specific incidence rate. The linear predictor was specified as:

log[λ(a,t)]=α+γ(a)+π(t)+κ(c)

where α is the intercept, γ(a) denotes the age effect, π(t) denotes the period effect, and κ(c) denotes the cohort effect. Cohort was indexed according to calendar year minus the lower bound of the corresponding age group. The age effect was assigned an independent and identically distributed prior, whereas period and cohort effects were assigned scaled second-order random-walk priors. The model used a Poisson likelihood with population as the exposure term.

Posterior predictive distributions were summarized using the mean and selected quantiles. The main results reported 80% and 95% posterior uncertainty intervals, while additional nested intervals were retained for diagnostic fan charts. The projections represented a status-quo scenario based on extrapolation of historical age, period, cohort, and population patterns and did not explicitly model future changes in screening, treatment, reproductive patterns, risk exposures, or registration.

To make the projected curves connect with the observed 2023 boundary while preserving uncertainty, a separate multiplicative anchor ratio was calculated for each location-age series:

Anchorratio(l,a)=observedrate(l,a,2023)/unanchoredfittedrate(l,a,2023)

For 2023 onward, this ratio was applied to the posterior mean and all posterior interval bounds. Unanchored projections were retained as a sensitivity analysis. Age-standardized incidence rates for the 25–49-year group were calculated as weighted averages of the age-specific projected rates using the same GBD standard population weights as in the historical analysis.

Model estimates for 2022 were externally compared with age-specific GLOBOCAN 2022 case estimates for China and the global population. To place the case estimates on a common population scale, comparison rates were derived using the matched 2022 population denominators reconstructed from the GBD analytic panel. These values were therefore rates derived from GLOBOCAN case estimates rather than official GLOBOCAN incidence rates. Absolute and relative differences were reported, and similarity in age pattern was summarized graphically and using Pearson correlation across ten paired estimates—five age groups for China and five for the global population. This comparison was used as an external baseline reference and not as formal validation of the Bayesian age-period-cohort model.

For the external-baseline scaling sensitivity analysis, an age- and location-specific baseline-scaling factor was calculated as the rate derived from GLOBOCAN 2022 case estimates divided by the corresponding GBD 2022 rate. Each factor was held constant from 2023 through 2050 and applied to the posterior mean and interval bounds. The baseline-scaled overall ASIR was then recalculated using the standard population weights. This scenario changed the baseline magnitude while preserving the GBD-derived relative trajectory and was not treated as an independent GLOBOCAN forecast. The scaled intervals retained the relative posterior uncertainty of the main BAPC model but did not incorporate uncertainty in the GLOBOCAN-to-GBD scaling factors themselves.

### Quantification and statistical analysis

Estimated annual percentage changes (EAPCs) were calculated by fitting log-linear models to weighted age-standardized rates over calendar year. If β denotes the regression coefficient for year, EAPC was calculated as:

EAPC=100×[exp(β)−1]

The 95% confidence interval was derived from the standard error of β. As the analyses were mainly descriptive, model-based, and exploratory, P-value thresholds were not used as decision criteria.

All analyses were performed in R version 4.5.1 on macOS. Key packages included tidyverse 2.0.0, dplyr 1.2.1, tidyr 1.3.1, forecast 9.0.2, INLA 25.10.19, xgboost 3.2.1.1, shapviz 0.10.3, ggplot2 4.0.1, patchwork 1.3.2, cowplot 1.2.0, and openxlsx 4.2.8. A fixed random seed of 2026 was used for XGBoost analyses. The final standalone analysis pipeline was frozen as version 1.1.10 and included automated checks for missingness, duplicate keys, zero-variance features, source-data completeness, temporal-fold separation, calendar-block resampling, projection anchoring, interval ordering, baseline-scaling terminology, HCA PAF handling, and manuscript-output consistency.

## Supplemental information

Document S1. Figures S1–S6 and Tables S1–S17

Data S1. Frozen reproducibility package for analysis version v1.1.10
