# Supplementary material for: Breast cancer burden risk attribution and productivity loss in Chinese women aged 25 to 49
Source: iScience. 2026 Oct 14;29(11):117891. doi: 10.1016/j.isci.2026.117891 (PMC13683865; doi:10.1016/j.isci.2026.117891)
Supplement: Supplementary file 1 [file mmc1.pdf]

iScience, Volume *29*

Supplemental information

Breast cancer burden risk attribution and productivity loss in Chinese women aged 25 to 49

Yang Bai, Jiaqi Zhang, Tian Tian, Ke Wang, and Jian Zhang

Supplementary Figures

Supplementary Figure S1.

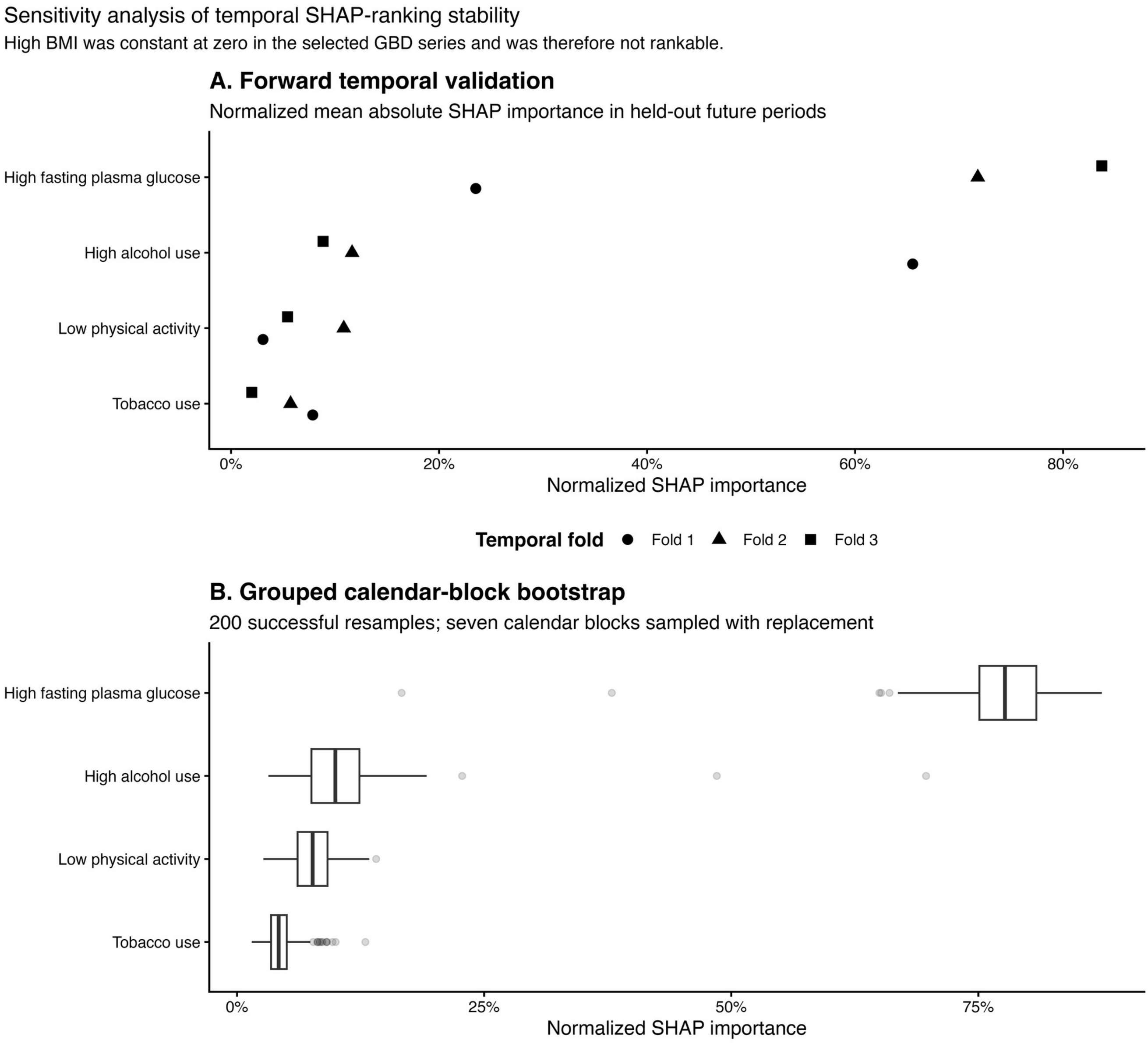

Supplementary Figure S1. Temporal stability of the exploratory SHAP ranking. Fold-specific SHAP values were calculated exclusively in held-out future-period observations. The grouped bootstrap sampled six non-overlapping 5-year blocks and one final 4-year block with replacement, retaining all five age groups within each selected year. High BMI was constant at zero in the selected GBD series and was therefore not rankable; this does not indicate absent obesity exposure or biological irrelevance.

Supplementary Figure S2.

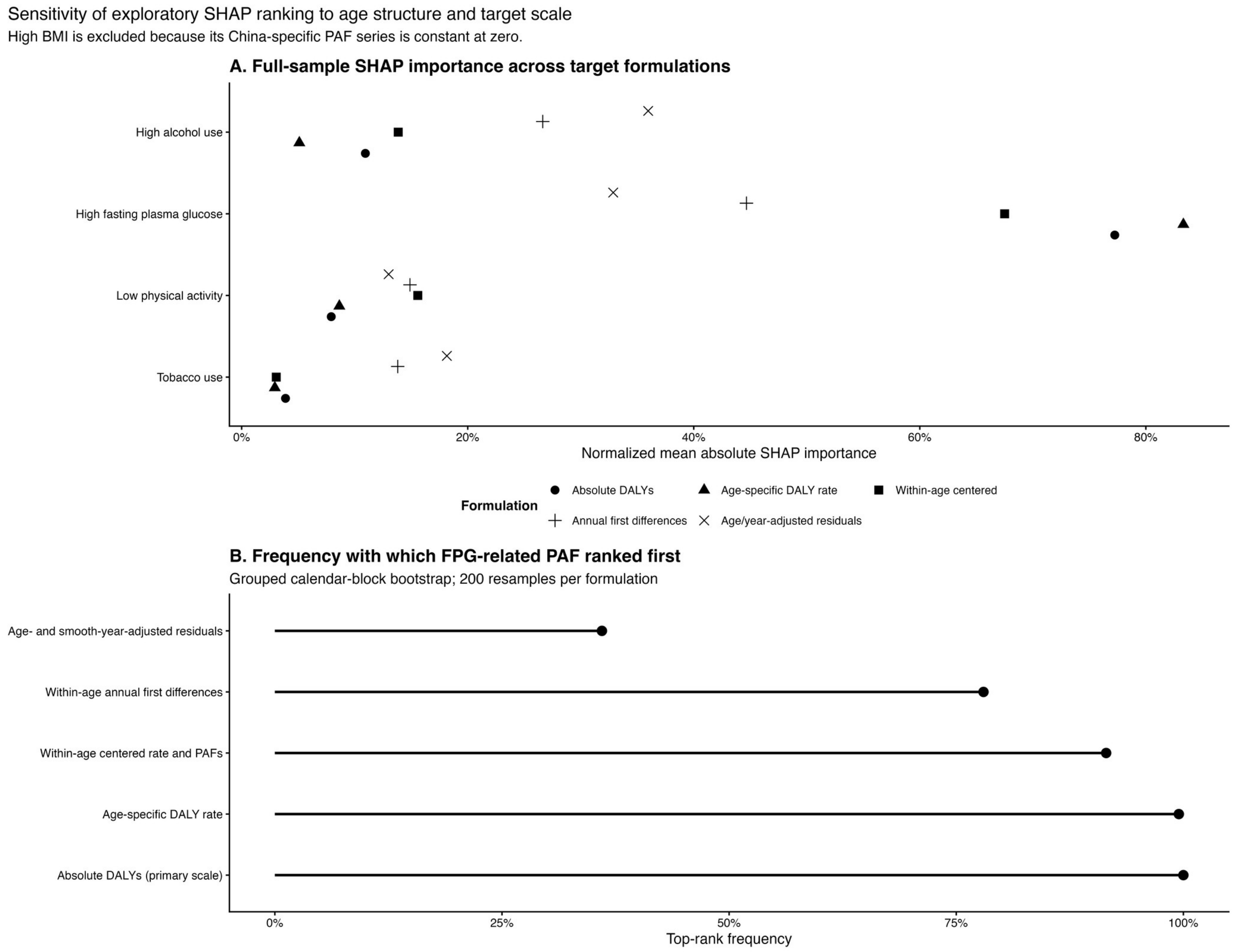

Supplementary Figure S2. Sensitivity of the exploratory SHAP ranking to target scale, population size, between-age differences, annual changes, and residual age/time structure.

Supplementary Figure S3.

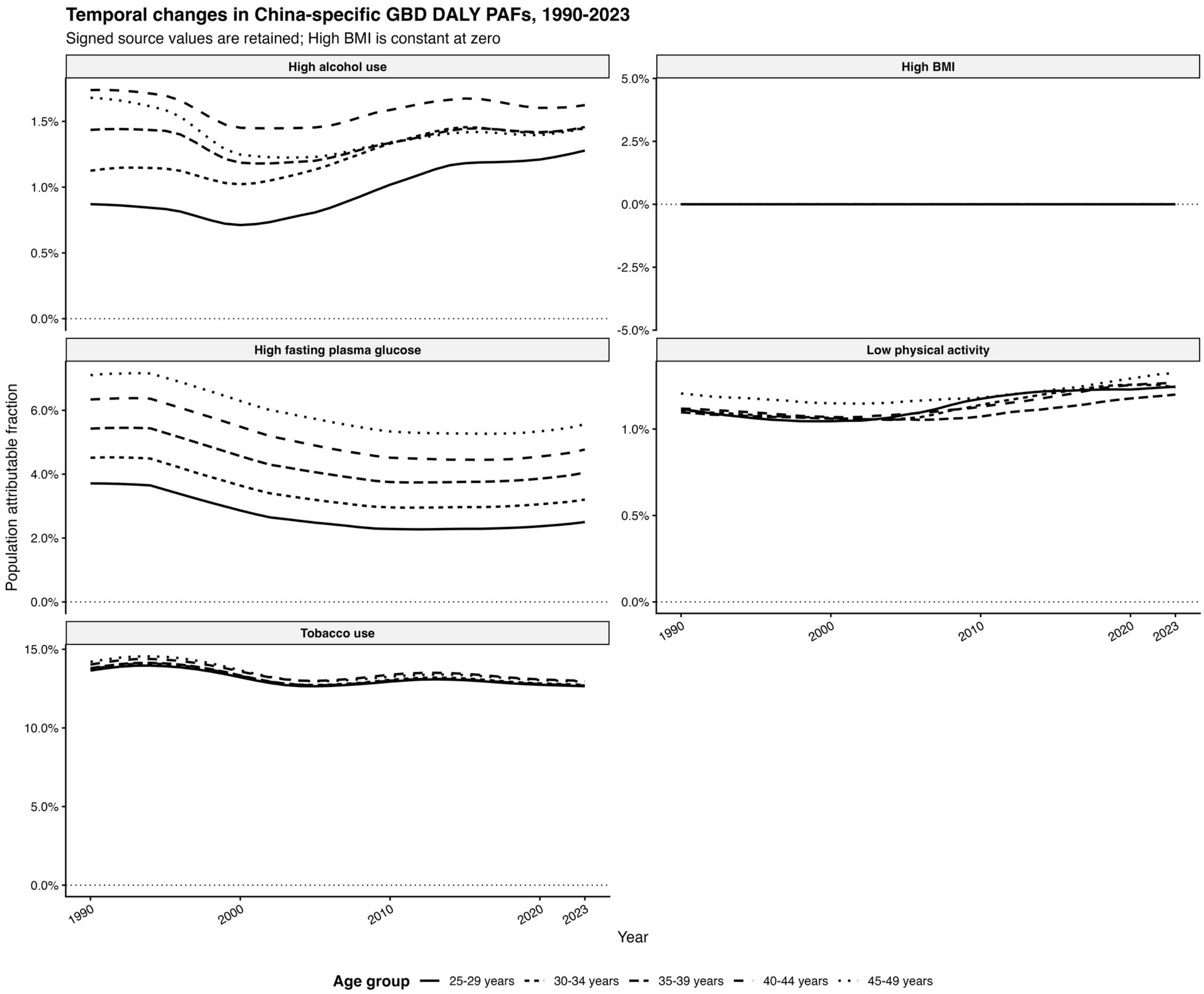

Supplementary Figure S3. Age-specific temporal patterns in signed China-specific GBD DALY PAFs from 1990 to 2023. The High BMI series is constant at zero.

Supplementary Figure S4.

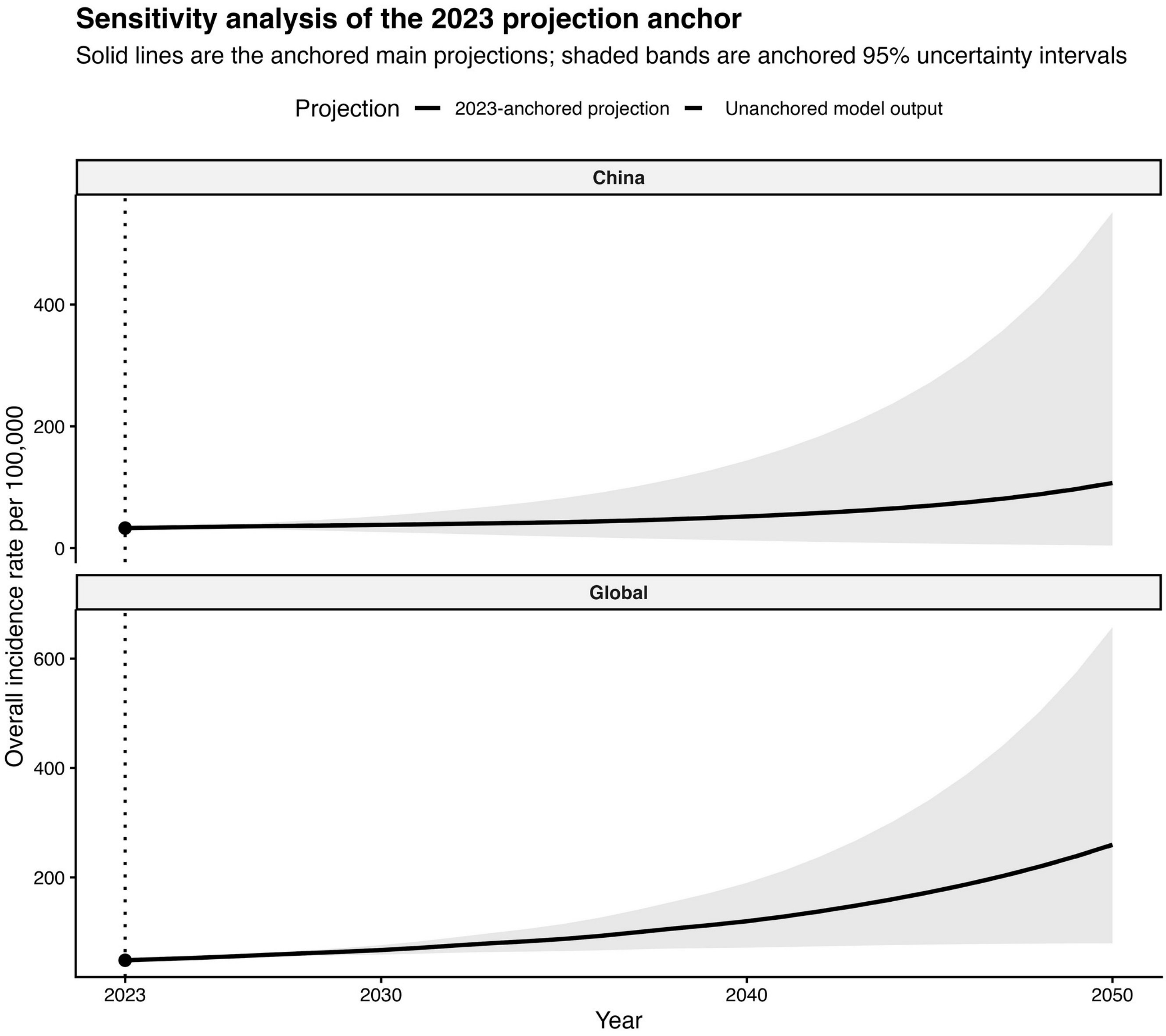

Supplementary Figure S4. Sensitivity of BAPC projections to multiplicative anchoring at the observed 2023 incidence rate.

Supplementary Figure S5.

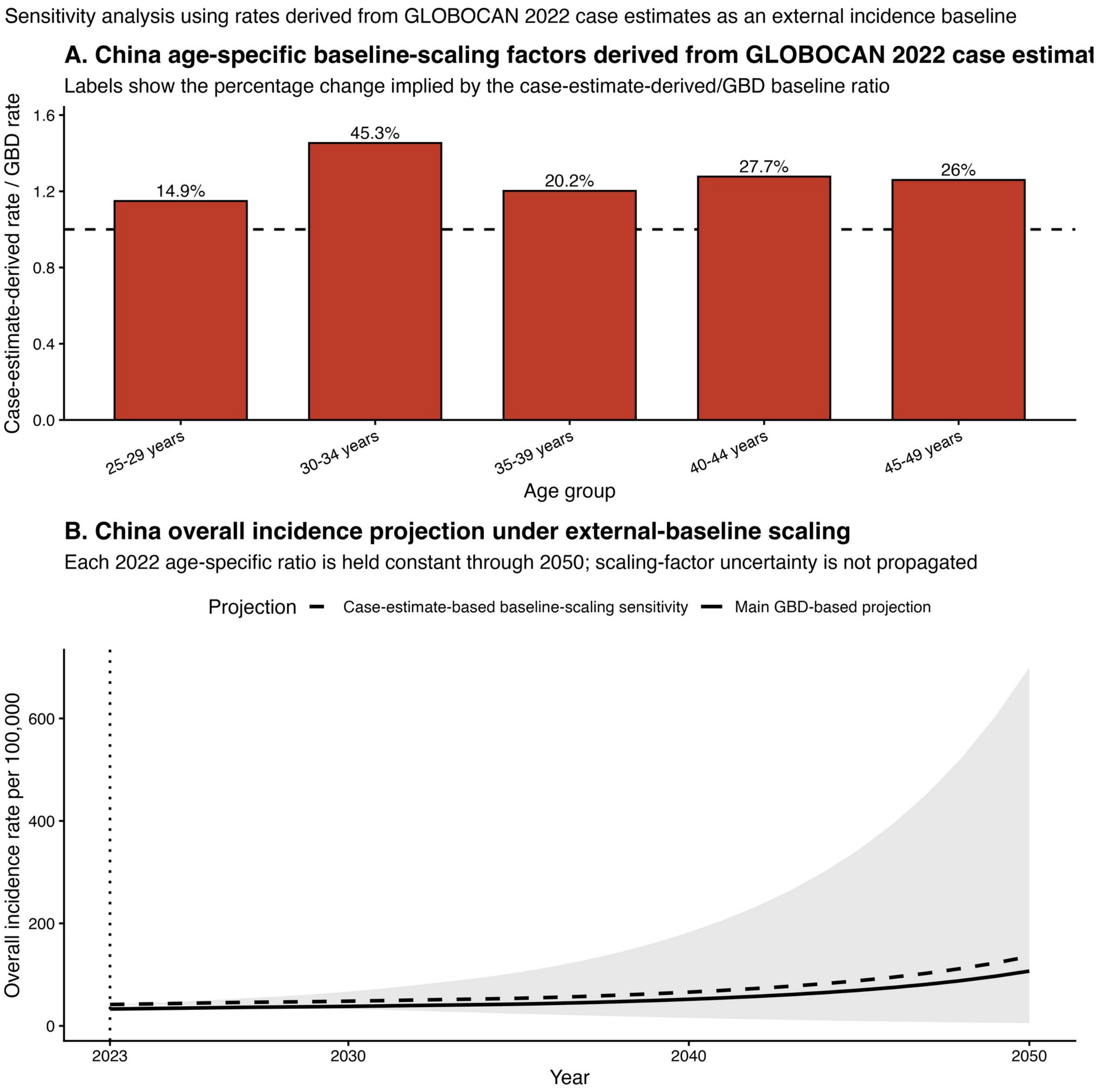

Supplementary Figure S5. Sensitivity analysis using rates derived from GLOBOCAN 2022 case estimates as an external incidence baseline. This scenario preserves the GBD-derived trajectory and is not an independent GLOBOCAN forecast. The scaled intervals retain the relative posterior uncertainty of the main model but do not incorporate uncertainty in the GLOBOCAN-to-GBD baseline-scaling factors.

Supplementary Figure S6.

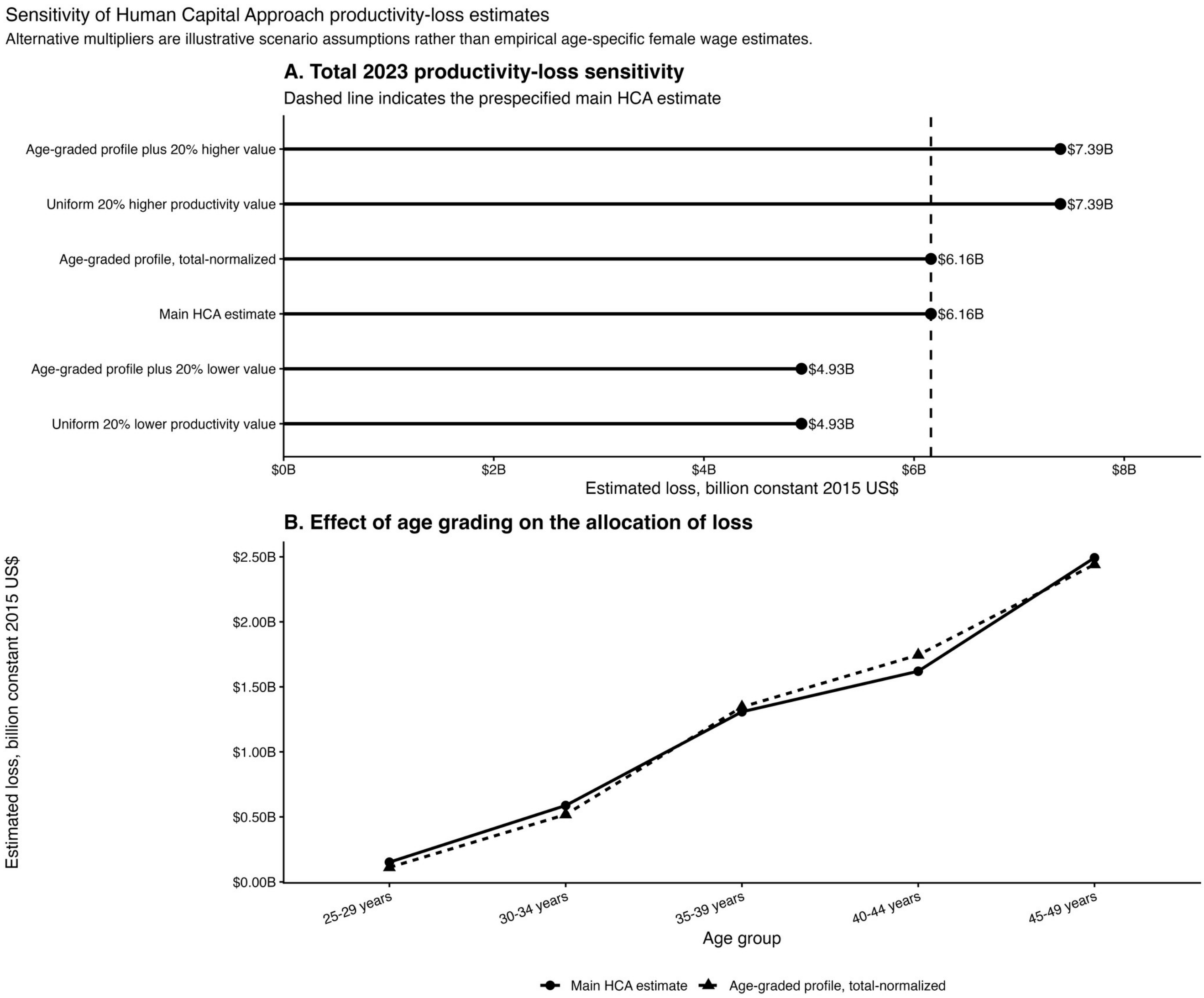

Supplementary Figure S6. Sensitivity of potential mortality-related productivity-loss estimates under the Human Capital Approach to illustrative productivity-value and age-allocation assumptions; the age multipliers are not empirical age-specific female wage estimates. As in the main HCA analysis, total GBD YLLs were not truncated at a prespecified working-age or retirement-age boundary.

Supplementary Tables

Supplementary Table S1. GBD and GLOBOCAN 2022 external comparison

Location	Age group	GLOBOCAN case estimate, 2022	Matched population denominator	Rate derived from GLOBOCAN case estimates, per 100,000	GBD incidence rate, per 100,000	Absolute rate difference	Relative rate difference (%)
China	25-29 years	2,675	38,411,810	6.96	6.06	-0.9	-12.97
China	30-34 years	10,405	55,121,182	18.88	12.99	-5.89	-31.2
China	35-39 years	18,603	54,714,849	34	28.28	-5.72	-16.82
China	40-44 years	29,963	44,090,054	67.96	53.21	-14.74	-21.7
China	45-49 years	49,686	51,683,935	96.13	76.33	-19.81	-20.6
Global	25-29 years	33,777	289,905,907	11.65	9.55	-2.1	-18.06
Global	30-34 years	76,407	296,792,172	25.74	23.89	-1.85	-7.21
Global	35-39 years	128,734	282,893,030	45.51	43.39	-2.11	-4.64
Global	40-44 years	183,276	250,755,571	73.09	74.07	0.98	1.34
Global	45-49 years	239,314	234,247,617	102.16	99.94	-2.23	-2.18

Note: *Rates derived from GLOBOCAN case estimates use matched 2022 population denominators from the GBD-based analytic panel and are not official GLOBOCAN incidence rates. Relative rate difference is expressed as a percentage.*

Supplementary Table S2. Sequential decomposition results

Location	Cases, 1990	Population growth	Age-structure change	Residual epidemiological component	Cases, 2023	Total increase	Population growth (%)	Age structure (%)	Residual (%)
China	50,064	10,568	12,856	13,732	87,221	37,156	28.4%	34.6%	37.0%
Global	278,566	173,667	54,213	165,782	672,228	393,662	44.1%	13.8%	42.1%
High SDI	184,164	35,766	38,968	-8,655	250,244	66,080	54.1%	59.0%	-13.1%
High-middle SDI	40,701	25,975	10,840	36,998	114,514	73,813	35.2%	14.7%	50.1%
Middle SDI	17,215	15,547	5,303	49,430	87,495	70,280	22.1%	7.5%	70.3%
Low-middle SDI	17,211	16,996	1,836	58,234	94,277	77,066	22.1%	2.4%	75.6%
Low SDI	18,960	28,362	1,157	76,642	125,121	106,161	26.7%	1.1%	72.2%

Note: *Incident cases were sequentially decomposed into population growth, age-structure change, and a residual epidemiological component. The residual component is descriptive and should not be interpreted as a directly measured causal or metabolic effect. Percent contributions were calculated from unrounded estimates.*

Supplementary Table S3. PAF and exploratory SHAP ranking in 2023

Risk factor	25-29 years	30-34 years	35-39 years	40-44 years	45-49 years	Descriptive mean of age-specific PAFs	SHAP rank	Mean absolute SHAP
High fasting plasma glucose	0.025	0.032	0.0405	0.0477	0.0556	0.0402	1	85.3
High alcohol use	0.0128	0.0145	0.0146	0.0162	0.0145	0.0145	2	12.1
Low physical activity	0.0125	0.0124	0.012	0.0127	0.0132	0.0126	3	8.8
Tobacco use	0.1266	0.1274	0.127	0.1299	0.1296	0.1281	4	4.3
High BMI	0	0	0	0	0	0	NA	NA

Note: *PAFs are shown as proportions. "Descriptive mean of age-specific PAFs" is the unweighted arithmetic mean across the five age groups and is provided only as a descriptive summary; it should not be interpreted as an overall or population-weighted PAF for women aged 25–49 years. Mean absolute SHAP values are in thousands of DALYs. High BMI was retained descriptively but was not rankable because the selected China-specific series was constant at zero.*

Supplementary Table S4. Mortality-related productivity losses in selected years

Location	Year	Total YLLs, 25-49y	Total productivity loss, billion constant 2015 US$	High BMI loss, billion constant 2015 US$	High fasting plasma glucose loss, billion constant 2015 US$	Tobacco use loss, billion constant 2015 US$	High alcohol use loss, billion constant 2015 US$	Low physical activity loss, billion constant 2015 US$
China	1990	816,139	0.656	0	0.038	0.091	0.01	0.007
China	2000	1,068,018	1.947	0	0.099	0.263	0.024	0.021
China	2010	832,210	3.966	0	0.177	0.525	0.056	0.045
China	2020	589,820	5.138	0	0.233	0.667	0.074	0.065
China	2023	597,996	6.161	0	0.291	0.793	0.092	0.079

Note: *Values represent estimated potential mortality-related productivity loss under the specified Human Capital Approach assumptions and are reported in billion constant 2015 US dollars. Total GBD YLLs were not truncated at a prespecified working-age or retirement-age boundary and may therefore include years beyond conventional working ages. Risk-attributable losses use YLL-specific PAFs. HCA, Human Capital Approach; YLL, year of life lost; PAF, population attributable fraction.*

Supplementary Table S5. BAPC projections in selected years with 95% uncertainty intervals

Location	Age group	Year	Projected incidence rate per 100,000, 95% UI
China	25-29 years	2023	5.99 (5.8 to 6.18)
China	25-29 years	2030	6.76 (4.36 to 9.93)
China	25-29 years	2040	9.79 (1.86 to 30.39)
China	25-29 years	2050	22.94 (0.57 to 129.97)
China	30-34 years	2023	13.4 (13.2 to 13.6)
China	30-34 years	2030	15.25 (10.51 to 21.15)
China	30-34 years	2040	21.35 (4.71 to 61.6)
China	30-34 years	2050	47.02 (1.49 to 254.42)
China	35-39 years	2023	27.24 (26.96 to 27.53)
China	35-39 years	2030	29.98 (20.8 to 41.36)
China	35-39 years	2040	41.41 (9.84 to 114.86)
China	35-39 years	2050	86.95 (3.24 to 454.35)
China	40-44 years	2023	52.14 (51.66 to 52.62)
China	40-44 years	2030	56.55 (39.25 to 78.01)
China	40-44 years	2040	78.78 (19.18 to 215.64)
China	40-44 years	2050	160.01 (6.58 to 817.62)
China	45-49 years	2023	80.54 (79.91 to 81.17)
China	45-49 years	2030	99.58 (69.13 to 137.36)
China	45-49 years	2040	132.28 (32.27 to 361.71)
China	45-49 years	2050	265.01 (11.43 to 1338.86)
China	Overall ASIR (25-49y)	2023	32.87 (32.54 to 33.21)
China	Overall ASIR (25-49y)	2030	38.01 (26.29 to 52.59)
China	Overall ASIR (25-49y)	2040	51.9 (12.37 to 143.82)
China	Overall ASIR (25-49y)	2050	106.87 (4.23 to 551.55)
Global	25-29 years	2023	10.15 (10.04 to 10.26)
Global	25-29 years	2030	16.21 (12.51 to 20.7)
Global	25-29 years	2040	34.21 (13.04 to 74.09)
Global	25-29 years	2050	87.05 (11.03 to 327.14)
Global	30-34 years	2023	25.48 (25.33 to 25.63)
Global	30-34 years	2030	36.95 (32.37 to 41.89)
Global	30-34 years	2040	74.82 (38.09 to 132.78)
Global	30-34 years	2050	175.31 (34.82 to 542.19)
Global	35-39 years	2023	44.92 (44.73 to 45.12)
Global	35-39 years	2030	61.17 (54.02 to 68.85)
Global	35-39 years	2040	119.44 (72.05 to 185.86)
Global	35-39 years	2050	263.16 (73.11 to 683.46)
Global	40-44 years	2023	76.47 (76.19 to 76.75)
Global	40-44 years	2030	103.28 (91.23 to 116.22)
Global	40-44 years	2040	182.4 (116.23 to 271.39)

Location	Age group	Year	Projected incidence rate per 100,000, 95% UI
Global	40-44 years	2050	385.62 (133.35 to 879.78)
Global	45-49 years	2023	103.65 (103.31 to 104)
Global	45-49 years	2030	143.09 (126.4 to 161)
Global	45-49 years	2040	227.16 (145.09 to 337.66)
Global	45-49 years	2050	463.12 (178.75 to 985.14)
Global	Overall ASIR (25-49y)	2023	48.36 (48.15 to 48.56)
Global	Overall ASIR (25-49y)	2030	67.06 (58.75 to 76.1)
Global	Overall ASIR (25-49y)	2040	119.78 (71.48 to 189.8)
Global	Overall ASIR (25-49y)	2050	259.61 (79.32 to 657.4)

Note: *Values are posterior mean incidence rates per 100,000 with 95% posterior uncertainty intervals under the 2023-anchored status-quo BAPC scenario. BAPC, Bayesian age-period-cohort; UI, uncertainty interval.*

Supplementary Table S6. SHAP feature status and importance

Feature	Risk factor	Mean absolute SHAP	SHAP rank	n observed	n unique	Mean	SD	Min	Max	Zero variance	Model status
High_fasting_plasma_glucose	High fasting plasma glucose	85.3	1	170	170	0.0432	0.0131	0.0227	0.0716	No	Included in XGBoost-SHAP
High_alcohol_use	High alcohol use	12.1	2	170	170	0.0131	0.0025	0.0071	0.0174	No	Included in XGBoost-SHAP
Low_physical_activity	Low physical activity	8.8	3	170	170	0.0114	0.0007	0.0105	0.0132	No	Included in XGBoost-SHAP
Tobacco_use	Tobacco use	4.3	4	170	170	0.1333	0.0052	0.1265	0.1455	No	Included in XGBoost-SHAP
High_BMI	High BMI	NA	NA	170	1	0	0	0	0	Yes	Excluded from XGBoost-SHAP: zero variance

Note: *Mean absolute SHAP values quantify model-based feature contributions to predicted DALYs and do not indicate causal effects. The high-BMI feature was excluded from XGBoost-SHAP because its selected China-specific series had zero variance.*

Supplementary Table S7. PAF feature completeness

Feature	n rows	n missing	Missing (%)	n observed	n zero	Zero among observed (%)	n negative	Minimum	Median	Maximum
High_alcohol_use	170	0	0	170	0	0	0	0.0071	0.0137	0.0174
High_BMI	170	0	0	170	170	100	0	0	0	0
High_fasting_plasma_glucose	170	0	0	170	0	0	0	0.0227	0.0439	0.0716
Low_physical_activity	170	0	0	170	0	0	0	0.0105	0.0113	0.0132
Tobacco_use	170	0	0	170	0	0	0	0.1265	0.1314	0.1455

Note: *Completeness was evaluated across 170 year-age observations (34 years × five age groups). No imputation was performed. PAF, population attributable fraction.*

Supplementary Table S8. High-BMI source validation by age

A. Point-estimate validation

Age group	n rows	Point n missing	Point n zero	Point n negative	Point min	Point median	Point max
25-29 years	34	0	34	0	0	0	0
30-34 years	34	0	34	0	0	0	0
35-39 years	34	0	34	0	0	0	0
40-44 years	34	0	34	0	0	0	0
45-49 years	34	0	34	0	0	0	0

B. Lower-bound validation

Age group	Lower n missing	Lower n zero	Lower n negative	Lower min	Lower median	Lower max
25-29 years	0	34	0	0	0	0
30-34 years	0	34	0	0	0	0
35-39 years	0	34	0	0	0	0
40-44 years	0	34	0	0	0	0
45-49 years	0	34	0	0	0	0

C. Upper-bound validation

Age group	Upper n missing	Upper n zero	Upper n negative	Upper min	Upper median	Upper max
25-29 years	0	34	0	0	0	0
30-34 years	0	34	0	0	0	0
35-39 years	0	34	0	0	0	0
40-44 years	0	34	0	0	0	0
45-49 years	0	34	0	0	0	0

Note: *Validation summary for the selected China-specific GBD breast-cancer DALY PAF series for high BMI. Each age group contains 34 annual observations (1990–2023); the point estimate and both uncertainty bounds were zero in all observations.*

Supplementary Table S9. Temporal SHAP bootstrap summary

Feature	Risk factor	Successful resamples	Mean normalized importance	SD normalized importance	Median normalized importance	Q1 normalized importance	Q3 normalized importance	Median rank	Q1 rank	Q3 rank	Top-rank frequency
High_fasting_plasma_glucose	High fasting plasma glucose	200	0.771	0.066	0.777	0.751	0.809	1	1	1	0.99
High_alcohol_use	High alcohol use	200	0.107	0.06	0.1	0.075	0.124	2	2	3	0.01
Low_physical_activity	Low physical activity	200	0.077	0.021	0.077	0.061	0.092	3	2	3	0
Tobacco_use	Tobacco use	200	0.045	0.018	0.042	0.034	0.05	4	4	4	0

Note: *Summary of 200 grouped calendar-block bootstrap resamples. Six non-overlapping 5-year blocks and one final 4-year block were sampled with replacement, retaining all five age groups within selected years. Normalized importance and top-rank frequency are proportions.*

Supplementary Table S10. SHAP structure-sensitivity summary

A. Ranking summary

Scenario	Full-sample top feature	Full-sample top importance	FPG mean importance	FPG median rank	FPG top-rank frequency
Absolute DALYs (primary scale)	High fasting plasma glucose	0.772	0.779	1	1
Age- and smooth-year-adjusted residuals	High alcohol use	0.36	0.33	2	0.36
Age-specific DALY rate	High fasting plasma glucose	0.833	0.853	1	0.995
Within-age annual first differences	High fasting plasma glucose	0.447	0.422	1	0.78
Within-age centered rate and PAFs	High fasting plasma glucose	0.675	0.655	1	0.915

B. Interpretation

Scenario	Interpretation	Suggested interpretation
Absolute DALYs (primary scale)	Primary exploratory formulation; retains population size and between-age differences.	FPG-related PAF remains the leading exploratory feature under this formulation.
Age- and smooth-year-adjusted residuals	Tests residual associations after removing age-group levels and smooth secular trends from both target and features.	The FPG ranking is sensitive to this formulation; the original ranking should be interpreted cautiously.
Age-specific DALY rate	Removes the direct effect of age-group population size but retains between-age rate differences.	FPG-related PAF remains the leading exploratory feature under this formulation.
Within-age annual first differences	Focuses on year-to-year co-movement and removes stable age-level differences.	FPG-related PAF remains prominent but is not uniformly the leading feature.
Within-age centered rate and PAFs	Removes each age group's long-run mean and focuses on within-age temporal deviations.	FPG-related PAF remains the leading exploratory feature under this formulation.

Note: *Sensitivity formulations evaluate how exploratory SHAP rankings change with target scale, population-size effects, between-age structure, annual changes, and residual age/time structure. These results are exploratory and non-causal.*

Supplementary Table S11. Anchored versus unanchored projection

A. Anchored projections

Location	Age group	Year	Observed rate	Anchored rate	Anchored lower 95%	Anchored upper 95%	Anchored 95% UI
China	25-29 years	2023	5.99	5.99	5.8	6.18	5.99 (5.8 to 6.18)
China	25-29 years	2030	NA	6.76	4.36	9.93	6.76 (4.36 to 9.93)
China	25-29 years	2040	NA	9.79	1.86	30.39	9.79 (1.86 to 30.39)
China	25-29 years	2050	NA	22.94	0.57	129.97	22.94 (0.57 to 129.97)
China	30-34 years	2023	13.4	13.4	13.2	13.6	13.4 (13.2 to 13.6)
China	30-34 years	2030	NA	15.25	10.51	21.15	15.25 (10.51 to 21.15)
China	30-34 years	2040	NA	21.35	4.71	61.6	21.35 (4.71 to 61.6)
China	30-34 years	2050	NA	47.02	1.49	254.42	47.02 (1.49 to 254.42)
China	35-39 years	2023	27.24	27.24	26.96	27.53	27.24 (26.96 to 27.53)
China	35-39 years	2030	NA	29.98	20.8	41.36	29.98 (20.8 to 41.36)
China	35-39 years	2040	NA	41.41	9.84	114.86	41.41 (9.84 to 114.86)
China	35-39 years	2050	NA	86.95	3.24	454.35	86.95 (3.24 to 454.35)
China	40-44 years	2023	52.14	52.14	51.66	52.62	52.14 (51.66 to 52.62)
China	40-44 years	2030	NA	56.55	39.25	78.01	56.55 (39.25 to 78.01)
China	40-44 years	2040	NA	78.78	19.18	215.64	78.78 (19.18 to 215.64)
China	40-44 years	2050	NA	160.01	6.58	817.62	160.01 (6.58 to 817.62)
China	45-49 years	2023	80.54	80.54	79.91	81.17	80.54 (79.91 to 81.17)
China	45-49 years	2030	NA	99.58	69.13	137.36	99.58 (69.13 to 137.36)
China	45-49 years	2040	NA	132.28	32.27	361.71	132.28 (32.27 to 361.71)
China	45-49 years	2050	NA	265.01	11.43	1338.86	265.01 (11.43 to 1338.86)
China	Overall ASIR (25-49y)	2023	32.87	32.87	32.54	33.21	32.87 (32.54 to 33.21)
China	Overall ASIR (25-49y)	2030	NA	38.01	26.29	52.59	38.01 (26.29 to 52.59)
China	Overall ASIR (25-49y)	2040	NA	51.9	12.37	143.82	51.9 (12.37 to 143.82)
China	Overall ASIR (25-49y)	2050	NA	106.87	4.23	551.55	106.87 (4.23 to 551.55)
Global	25-29 years	2023	10.15	10.15	10.04	10.26	10.15 (10.04 to 10.26)
Global	25-29 years	2030	NA	16.21	12.51	20.7	16.21 (12.51 to 20.7)
Global	25-29 years	2040	NA	34.21	13.04	74.09	34.21 (13.04 to 74.09)
Global	25-29 years	2050	NA	87.05	11.03	327.14	87.05 (11.03 to 327.14)
Global	30-34 years	2023	25.48	25.48	25.33	25.63	25.48 (25.33 to 25.63)
Global	30-34 years	2030	NA	36.95	32.37	41.89	36.95 (32.37 to 41.89)
Global	30-34 years	2040	NA	74.82	38.09	132.78	74.82 (38.09 to 132.78)
Global	30-34 years	2050	NA	175.31	34.82	542.19	175.31 (34.82 to 542.19)
Global	35-39 years	2023	44.92	44.92	44.73	45.12	44.92 (44.73 to 45.12)
Global	35-39 years	2030	NA	61.17	54.02	68.85	61.17 (54.02 to 68.85)
Global	35-39 years	2040	NA	119.44	72.05	185.86	119.44 (72.05 to 185.86)
Global	35-39 years	2050	NA	263.16	73.11	683.46	263.16 (73.11 to 683.46)
Global	40-44 years	2023	76.47	76.47	76.19	76.75	76.47 (76.19 to 76.75)
Global	40-44 years	2030	NA	103.28	91.23	116.22	103.28 (91.23 to 116.22)
Global	40-44 years	2040	NA	182.4	116.23	271.39	182.4 (116.23 to 271.39)
Global	40-44 years	2050	NA	385.62	133.35	879.78	385.62 (133.35 to 879.78)

Location	Age group	Year	Observed rate	Anchored rate	Anchored lower 95%	Anchored upper 95%	Anchored 95% UI
Global	45-49 years	2023	103.65	103.65	103.31	104	103.65 (103.31 to 104)
Global	45-49 years	2030	NA	143.09	126.4	161	143.09 (126.4 to 161)
Global	45-49 years	2040	NA	227.16	145.09	337.66	227.16 (145.09 to 337.66)
Global	45-49 years	2050	NA	463.12	178.75	985.14	463.12 (178.75 to 985.14)
Global	Overall ASIR (25-49y)	2023	48.36	48.36	48.15	48.56	48.36 (48.15 to 48.56)
Global	Overall ASIR (25-49y)	2030	NA	67.06	58.75	76.1	67.06 (58.75 to 76.1)
Global	Overall ASIR (25-49y)	2040	NA	119.78	71.48	189.8	119.78 (71.48 to 189.8)
Global	Overall ASIR (25-49y)	2050	NA	259.61	79.32	657.4	259.61 (79.32 to 657.4)

B. Unanchored comparison and change

Location	Age group	Year	Unanchored rate	Unanchored lower 95%	Unanchored upper 95%	Unanchored 95% UI	Absolute change	Relative change (%)
China	25-29 years	2023	6.07	5.88	6.26	6.07 (5.88 to 6.26)	-0.08	-1.35
China	25-29 years	2030	6.85	4.42	10.07	6.85 (4.42 to 10.07)	-0.09	-1.35
China	25-29 years	2040	9.92	1.88	30.81	9.92 (1.88 to 30.81)	-0.13	-1.35
China	25-29 years	2050	23.26	0.57	131.75	23.26 (0.57 to 131.75)	-0.31	-1.35
China	30-34 years	2023	13.17	12.98	13.36	13.17 (12.98 to 13.36)	0.23	1.74
China	30-34 years	2030	14.99	10.33	20.79	14.99 (10.33 to 20.79)	0.26	1.74
China	30-34 years	2040	20.99	4.63	60.55	20.99 (4.63 to 60.55)	0.36	1.74
China	30-34 years	2050	46.22	1.46	250.08	46.22 (1.46 to 250.08)	0.8	1.74
China	35-39 years	2023	26.63	26.35	26.91	26.63 (26.35 to 26.91)	0.61	2.31
China	35-39 years	2030	29.31	20.33	40.43	29.31 (20.33 to 40.43)	0.68	2.31
China	35-39 years	2040	40.48	9.62	112.28	40.48 (9.62 to 112.28)	0.93	2.31
China	35-39 years	2050	84.99	3.17	444.11	84.99 (3.17 to 444.11)	1.96	2.31
China	40-44 years	2023	52.08	51.6	52.56	52.08 (51.6 to 52.56)	0.06	0.12
China	40-44 years	2030	56.48	39.21	77.91	56.48 (39.21 to 77.91)	0.07	0.12
China	40-44 years	2040	78.68	19.16	215.38	78.68 (19.16 to 215.38)	0.09	0.12
China	40-44 years	2050	159.82	6.57	816.63	159.82 (6.57 to 816.63)	0.19	0.12
China	45-49 years	2023	81.92	81.28	82.56	81.92 (81.28 to 82.56)	-1.38	-1.69
China	45-49 years	2030	101.29	70.31	139.72	101.29 (70.31 to 139.72)	-1.71	-1.69
China	45-49 years	2040	134.55	32.82	367.91	134.55 (32.82 to 367.91)	-2.27	-1.69
China	45-49 years	2050	269.56	11.63	1361.83	269.56 (11.63 to 1361.83)	-4.55	-1.69
China	Overall ASIR (25-49y)	2023	32.94	32.6	33.28	32.94 (32.6 to 33.28)	-0.07	-0.2
China	Overall ASIR (25-49y)	2030	38.11	26.36	52.74	38.11 (26.36 to 52.74)	-0.1	-0.27
China	Overall ASIR (25-49y)	2040	52.03	12.4	144.17	52.03 (12.4 to 144.17)	-0.13	-0.25
China	Overall ASIR (25-49y)	2050	107.11	4.25	552.64	107.11 (4.25 to 552.64)	-0.23	-0.22
Global	25-29 years	2023	10.14	10.04	10.25	10.14 (10.04 to 10.25)	0.01	0.06
Global	25-29 years	2030	16.2	12.5	20.69	16.2 (12.5 to 20.69)	0.01	0.06
Global	25-29 years	2040	34.19	13.03	74.05	34.19 (13.03 to 74.05)	0.02	0.06
Global	25-29 years	2050	87	11.02	326.95	87 (11.02 to 326.95)	0.05	0.06
Global	30-34 years	2023	24.87	24.72	25.02	24.87 (24.72 to 25.02)	0.61	2.46
Global	30-34 years	2030	36.07	31.59	40.89	36.07 (31.59 to 40.89)	0.89	2.46
Global	30-34 years	2040	73.03	37.17	129.6	73.03 (37.17 to 129.6)	1.79	2.46

Location	Age group	Year	Unanchored rate	Unanchored lower 95%	Unanchored upper 95%	Unanchored 95% UI	Absolute change	Relative change (%)
Global	30-34 years	2050	171.11	33.99	529.19	171.11 (33.99 to 529.19)	4.2	2.46
Global	35-39 years	2023	44.34	44.14	44.53	44.34 (44.14 to 44.53)	0.59	1.32
Global	35-39 years	2030	60.37	53.32	67.96	60.37 (53.32 to 67.96)	0.8	1.32
Global	35-39 years	2040	117.88	71.11	183.43	117.88 (71.11 to 183.43)	1.56	1.32
Global	35-39 years	2050	259.72	72.16	674.54	259.72 (72.16 to 674.54)	3.43	1.32
Global	40-44 years	2023	76.61	76.33	76.89	76.61 (76.33 to 76.89)	-0.14	-0.19
Global	40-44 years	2030	103.48	91.4	116.44	103.48 (91.4 to 116.44)	-0.19	-0.19
Global	40-44 years	2040	182.74	116.44	271.9	182.74 (116.44 to 271.9)	-0.34	-0.19
Global	40-44 years	2050	386.34	133.6	881.41	386.34 (133.6 to 881.41)	-0.72	-0.19
Global	45-49 years	2023	105.12	104.76	105.47	105.12 (104.76 to 105.47)	-1.46	-1.39
Global	45-49 years	2030	145.11	128.18	163.27	145.11 (128.18 to 163.27)	-2.02	-1.39
Global	45-49 years	2040	230.36	147.13	342.41	230.36 (147.13 to 342.41)	-3.2	-1.39
Global	45-49 years	2050	469.65	181.27	999.02	469.65 (181.27 to 999.02)	-6.52	-1.39
Global	Overall ASIR (25-49y)	2023	48.38	48.17	48.58	48.38 (48.17 to 48.58)	-0.02	-0.04
Global	Overall ASIR (25-49y)	2030	67.08	58.77	76.12	67.08 (58.77 to 76.12)	-0.02	-0.03
Global	Overall ASIR (25-49y)	2040	119.68	71.47	189.51	119.68 (71.47 to 189.51)	0.11	0.09
Global	Overall ASIR (25-49y)	2050	259.23	79.42	655.39	259.23 (79.42 to 655.39)	0.38	0.15

Note: *Anchored projections were multiplicatively aligned to the observed 2023 age-specific incidence boundary. Relative changes compare anchored with unanchored point estimates. UI, uncertainty interval.*

Supplementary Table S12. GLOBOCAN-based baseline-scaled projection sensitivity

Location	Age group	Year	Baseline-scaling factor	Main GBD-based projection per 100,000 (95% UI)	Case-estimate-based baseline-scaled sensitivity per 100,000 (95% UI)	Relative change after baseline scaling (%)
China	25-29 years	2023	1.149	5.99 (5.8 to 6.18)	6.88 (6.66 to 7.1)	14.9
China	25-29 years	2030	1.149	6.76 (4.36 to 9.93)	7.77 (5.01 to 11.41)	14.9
China	25-29 years	2040	1.149	9.79 (1.86 to 30.39)	11.24 (2.14 to 34.92)	14.9
China	25-29 years	2050	1.149	22.94 (0.57 to 129.97)	26.36 (0.65 to 149.34)	14.9
China	30-34 years	2023	1.453	13.4 (13.2 to 13.6)	19.47 (19.19 to 19.76)	45.34
China	30-34 years	2030	1.453	15.25 (10.51 to 21.15)	22.16 (15.28 to 30.75)	45.34
China	30-34 years	2040	1.453	21.35 (4.71 to 61.6)	31.03 (6.84 to 89.53)	45.34
China	30-34 years	2050	1.453	47.02 (1.49 to 254.42)	68.34 (2.16 to 369.78)	45.34
China	35-39 years	2023	1.202	27.24 (26.96 to 27.53)	32.75 (32.41 to 33.1)	20.22
China	35-39 years	2030	1.202	29.98 (20.8 to 41.36)	36.05 (25.01 to 49.73)	20.22
China	35-39 years	2040	1.202	41.41 (9.84 to 114.86)	49.79 (11.83 to 138.09)	20.22
China	35-39 years	2050	1.202	86.95 (3.24 to 454.35)	104.54 (3.9 to 546.23)	20.22
China	40-44 years	2023	1.277	52.14 (51.66 to 52.62)	66.59 (65.98 to 67.2)	27.71
China	40-44 years	2030	1.277	56.55 (39.25 to 78.01)	72.22 (50.13 to 99.62)	27.71
China	40-44 years	2040	1.277	78.78 (19.18 to 215.64)	100.61 (24.49 to 275.38)	27.71
China	40-44 years	2050	1.277	160.01 (6.58 to 817.62)	204.35 (8.4 to 1044.15)	27.71
China	45-49 years	2023	1.26	80.54 (79.91 to 81.17)	101.44 (100.65 to 102.23)	25.95
China	45-49 years	2030	1.26	99.58 (69.13 to 137.36)	125.43 (87.07 to 173.01)	25.95
China	45-49 years	2040	1.26	132.28 (32.27 to 361.71)	166.61 (40.64 to 455.57)	25.95
China	45-49 years	2050	1.26	265.01 (11.43 to 1338.86)	333.79 (14.4 to 1686.32)	25.95
China	Overall ASIR (25-49y)	2023	1.268	32.87 (32.54 to 33.21)	41.67 (41.24 to 42.1)	26.76
China	Overall ASIR (25-49y)	2030	1.268	38.01 (26.29 to 52.59)	48.17 (33.33 to 66.65)	26.76
China	Overall ASIR (25-49y)	2040	1.268	51.9 (12.37 to 143.82)	65.8 (15.68 to 182.34)	26.77
China	Overall ASIR (25-49y)	2050	1.268	106.87 (4.23 to 551.55)	135.51 (5.37 to 699.46)	26.8
Global	25-29 years	2023	1.22	10.15 (10.04 to 10.26)	12.39 (12.26 to 12.52)	22.03
Global	25-29 years	2030	1.22	16.21 (12.51 to 20.7)	19.78 (15.26 to 25.26)	22.03
Global	25-29 years	2040	1.22	34.21 (13.04 to 74.09)	41.75 (15.91 to 90.42)	22.03
Global	25-29 years	2050	1.22	87.05 (11.03 to 327.14)	106.23 (13.46 to 399.23)	22.03
Global	30-34 years	2023	1.078	25.48 (25.33 to 25.63)	27.46 (27.29 to 27.62)	7.76
Global	30-34 years	2030	1.078	36.95 (32.37 to 41.89)	39.82 (34.88 to 45.15)	7.76
Global	30-34 years	2040	1.078	74.82 (38.09 to 132.78)	80.63 (41.04 to 143.09)	7.76
Global	30-34 years	2050	1.078	175.31 (34.82 to 542.19)	188.92 (37.53 to 584.29)	7.76
Global	35-39 years	2023	1.049	44.92 (44.73 to 45.12)	47.11 (46.91 to 47.32)	4.87
Global	35-39 years	2030	1.049	61.17 (54.02 to 68.85)	64.15 (56.65 to 72.21)	4.87
Global	35-39 years	2040	1.049	119.44 (72.05 to 185.86)	125.25 (75.56 to 194.91)	4.87
Global	35-39 years	2050	1.049	263.16 (73.11 to 683.46)	275.97 (76.67 to 716.74)	4.87
Global	40-44 years	2023	0.987	76.47 (76.19 to 76.75)	75.46 (75.19 to 75.74)	-1.32
Global	40-44 years	2030	0.987	103.28 (91.23 to 116.22)	101.92 (90.03 to 114.69)	-1.32
Global	40-44 years	2040	0.987	182.4 (116.23 to 271.39)	179.99 (114.7 to 267.81)	-1.32

Location	Age group	Year	Baseline-scaling factor	Main GBD-based projection per 100,000 (95% UI)	Case-estimate-based baseline-scaled sensitivity per 100,000 (95% UI)	Relative change after baseline scaling (%)
Global	40-44 years	2050	0.987	385.62 (133.35 to 879.78)	380.53 (131.6 to 868.17)	-1.32
Global	45-49 years	2023	1.022	103.65 (103.31 to 104)	105.96 (105.61 to 106.32)	2.23
Global	45-49 years	2030	1.022	143.09 (126.4 to 161)	146.28 (129.22 to 164.59)	2.23
Global	45-49 years	2040	1.022	227.16 (145.09 to 337.66)	232.22 (148.32 to 345.18)	2.23
Global	45-49 years	2050	1.022	463.12 (178.75 to 985.14)	473.44 (182.73 to 1007.09)	2.23
Global	Overall ASIR (25-49y)	2023	1.033	48.36 (48.15 to 48.56)	49.94 (49.72 to 50.16)	3.27
Global	Overall ASIR (25-49y)	2030	1.035	67.06 (58.75 to 76.1)	69.38 (60.71 to 78.84)	3.47
Global	Overall ASIR (25-49y)	2040	1.038	119.78 (71.48 to 189.8)	124.35 (73.74 to 198.22)	3.81
Global	Overall ASIR (25-49y)	2050	1.041	259.61 (79.32 to 657.4)	270.34 (81.53 to 691.36)	4.13

Note: *The sensitivity analysis applies age-specific case-estimate-derived-to-GBD baseline-scaling factors to the main BAPC projections. Scaling-factor uncertainty is not propagated into the reported scaled uncertainty intervals.*

Supplementary Table S13. HCA sensitivity scenarios in 2023

Year	Scenario	Total loss (billion)	Main loss (billion)	Absolute change (billion)	Relative change (%)	Interpretation
2023	Uniform 20% lower productivity value	4.929	6.161	-1.232	-20	Lower-bound scenario in which the productivity-value parameter applied to YLLs is 20% below GDP per capita.
2023	Age-graded profile plus 20% lower value	4.929	6.161	-1.232	-20	Combines age grading with a 20% lower monetary value.
2023	Main HCA estimate	6.161	6.161	0	0	Prespecified main estimate using GDP per capita and age-specific female labour-force participation.
2023	Age-graded profile, total-normalized	6.161	6.161	0	0	Redistributes the 2023 loss across age groups while preserving the overall 2023 monetary scale.
2023	Uniform 20% higher productivity value	7.393	6.161	1.232	20	Upper-bound scenario in which the productivity-value parameter applied to YLLs is 20% above GDP per capita.
2023	Age-graded profile plus 20% higher value	7.393	6.161	1.232	20	Combines age grading with a 20% higher monetary value.

Note: *HCA sensitivity scenarios use illustrative productivity-value and age-allocation assumptions. Age multipliers are not empirical age-specific female wage estimates. These scenarios do not truncate total GBD YLLs at a prespecified working-age or retirement-age boundary and should be interpreted as sensitivity analyses of potential mortality-related productivity loss under the stated HCA assumptions. Monetary values are in billion constant 2015 US dollars.*

Supplementary Table S14. Burden counts, rates, and 95% uncertainty intervals

Location	Year	Measure	Metric	Estimate	Lower 95% UI	Upper 95% UI	Formatted 95% UI
China	1990	Incidence	Number	50,064	37,131	64,200	50064 (37131 to 64200)
China	1990	Incidence	Weighted rate per 100,000	28.06	20.84	35.86	28.06 (20.84 to 35.86)
China	1990	Deaths	Number	16,414	12,412	20,953	16414 (12412 to 20953)
China	1990	Deaths	Weighted rate per 100,000	9.25	7	11.78	9.25 (7 to 11.78)
China	1990	YLLs	Number	816,139	616,204	1,043,382	816139 (616204 to 1043382)
China	1990	YLLs	Weighted rate per 100,000	451.5	341.53	576	451.5 (341.53 to 576)
China	1990	YLDs	Number	33,626	21,684	50,127	33626 (21684 to 50127)
China	1990	YLDs	Weighted rate per 100,000	18.78	12.14	27.95	18.78 (12.14 to 27.95)
China	1990	DALYs	Number	849,765	645,381	1,079,435	849765 (645381 to 1079435)
China	1990	DALYs	Weighted rate per 100,000	470.29	357.83	596.25	470.29 (357.83 to 596.25)
China	2023	Incidence	Number	87,221	61,492	122,482	87221 (61492 to 122482)
China	2023	Incidence	Weighted rate per 100,000	32.87	23.19	46.18	32.87 (23.19 to 46.18)
China	2023	Deaths	Number	12,611	9,232	16,869	12611 (9232 to 16869)
China	2023	Deaths	Weighted rate per 100,000	4.72	3.45	6.33	4.72 (3.45 to 6.33)
China	2023	YLLs	Number	597,996	436,745	801,542	597996 (436745 to 801542)
China	2023	YLLs	Weighted rate per 100,000	225.86	164.71	303.53	225.86 (164.71 to 303.53)
China	2023	YLDs	Number	60,262	38,889	93,464	60262 (38889 to 93464)
China	2023	YLDs	Weighted rate per 100,000	22.75	14.66	35.24	22.75 (14.66 to 35.24)
China	2023	DALYs	Number	658,257	487,978	879,835	658257 (487978 to 879835)
China	2023	DALYs	Weighted rate per 100,000	248.61	184.07	332.76	248.61 (184.07 to 332.76)
Global	1990	Incidence	Number	278,566	244,818	318,547	278566 (244818 to 318547)
Global	1990	Incidence	Weighted rate per 100,000	36.39	32.02	41.56	36.39 (32.02 to 41.56)
Global	1990	Deaths	Number	84,800	75,013	94,443	84800 (75013 to 94443)
Global	1990	Deaths	Weighted rate per 100,000	11.07	9.8	12.3	11.07 (9.8 to 12.3)
Global	1990	YLLs	Number	4,145,105	3,659,271	4,628,906	4145105 (3659271 to 4628906)
Global	1990	YLLs	Weighted rate per 100,000	534.33	472.48	595.34	534.33 (472.48 to 595.34)
Global	1990	YLDs	Number	188,344	129,431	262,688	188344 (129431 to 262688)
Global	1990	YLDs	Weighted rate per 100,000	24.55	16.88	34.25	24.55 (16.88 to 34.25)
Global	1990	DALYs	Number	4,333,449	3,820,042	4,850,403	4333449 (3820042 to 4850403)
Global	1990	DALYs	Weighted rate per 100,000	558.88	493.56	624.2	558.88 (493.56 to 624.2)
Global	2023	Incidence	Number	672,228	560,147	806,448	672228 (560147 to 806448)
Global	2023	Incidence	Weighted rate per 100,000	48.36	40.28	58.02	48.36 (40.28 to 58.02)
Global	2023	Deaths	Number	179,756	147,225	215,234	179756 (147225 to 215234)
Global	2023	Deaths	Weighted rate per 100,000	12.92	10.58	15.48	12.92 (10.58 to 15.48)
Global	2023	YLLs	Number	8,793,441	7,176,938	10,563,347	8793441 (7176938 to 10563347)
Global	2023	YLLs	Weighted rate per 100,000	632.9	516.41	760.61	632.9 (516.41 to 760.61)
Global	2023	YLDs	Number	435,528	297,800	623,726	435528 (297800 to 623726)
Global	2023	YLDs	Weighted rate per 100,000	31.34	21.43	44.9	31.34 (21.43 to 44.9)
Global	2023	DALYs	Number	9,228,969	7,593,015	11,116,114	9228969 (7593015 to 11116114)

Location	Year	Measure	Metric	Estimate	Lower 95% UI	Upper 95% UI	Formatted 95% UI
Global	2023	DALYs	Weighted rate per 100,000	664.24	546.31	800.45	664.24 (546.31 to 800.45)
High SDI	1990	Incidence	Number	184,164	164,593	204,903	184164 (164593 to 204903)
High SDI	1990	Incidence	Weighted rate per 100,000	53.65	48	59.67	53.65 (48 to 59.67)
High SDI	1990	Deaths	Number	42,525	40,085	45,014	42525 (40085 to 45014)
High SDI	1990	Deaths	Weighted rate per 100,000	12.4	11.7	13.12	12.4 (11.7 to 13.12)
High SDI	1990	YLLs	Number	2,048,923	1,928,278	2,172,663	2048923 (1928278 to 2172663)
High SDI	1990	YLLs	Weighted rate per 100,000	592.45	558.08	627.55	592.45 (558.08 to 627.55)
High SDI	1990	YLDs	Number	127,947	88,082	178,128	127947 (88082 to 178128)
High SDI	1990	YLDs	Weighted rate per 100,000	37.21	25.61	51.83	37.21 (25.61 to 51.83)
High SDI	1990	DALYs	Number	2,176,870	2,038,681	2,311,858	2176870 (2038681 to 2311858)
High SDI	1990	DALYs	Weighted rate per 100,000	629.66	590.2	668.03	629.66 (590.2 to 668.03)
High SDI	2023	Incidence	Number	250,244	215,636	287,282	250244 (215636 to 287282)
High SDI	2023	Incidence	Weighted rate per 100,000	52.08	44.85	59.81	52.08 (44.85 to 59.81)
High SDI	2023	Deaths	Number	36,344	33,514	39,444	36344 (33514 to 39444)
High SDI	2023	Deaths	Weighted rate per 100,000	7.53	6.94	8.18	7.53 (6.94 to 8.18)
High SDI	2023	YLLs	Number	1,726,448	1,591,311	1,874,715	1726448 (1591311 to 1874715)
High SDI	2023	YLLs	Weighted rate per 100,000	360.83	332.47	392.06	360.83 (332.47 to 392.06)
High SDI	2023	YLDs	Number	172,982	121,085	247,813	172982 (121085 to 247813)
High SDI	2023	YLDs	Weighted rate per 100,000	36.06	25.24	51.65	36.06 (25.24 to 51.65)
High SDI	2023	DALYs	Number	1,899,431	1,743,556	2,072,769	1899431 (1743556 to 2072769)
High SDI	2023	DALYs	Weighted rate per 100,000	396.89	364.24	433.35	396.89 (364.24 to 433.35)
High-middle SDI	1990	Incidence	Number	40,701	32,426	48,502	40701 (32426 to 48502)
High-middle SDI	1990	Incidence	Weighted rate per 100,000	25.23	20.17	29.98	25.23 (20.17 to 29.98)
High-middle SDI	1990	Deaths	Number	15,466	12,490	18,015	15466 (12490 to 18015)
High-middle SDI	1990	Deaths	Weighted rate per 100,000	9.65	7.82	11.23	9.65 (7.82 to 11.23)
High-middle SDI	1990	YLLs	Number	762,882	614,362	889,558	762882 (614362 to 889558)
High-middle SDI	1990	YLLs	Weighted rate per 100,000	468.25	378.34	545.31	468.25 (378.34 to 545.31)
High-middle SDI	1990	YLDs	Number	26,273	17,831	37,496	26273 (17831 to 37496)
High-middle SDI	1990	YLDs	Weighted rate per 100,000	16.22	11.05	23.11	16.22 (11.05 to 23.11)
High-middle SDI	1990	DALYs	Number	789,155	635,807	919,302	789155 (635807 to 919302)
High-middle SDI	1990	DALYs	Weighted rate per 100,000	484.47	391.55	563.65	484.47 (391.55 to 563.65)
High-middle SDI	2023	Incidence	Number	114,514	93,655	139,080	114514 (93655 to 139080)
High-middle SDI	2023	Incidence	Weighted rate per 100,000	37.25	30.44	45.24	37.25 (30.44 to 45.24)
High-middle SDI	2023	Deaths	Number	27,810	23,091	33,040	27810 (23091 to 33040)
High-middle SDI	2023	Deaths	Weighted rate per 100,000	9.02	7.49	10.73	9.02 (7.49 to 10.73)
High-middle SDI	2023	YLLs	Number	1,341,898	1,110,791	1,597,678	1341898 (1110791 to 1597678)
High-middle SDI	2023	YLLs	Weighted rate per 100,000	436.93	361.47	520.61	436.93 (361.47 to 520.61)
High-middle SDI	2023	YLDs	Number	73,114	50,175	104,951	73114 (50175 to 104951)
High-middle SDI	2023	YLDs	Weighted rate per 100,000	23.8	16.33	34.17	23.8 (16.33 to 34.17)
High-middle SDI	2023	DALYs	Number	1,415,012	1,176,569	1,673,797	1415012 (1176569 to 1673797)
High-middle SDI	2023	DALYs	Weighted rate per 100,000	460.73	382.91	545.39	460.73 (382.91 to 545.39)

Location	Year	Measure	Metric	Estimate	Lower 95% UI	Upper 95% UI	Formatted 95% UI
Middle SDI	1990	Incidence	Number	17,215	13,896	21,317	17215 (13896 to 21317)
Middle SDI	1990	Incidence	Weighted rate per 100,000	21.96	17.71	27.19	21.96 (17.71 to 27.19)
Middle SDI	1990	Deaths	Number	7,565	6,143	9,324	7565 (6143 to 9324)
Middle SDI	1990	Deaths	Weighted rate per 100,000	9.73	7.89	12	9.73 (7.89 to 12)
Middle SDI	1990	YLLs	Number	369,552	300,444	455,571	369552 (300444 to 455571)
Middle SDI	1990	YLLs	Weighted rate per 100,000	468.76	380.57	577.99	468.76 (380.57 to 577.99)
Middle SDI	1990	YLDs	Number	11,004	7,229	15,465	11004 (7229 to 15465)
Middle SDI	1990	YLDs	Weighted rate per 100,000	13.97	9.16	19.59	13.97 (9.16 to 19.59)
Middle SDI	1990	DALYs	Number	380,556	309,181	469,131	380556 (309181 to 469131)
Middle SDI	1990	DALYs	Weighted rate per 100,000	482.73	391.63	595.1	482.73 (391.63 to 595.1)
Middle SDI	2023	Incidence	Number	87,495	68,965	110,045	87495 (68965 to 110045)
Middle SDI	2023	Incidence	Weighted rate per 100,000	50.49	39.79	63.51	50.49 (39.79 to 63.51)
Middle SDI	2023	Deaths	Number	26,739	21,078	33,211	26739 (21078 to 33211)
Middle SDI	2023	Deaths	Weighted rate per 100,000	15.41	12.15	19.14	15.41 (12.15 to 19.14)
Middle SDI	2023	YLLs	Number	1,290,280	1,016,694	1,602,758	1290280 (1016694 to 1602758)
Middle SDI	2023	YLLs	Weighted rate per 100,000	744.34	586.42	924.69	744.34 (586.42 to 924.69)
Middle SDI	2023	YLDs	Number	55,970	37,367	83,999	55970 (37367 to 83999)
Middle SDI	2023	YLDs	Weighted rate per 100,000	32.31	21.57	48.5	32.31 (21.57 to 48.5)
Middle SDI	2023	DALYs	Number	1,346,250	1,061,559	1,668,346	1346250 (1061559 to 1668346)
Middle SDI	2023	DALYs	Weighted rate per 100,000	776.65	612.33	962.56	776.65 (612.33 to 962.56)
Low-middle SDI	1990	Incidence	Number	17,211	13,217	22,478	17211 (13217 to 22478)
Low-middle SDI	1990	Incidence	Weighted rate per 100,000	18.38	14.13	23.98	18.38 (14.13 to 23.98)
Low-middle SDI	1990	Deaths	Number	8,760	6,739	11,281	8760 (6739 to 11281)
Low-middle SDI	1990	Deaths	Weighted rate per 100,000	9.42	7.25	12.12	9.42 (7.25 to 12.12)
Low-middle SDI	1990	YLLs	Number	436,008	335,369	562,405	436008 (335369 to 562405)
Low-middle SDI	1990	YLLs	Weighted rate per 100,000	462.55	355.93	595.61	462.55 (355.93 to 595.61)
Low-middle SDI	1990	YLDs	Number	10,701	6,767	15,654	10701 (6767 to 15654)
Low-middle SDI	1990	YLDs	Weighted rate per 100,000	11.4	7.21	16.63	11.4 (7.21 to 16.63)
Low-middle SDI	1990	DALYs	Number	446,708	343,564	576,212	446708 (343564 to 576212)
Low-middle SDI	1990	DALYs	Weighted rate per 100,000	473.95	364.69	610.36	473.95 (364.69 to 610.36)
Low-middle SDI	2023	Incidence	Number	94,277	72,279	121,610	94277 (72279 to 121610)
Low-middle SDI	2023	Incidence	Weighted rate per 100,000	47.93	36.78	61.76	47.93 (36.78 to 61.76)
Low-middle SDI	2023	Deaths	Number	35,719	27,300	45,513	35719 (27300 to 45513)
Low-middle SDI	2023	Deaths	Weighted rate per 100,000	18.21	13.93	23.16	18.21 (13.93 to 23.16)
Low-middle SDI	2023	YLLs	Number	1,776,657	1,353,717	2,272,730	1776657 (1353717 to 2272730)
Low-middle SDI	2023	YLLs	Weighted rate per 100,000	900.18	686.76	1149.54	900.18 (686.76 to 1149.54)
Low-middle SDI	2023	YLDs	Number	56,331	37,105	83,495	56331 (37105 to 83495)
Low-middle SDI	2023	YLDs	Weighted rate per 100,000	28.56	18.83	42.28	28.56 (18.83 to 42.28)
Low-middle SDI	2023	DALYs	Number	1,832,988	1,398,323	2,346,415	1832988 (1398323 to 2346415)
Low-middle SDI	2023	DALYs	Weighted rate per 100,000	928.74	709.19	1186.81	928.74 (709.19 to 1186.81)
Low SDI	1990	Incidence	Number	18,960	13,705	25,764	18960 (13705 to 25764)

Location	Year	Measure	Metric	Estimate	Lower 95% UI	Upper 95% UI	Formatted 95% UI
Low SDI	1990	Incidence	Weighted rate per 100,000	20.03	14.49	27.13	20.03 (14.49 to 27.13)
Low SDI	1990	Deaths	Number	10,386	7,606	13,945	10386 (7606 to 13945)
Low SDI	1990	Deaths	Weighted rate per 100,000	11.07	8.11	14.82	11.07 (8.11 to 14.82)
Low SDI	1990	YLLs	Number	522,958	382,710	703,877	522958 (382710 to 703877)
Low SDI	1990	YLLs	Weighted rate per 100,000	547.62	401.12	734.83	547.62 (401.12 to 734.83)
Low SDI	1990	YLDs	Number	12,211	7,422	18,363	12211 (7422 to 18363)
Low SDI	1990	YLDs	Weighted rate per 100,000	12.83	7.82	19.2	12.83 (7.82 to 19.2)
Low SDI	1990	DALYs	Number	535,168	390,895	720,916	535168 (390895 to 720916)
Low SDI	1990	DALYs	Weighted rate per 100,000	560.45	409.8	752.47	560.45 (409.8 to 752.47)
Low SDI	2023	Incidence	Number	125,121	91,810	164,463	125121 (91810 to 164463)
Low SDI	2023	Incidence	Weighted rate per 100,000	51.79	38.11	68.04	51.79 (38.11 to 68.04)
Low SDI	2023	Deaths	Number	52,991	38,156	68,590	52991 (38156 to 68590)
Low SDI	2023	Deaths	Weighted rate per 100,000	22.09	15.93	28.57	22.09 (15.93 to 28.57)
Low SDI	2023	YLLs	Number	2,650,740	1,905,119	3,433,049	2650740 (1905119 to 3433049)
Low SDI	2023	YLLs	Weighted rate per 100,000	1089.89	784.79	1410.79	1089.89 (784.79 to 1410.79)
Low SDI	2023	YLDs	Number	76,759	47,065	115,405	76759 (47065 to 115405)
Low SDI	2023	YLDs	Weighted rate per 100,000	31.62	19.4	47.44	31.62 (19.4 to 47.44)
Low SDI	2023	DALYs	Number	2,727,499	1,962,993	3,543,003	2727499 (1962993 to 3543003)
Low SDI	2023	DALYs	Weighted rate per 100,000	1121.52	808.43	1456.02	1121.52 (808.43 to 1456.02)

Note: *Counts and weighted rates for ages 25–49 years are shown with GBD 95% uncertainty intervals. Weighted rates were recalculated by the authors from the five age-specific groups. UI, uncertainty interval.*

Supplementary Table S15. Age-specific PAFs in selected years

Year	Age group	Risk factor	PAF	Lower 95% UI	Upper 95% UI	Source status
1990	25-29 years	High BMI	0	0	0	Observed zero
2000	25-29 years	High BMI	0	0	0	Observed zero
2010	25-29 years	High BMI	0	0	0	Observed zero
2020	25-29 years	High BMI	0	0	0	Observed zero
2023	25-29 years	High BMI	0	0	0	Observed zero
1990	30-34 years	High BMI	0	0	0	Observed zero
2000	30-34 years	High BMI	0	0	0	Observed zero
2010	30-34 years	High BMI	0	0	0	Observed zero
2020	30-34 years	High BMI	0	0	0	Observed zero
2023	30-34 years	High BMI	0	0	0	Observed zero
1990	35-39 years	High BMI	0	0	0	Observed zero
2000	35-39 years	High BMI	0	0	0	Observed zero
2010	35-39 years	High BMI	0	0	0	Observed zero
2020	35-39 years	High BMI	0	0	0	Observed zero
2023	35-39 years	High BMI	0	0	0	Observed zero
1990	40-44 years	High BMI	0	0	0	Observed zero
2000	40-44 years	High BMI	0	0	0	Observed zero
2010	40-44 years	High BMI	0	0	0	Observed zero
2020	40-44 years	High BMI	0	0	0	Observed zero
2023	40-44 years	High BMI	0	0	0	Observed zero
1990	45-49 years	High BMI	0	0	0	Observed zero
2000	45-49 years	High BMI	0	0	0	Observed zero
2010	45-49 years	High BMI	0	0	0	Observed zero
2020	45-49 years	High BMI	0	0	0	Observed zero
2023	45-49 years	High BMI	0	0	0	Observed zero
1990	25-29 years	High alcohol use	0.0087	0.0036	0.0134	Observed positive
2000	25-29 years	High alcohol use	0.0071	0.0038	0.0116	Observed positive
2010	25-29 years	High alcohol use	0.0102	0.0055	0.0175	Observed positive
2020	25-29 years	High alcohol use	0.0121	0.0047	0.0212	Observed positive
2023	25-29 years	High alcohol use	0.0128	0.0059	0.0231	Observed positive
1990	30-34 years	High alcohol use	0.0113	0.0056	0.018	Observed positive
2000	30-34 years	High alcohol use	0.0102	0.0057	0.0159	Observed positive
2010	30-34 years	High alcohol use	0.0133	0.0078	0.0201	Observed positive
2020	30-34 years	High alcohol use	0.0141	0.0064	0.0221	Observed positive
2023	30-34 years	High alcohol use	0.0145	0.007	0.0232	Observed positive
1990	35-39 years	High alcohol use	0.0144	0.0076	0.0231	Observed positive
2000	35-39 years	High alcohol use	0.0119	0.0066	0.0182	Observed positive
2010	35-39 years	High alcohol use	0.0134	0.0075	0.0211	Observed positive
2020	35-39 years	High alcohol use	0.0142	0.0062	0.0227	Observed positive

Year	Age group	Risk factor	PAF	Lower 95% UI	Upper 95% UI	Source status
2023	35-39 years	High alcohol use	0.0146	0.0068	0.0234	Observed positive
1990	40-44 years	High alcohol use	0.0174	0.0088	0.0279	Observed positive
2000	40-44 years	High alcohol use	0.0145	0.0074	0.0226	Observed positive
2010	40-44 years	High alcohol use	0.0159	0.0081	0.0251	Observed positive
2020	40-44 years	High alcohol use	0.016	0.0064	0.0262	Observed positive
2023	40-44 years	High alcohol use	0.0162	0.0067	0.0271	Observed positive
1990	45-49 years	High alcohol use	0.0168	0.0067	0.0273	Observed positive
2000	45-49 years	High alcohol use	0.0125	0.0046	0.0211	Observed positive
2010	45-49 years	High alcohol use	0.0134	0.0054	0.024	Observed positive
2020	45-49 years	High alcohol use	0.014	0.005	0.0247	Observed positive
2023	45-49 years	High alcohol use	0.0145	0.005	0.027	Observed positive
1990	25-29 years	High fasting plasma glucose	0.0371	0.0164	0.0757	Observed positive
2000	25-29 years	High fasting plasma glucose	0.0286	0.0131	0.0568	Observed positive
2010	25-29 years	High fasting plasma glucose	0.0228	0.0113	0.0422	Observed positive
2020	25-29 years	High fasting plasma glucose	0.0237	0.0108	0.0457	Observed positive
2023	25-29 years	High fasting plasma glucose	0.025	0.0115	0.0485	Observed positive
1990	30-34 years	High fasting plasma glucose	0.0451	0.0224	0.087	Observed positive
2000	30-34 years	High fasting plasma glucose	0.0364	0.0183	0.0676	Observed positive
2010	30-34 years	High fasting plasma glucose	0.0296	0.0158	0.0522	Observed positive
2020	30-34 years	High fasting plasma glucose	0.0306	0.016	0.0559	Observed positive
2023	30-34 years	High fasting plasma glucose	0.032	0.0164	0.0589	Observed positive
1990	35-39 years	High fasting plasma glucose	0.0543	0.0285	0.0986	Observed positive
2000	35-39 years	High fasting plasma glucose	0.0457	0.0248	0.0796	Observed positive
2010	35-39 years	High fasting plasma glucose	0.0375	0.0219	0.0631	Observed positive
2020	35-39 years	High fasting plasma glucose	0.0386	0.0214	0.0668	Observed positive
2023	35-39 years	High fasting plasma glucose	0.0405	0.0222	0.0704	Observed positive
1990	40-44 years	High fasting plasma glucose	0.0634	0.0326	0.1068	Observed positive
2000	40-44 years	High fasting plasma glucose	0.0549	0.0323	0.089	Observed positive
2010	40-44 years	High fasting plasma glucose	0.0452	0.0294	0.0711	Observed positive
2020	40-44 years	High fasting plasma glucose	0.0455	0.029	0.0727	Observed positive
2023	40-44 years	High fasting plasma glucose	0.0477	0.0286	0.0776	Observed positive
1990	45-49 years	High fasting plasma glucose	0.071	0.0393	0.1137	Observed positive
2000	45-49 years	High fasting plasma glucose	0.0629	0.0396	0.0975	Observed positive
2010	45-49 years	High fasting plasma glucose	0.0534	0.0371	0.0788	Observed positive
2020	45-49 years	High fasting plasma glucose	0.0534	0.0353	0.0815	Observed positive
2023	45-49 years	High fasting plasma glucose	0.0556	0.0353	0.0836	Observed positive
1990	25-29 years	Low physical activity	0.0112	0.0012	0.0275	Observed positive
2000	25-29 years	Low physical activity	0.0105	0.0015	0.0239	Observed positive
2010	25-29 years	Low physical activity	0.0117	0.0021	0.0272	Observed positive
2020	25-29 years	Low physical activity	0.0123	0.0017	0.0295	Observed positive
2023	25-29 years	Low physical activity	0.0125	0.0015	0.032	Observed positive

Year	Age group	Risk factor	PAF	Lower 95% UI	Upper 95% UI	Source status
1990	30-34 years	Low physical activity	0.0111	0.001	0.0262	Observed positive
2000	30-34 years	Low physical activity	0.0106	0.0016	0.0224	Observed positive
2010	30-34 years	Low physical activity	0.0114	0.0018	0.0254	Observed positive
2020	30-34 years	Low physical activity	0.0126	0.0015	0.0322	Observed positive
2023	30-34 years	Low physical activity	0.0124	0.0014	0.0327	Observed positive
1990	35-39 years	Low physical activity	0.011	0.0014	0.0295	Observed positive
2000	35-39 years	Low physical activity	0.0106	0.0016	0.0234	Observed positive
2010	35-39 years	Low physical activity	0.0107	0.0021	0.0239	Observed positive
2020	35-39 years	Low physical activity	0.0118	0.0013	0.0303	Observed positive
2023	35-39 years	Low physical activity	0.012	0.0013	0.0293	Observed positive
1990	40-44 years	Low physical activity	0.0112	0.0013	0.0279	Observed positive
2000	40-44 years	Low physical activity	0.0107	0.0017	0.0241	Observed positive
2010	40-44 years	Low physical activity	0.0113	0.0018	0.0274	Observed positive
2020	40-44 years	Low physical activity	0.0125	0.0013	0.0298	Observed positive
2023	40-44 years	Low physical activity	0.0127	0.0013	0.0289	Observed positive
1990	45-49 years	Low physical activity	0.0121	0.0016	0.0294	Observed positive
2000	45-49 years	Low physical activity	0.0115	0.0019	0.0262	Observed positive
2010	45-49 years	Low physical activity	0.0118	0.0016	0.0283	Observed positive
2020	45-49 years	Low physical activity	0.0129	0.0016	0.0297	Observed positive
2023	45-49 years	Low physical activity	0.0132	0.0016	0.0306	Observed positive
1990	25-29 years	Tobacco use	0.1365	0.0874	0.1839	Observed positive
2000	25-29 years	Tobacco use	0.1322	0.084	0.1812	Observed positive
2010	25-29 years	Tobacco use	0.1295	0.082	0.1769	Observed positive
2020	25-29 years	Tobacco use	0.1275	0.0816	0.174	Observed positive
2023	25-29 years	Tobacco use	0.1266	0.0811	0.174	Observed positive
1990	30-34 years	Tobacco use	0.1374	0.0888	0.1832	Observed positive
2000	30-34 years	Tobacco use	0.1331	0.0873	0.1816	Observed positive
2010	30-34 years	Tobacco use	0.1306	0.0835	0.1785	Observed positive
2020	30-34 years	Tobacco use	0.1283	0.0829	0.174	Observed positive
2023	30-34 years	Tobacco use	0.1274	0.0825	0.1747	Observed positive
1990	35-39 years	Tobacco use	0.138	0.0895	0.1833	Observed positive
2000	35-39 years	Tobacco use	0.1334	0.0878	0.181	Observed positive
2010	35-39 years	Tobacco use	0.1301	0.0834	0.177	Observed positive
2020	35-39 years	Tobacco use	0.1279	0.0825	0.1732	Observed positive
2023	35-39 years	Tobacco use	0.127	0.0822	0.1739	Observed positive
1990	40-44 years	Tobacco use	0.1403	0.0905	0.1862	Observed positive
2000	40-44 years	Tobacco use	0.1359	0.0895	0.1833	Observed positive
2010	40-44 years	Tobacco use	0.1339	0.0883	0.1819	Observed positive
2020	40-44 years	Tobacco use	0.1309	0.0849	0.1779	Observed positive
2023	40-44 years	Tobacco use	0.1299	0.0851	0.1772	Observed positive
1990	45-49 years	Tobacco use	0.142	0.0922	0.1871	Observed positive

Year	Age group	Risk factor	PAF	Lower 95% UI	Upper 95% UI	Source status
2000	45-49 years	Tobacco use	0.1367	0.0904	0.1833	Observed positive
2010	45-49 years	Tobacco use	0.1326	0.0867	0.1793	Observed positive
2020	45-49 years	Tobacco use	0.1304	0.0856	0.1749	Observed positive
2023	45-49 years	Tobacco use	0.1296	0.0854	0.1749	Observed positive

Note: *PAFs are shown as proportions for selected calendar years and age groups. The selected China-specific high-BMI series is an observed constant-zero series; this does not indicate absent obesity exposure or biological irrelevance. PAF, population attributable fraction.*

Supplementary Table S16. Mortality-to-incidence ratios in selected years

Location	Year	Deaths	Incidence	Crude count ratio	Weighted ASIR, 25–49	Weighted ASDR, 25–49	Weighted rate MIR	Difference	Relative difference (%)
China	1990	16,414	50,064	0.328	28.06	9.25	0.33	0.002	0.52
China	2000	21,834	79,779	0.274	32.37	8.84	0.273	-0	-0.16
China	2010	17,466	87,103	0.201	28.96	5.79	0.2	-0	-0.25
China	2020	12,472	81,710	0.153	28.89	4.39	0.152	-0.001	-0.53
China	2023	12,611	87,221	0.145	32.87	4.72	0.144	-0.001	-0.68
Global	1990	84,800	278,566	0.304	36.39	11.07	0.304	-0	-0.09
Global	2000	107,585	390,469	0.276	38.81	10.68	0.275	-0	-0.15
Global	2010	115,568	455,950	0.253	37.28	9.45	0.254	0	0.05
Global	2020	149,998	572,623	0.262	42.16	11.04	0.262	0	0
Global	2023	179,756	672,228	0.267	48.36	12.92	0.267	-0	-0.08
High SDI	1990	42,525	184,164	0.231	53.65	12.4	0.231	0	0.12
High SDI	2000	48,567	243,916	0.199	55.69	11.07	0.199	-0	-0.14
High SDI	2010	40,488	250,433	0.162	51.27	8.27	0.161	-0	-0.18
High SDI	2020	35,719	242,029	0.148	49.73	7.31	0.147	-0.001	-0.37
High SDI	2023	36,344	250,244	0.145	52.08	7.53	0.145	-0.001	-0.46
High-middle SDI	1990	15,466	40,701	0.38	25.23	9.65	0.382	0.003	0.66
High-middle SDI	2000	21,480	65,114	0.33	29.3	9.69	0.331	0.001	0.25
High-middle SDI	2010	22,547	79,969	0.282	28.5	8.03	0.282	-0	-0.05
High-middle SDI	2020	25,023	99,785	0.251	32.73	8.2	0.25	-0	-0.12
High-middle SDI	2023	27,810	114,514	0.243	37.25	9.02	0.242	-0.001	-0.25
Middle SDI	1990	7,565	17,215	0.439	21.96	9.73	0.443	0.004	0.88
Middle SDI	2000	11,580	29,229	0.396	26.43	10.54	0.399	0.003	0.65
Middle SDI	2010	16,600	46,443	0.357	32.12	11.5	0.358	0.001	0.15
Middle SDI	2020	23,168	72,464	0.32	43.23	13.82	0.32	-0	-0.06
Middle SDI	2023	26,739	87,495	0.306	50.49	15.41	0.305	-0	-0.12
Low-middle SDI	1990	8,760	17,211	0.509	18.38	9.42	0.513	0.004	0.7
Low-middle SDI	2000	12,289	25,730	0.478	21.37	10.27	0.481	0.003	0.61
Low-middle SDI	2010	17,056	39,743	0.429	25.89	11.16	0.431	0.002	0.43
Low-middle SDI	2020	28,811	73,532	0.392	39.46	15.51	0.393	0.001	0.3
Low-middle SDI	2023	35,719	94,277	0.379	47.93	18.21	0.38	0.001	0.28
Low SDI	1990	10,386	18,960	0.548	20.03	11.07	0.553	0.005	0.9
Low SDI	2000	13,556	26,070	0.52	20.96	11	0.525	0.005	0.95
Low SDI	2010	18,761	38,912	0.482	23.5	11.43	0.486	0.004	0.87
Low SDI	2020	37,138	84,281	0.441	37.97	16.85	0.444	0.003	0.69
Low SDI	2023	52,991	125,121	0.424	51.79	22.09	0.426	0.003	0.69

Note: *Weighted rate MIR is calculated as weighted ASDR divided by weighted ASIR for ages 25–49 years; the crude deaths-to-incidence count ratio is a separate quantity. MIR should not be interpreted as individual survival. ASIR, age-standardized incidence rate; ASDR, age-standardized death rate.*

Supplementary Table S17. Data provenance

Output	Source or derivation	Author-derived
Table 1 burden estimates	GBD age-specific counts/rates; ages 25-49 aggregated or weighted by the authors	Yes
EAPC	Log-linear regression fitted by the authors to recalculated weighted rates	Yes
Decomposition	GBD incidence counts/rates with author-constructed demographic counterfactuals	Yes
Risk PAF trends	GBD Comparative Risk Assessment PAF estimates; age-specific values used directly and broad trends summarized by the authors using unweighted arithmetic means across the five age groups	Partly
XGBoost-SHAP ranking	Author-fitted exploratory model using GBD-derived DALY PAF features	Yes
MIR	Author-calculated ratio; Figure 4 uses weighted ASDR divided by weighted ASIR for ages 25-49	Yes
HCA potential mortality-related productivity loss	GBD total YLLs (not truncated at a prespecified working-age or retirement-age boundary) × World Bank GDP per capita × ILO age-specific female LFPR	Yes
Risk-attributable potential mortality-related productivity loss	HCA potential mortality-related productivity loss multiplied by YLL-specific GBD PAFs	Yes
BAPC projection	Author-fitted BAPC model using historical GBD incidence and population denominators	Yes
GLOBOCAN comparison	GLOBOCAN 2022 counts converted to rates and compared with GBD 2022 rates	Yes

Note: *This table summarizes the source or derivation of key study outputs and indicates whether each output is fully or partly author-derived.*
