# Supplementary material for: Breast cancer burden risk attribution and productivity loss in Chinese women aged 25 to 49
Source: iScience. 2026 Oct 14;29(11):117891. doi: 10.1016/j.isci.2026.117891 (PMC13683865; doi:10.1016/j.isci.2026.117891)

Sensitivity analysis using rates derived from GLOBOCAN 2022 case estimates as an external incidence baseline

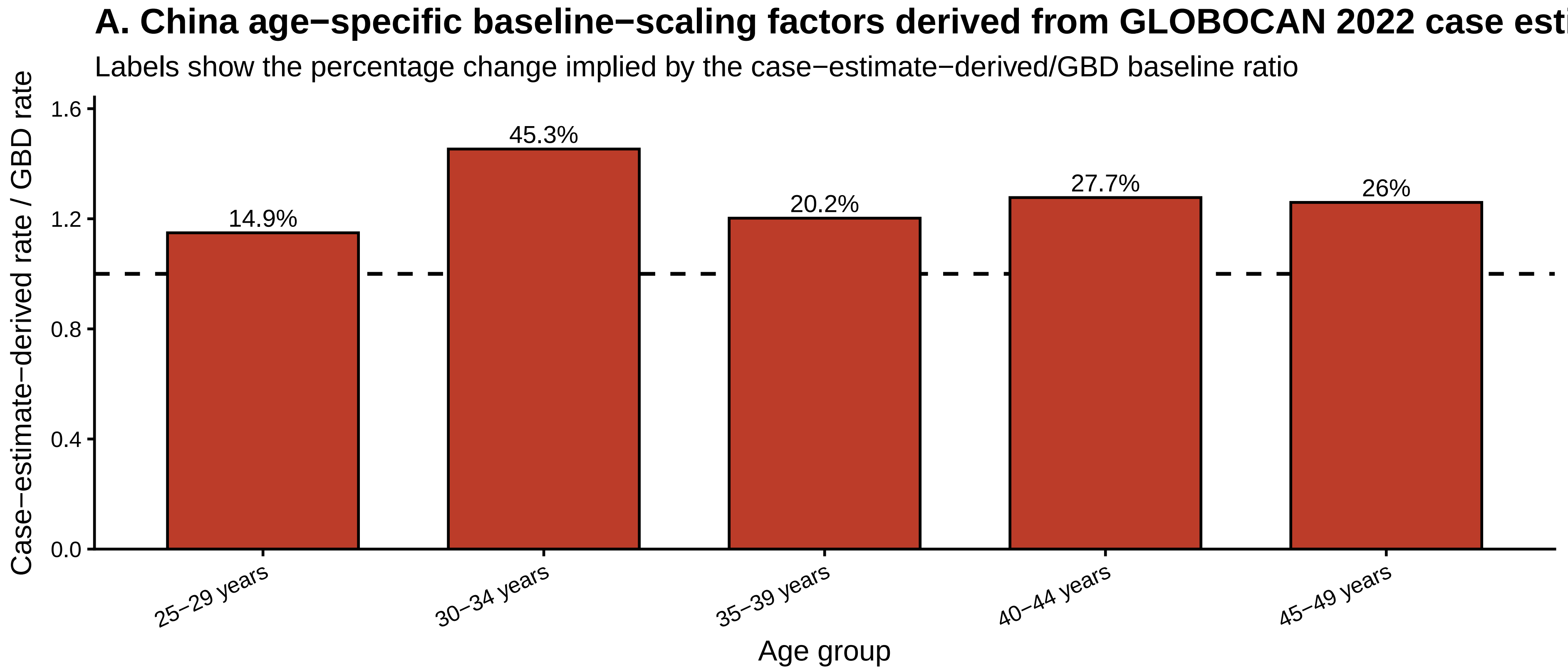

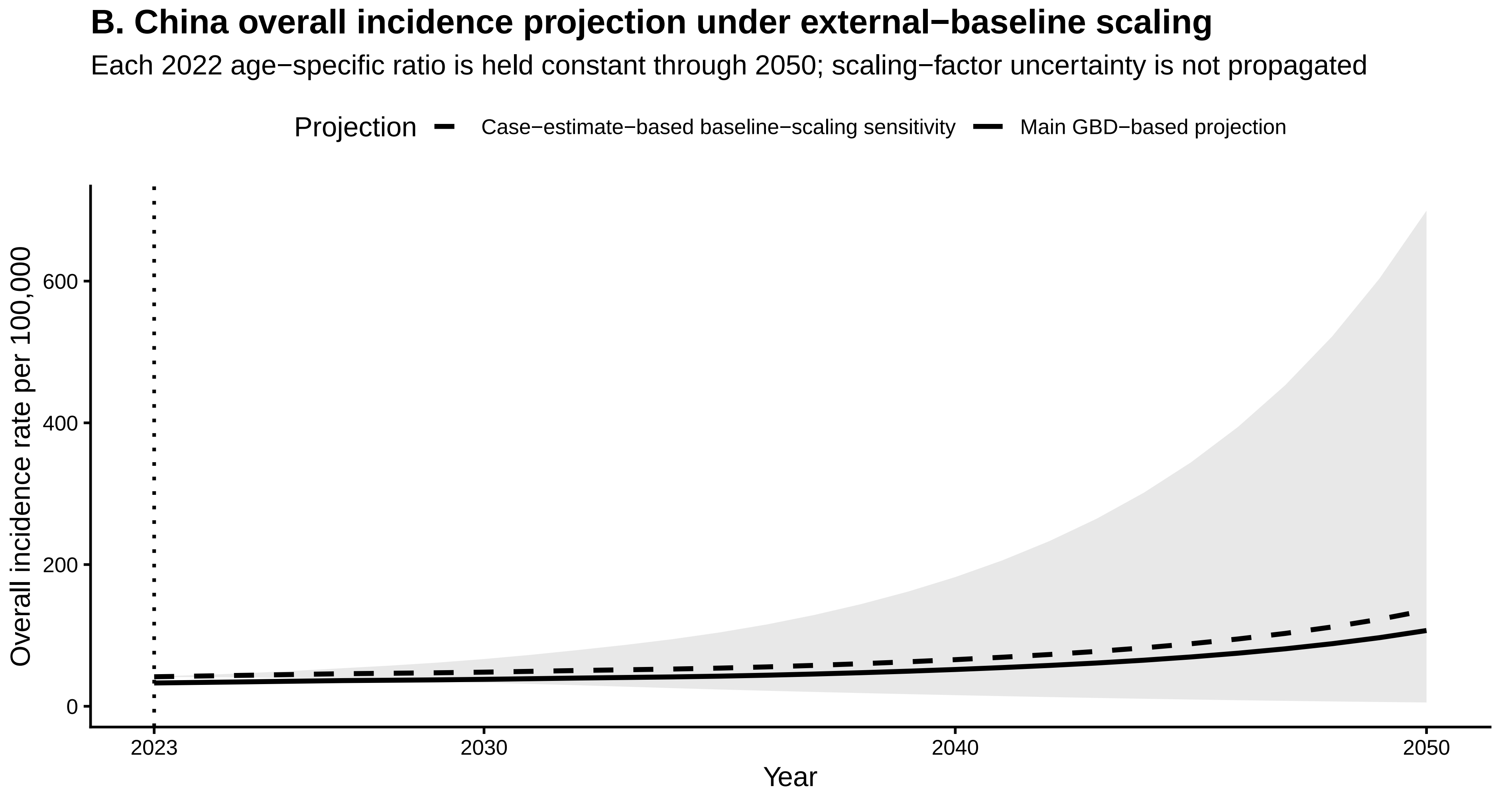

Supplement: Supplementary file 2 [file mmc2.zip › GBD_EOBC_final_release_v1.1.10_CLEAN/Results/Analysis_Figures/GLOBOCANBaseline_GLOBOCAN_baseline_scaled_projection.pdf]
