# Supplementary material for: Breast cancer burden risk attribution and productivity loss in Chinese women aged 25 to 49
Source: iScience. 2026 Oct 14;29(11):117891. doi: 10.1016/j.isci.2026.117891 (PMC13683865; doi:10.1016/j.isci.2026.117891)
Supplement: Supplementary file 2 [file mmc2.zip › GBD_EOBC_final_release_v1.1.10_CLEAN/Results/Analysis_Figures/HCASensitivity_HCA_sensitivity.pdf]

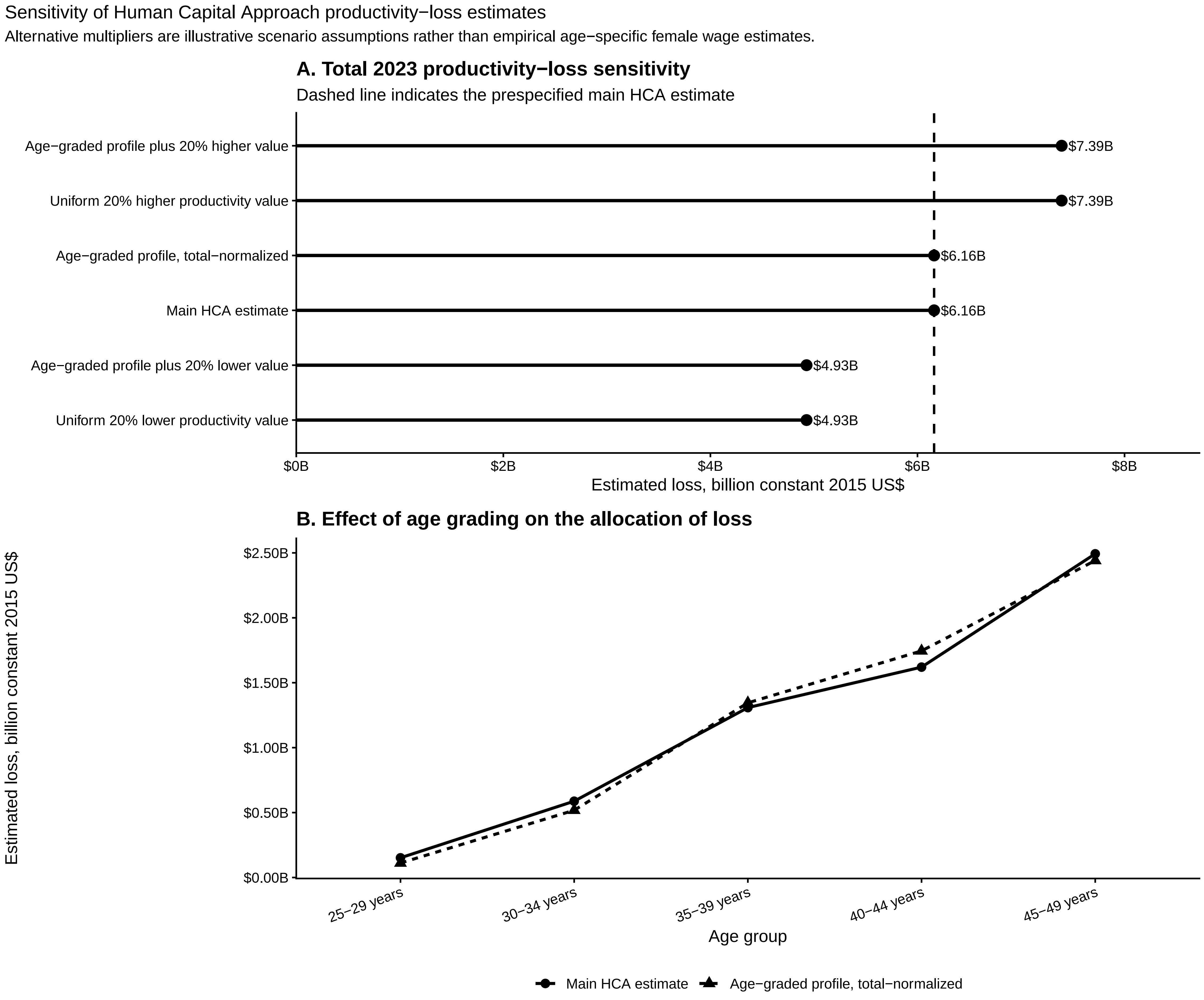

Sensitivity of Human Capital Approach productivity−loss estimates
Alternative multipliers are illustrative scenario assumptions rather than empirical age−specific female wage estimates.
A. Total 2023 productivity−loss sensitivity
Dashed line indicates the prespecified main HCA estimate
Age−graded profile plus 20% higher value
$7.39B
Uniform 20% higher productivity value
$7.39B
Age−graded profile, total−normalized
$6.16B
Main HCA estimate
$6.16B
Age−graded profile plus 20% lower value
$4.93B
Uniform 20% lower productivity value
$4.93B
$0B
$2B
$4B
$6B
$8B
Estimated loss, billion constant 2015 US$
B. Effect of age grading on the allocation of loss
Estimated loss, billion constant 2015 US$
$2.50B
$2.00B
$1.50B
$1.00B
$0.50B
$0.00B
25−29 years
30−34 years
35−39 years
40−44 years
45−49 years
Age group
Main HCA estimate
Age−graded profile, total−normalized
