# Supplementary material for: Breast cancer burden risk attribution and productivity loss in Chinese women aged 25 to 49
Source: iScience. 2026 Oct 14;29(11):117891. doi: 10.1016/j.isci.2026.117891 (PMC13683865; doi:10.1016/j.isci.2026.117891)
Supplement: Supplementary file 2 [file mmc2.zip › GBD_EOBC_final_release_v1.1.10_CLEAN/Results/Analysis_Figures/ProjectionAnchor_anchored_vs_unanchored_projection.pdf]

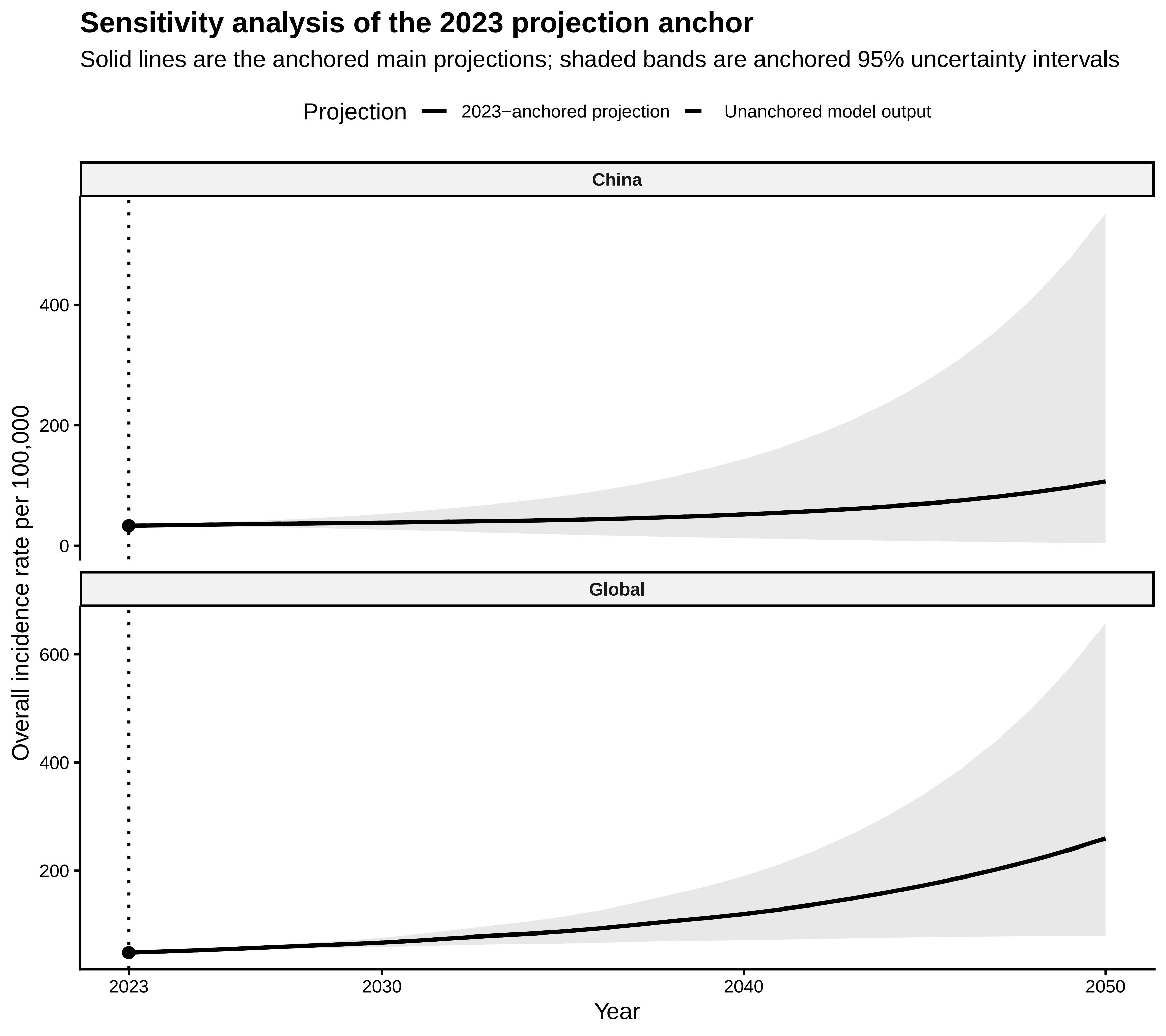

Sensitivity analysis of the 2023 projection anchor
Solid lines are the anchored main projections; shaded bands are anchored 95% uncertainty intervals
Projection
2023−anchored projection
Unanchored model output
China
Global
Overall incidence rate per 100,000
0
200
400
600
2023
2030
2040
2050
Year
