# Supplementary material for: Breast cancer burden risk attribution and productivity loss in Chinese women aged 25 to 49
Source: iScience. 2026 Oct 14;29(11):117891. doi: 10.1016/j.isci.2026.117891 (PMC13683865; doi:10.1016/j.isci.2026.117891)
Supplement: Supplementary file 2 [file mmc2.zip › GBD_EOBC_final_release_v1.1.10_CLEAN/Results/Analysis_Figures/SHAPStructure_age_structure_target_sensitivity.pdf]

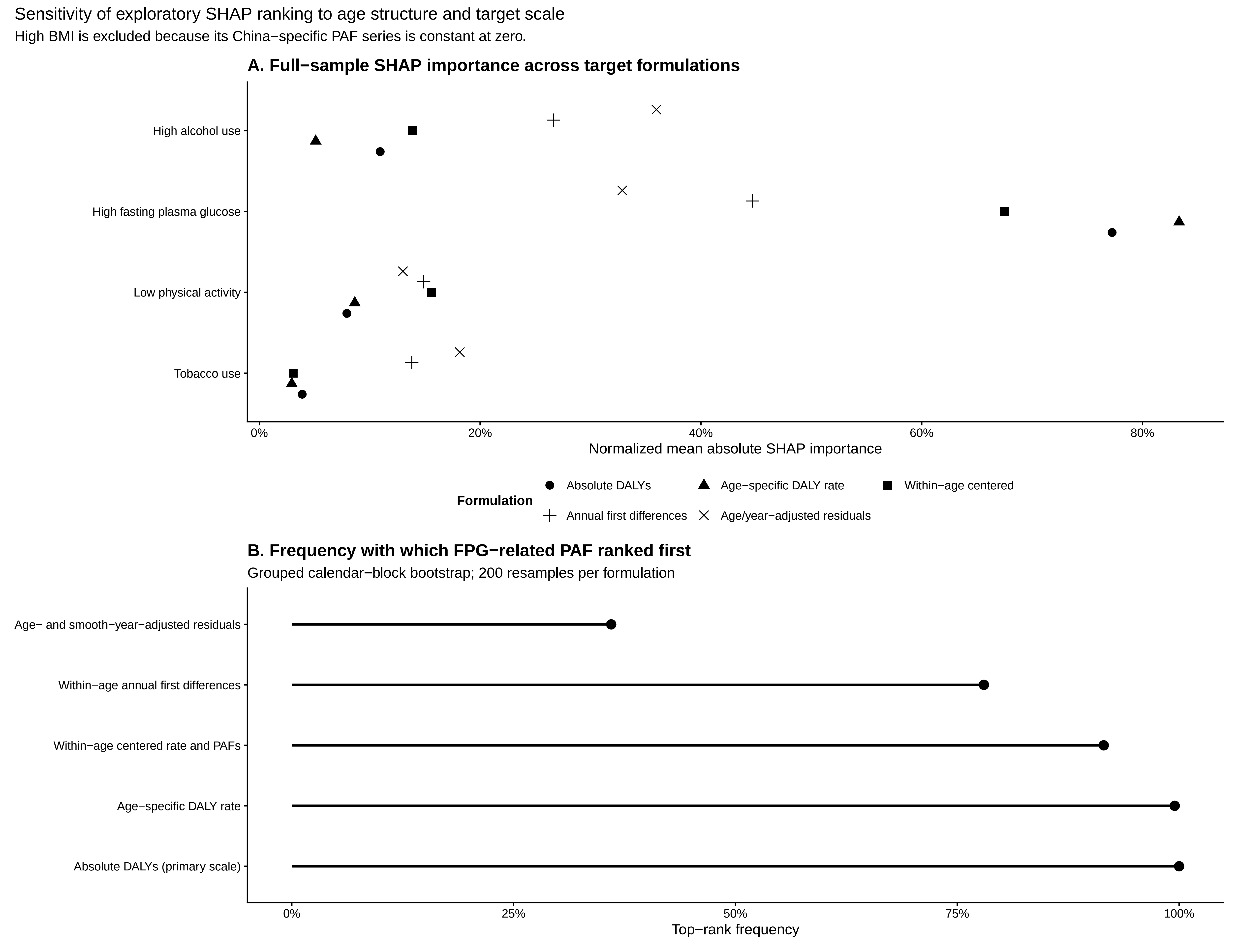

Sensitivity of exploratory SHAP ranking to age structure and target scale
High BMI is excluded because its China−specific PAF series is constant at zero.
A. Full−sample SHAP importance across target formulations
High alcohol use
High fasting plasma glucose
Low physical activity
Tobacco use
0%
20%
40%
60%
80%
Normalized mean absolute SHAP importance
Formulation
Absolute DALYs
Age−specific DALY rate
Within−age centered
Annual first differences
Age/year−adjusted residuals
B. Frequency with which FPG−related PAF ranked first
Grouped calendar−block bootstrap; 200 resamples per formulation
Age− and smooth−year−adjusted residuals
Within−age annual first differences
Within−age centered rate and PAFs
Age−specific DALY rate
Absolute DALYs (primary scale)
0%
25%
50%
75%
100%
Top−rank frequency
