# Supplementary material for: Breast cancer burden risk attribution and productivity loss in Chinese women aged 25 to 49
Source: iScience. 2026 Oct 14;29(11):117891. doi: 10.1016/j.isci.2026.117891 (PMC13683865; doi:10.1016/j.isci.2026.117891)
Supplement: Supplementary file 2 [file mmc2.zip › GBD_EOBC_final_release_v1.1.10_CLEAN/Results/Manuscript_Ready/Main_Figures/Figure_1_Breast_cancer_burden_and_temporal_trends.pdf]

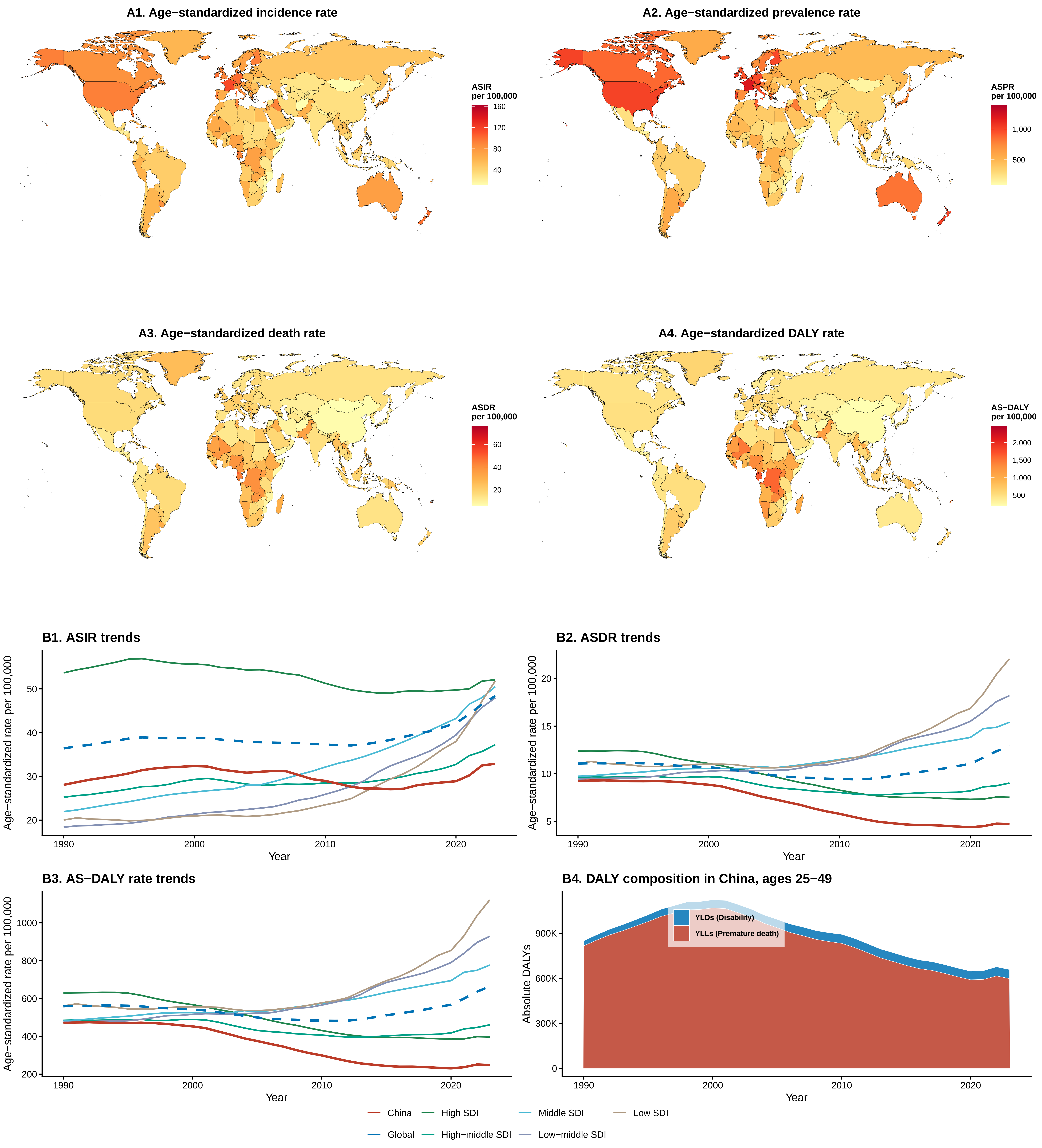
A1. Age−standardized incidence rate
ASIR
per 100,000
160
120
80
40
A2. Age−standardized prevalence rate
ASPR
per 100,000
1,000
500
A3. Age−standardized death rate
ASDR
per 100,000
60
40
20
A4. Age−standardized DALY rate
AS−DALY
per 100,000
2,000
1,500
1,000
500
B1. ASIR trends
Age−standardized rate per 100,000
50
40
30
20
1990
2000
2010
2020
Year
B2. ASDR trends
Age−standardized rate per 100,000
20
15
10
5
1990
2000
2010
2020
Year
B3. AS−DALY rate trends
Age−standardized rate per 100,000
1000
800
600
400
200
1990
2000
2010
2020
Year
B4. DALY composition in China, ages 25−49
Absolute DALYs
900K
600K
300K
0
YLDs (Disability)
YLLs (Premature death)
1990
2000
2010
2020
Year
China
High SDI
Middle SDI
Low SDI
Global
High−middle SDI
Low−middle SDI
