# Supplementary material for: Breast cancer burden risk attribution and productivity loss in Chinese women aged 25 to 49
Source: iScience. 2026 Oct 14;29(11):117891. doi: 10.1016/j.isci.2026.117891 (PMC13683865; doi:10.1016/j.isci.2026.117891)
Supplement: Supplementary file 2 [file mmc2.zip › GBD_EOBC_final_release_v1.1.10_CLEAN/Results/Manuscript_Ready/Main_Figures/Figure_3_GBD_risk_attribution_and_exploratory_SHAP_ranking.pdf]

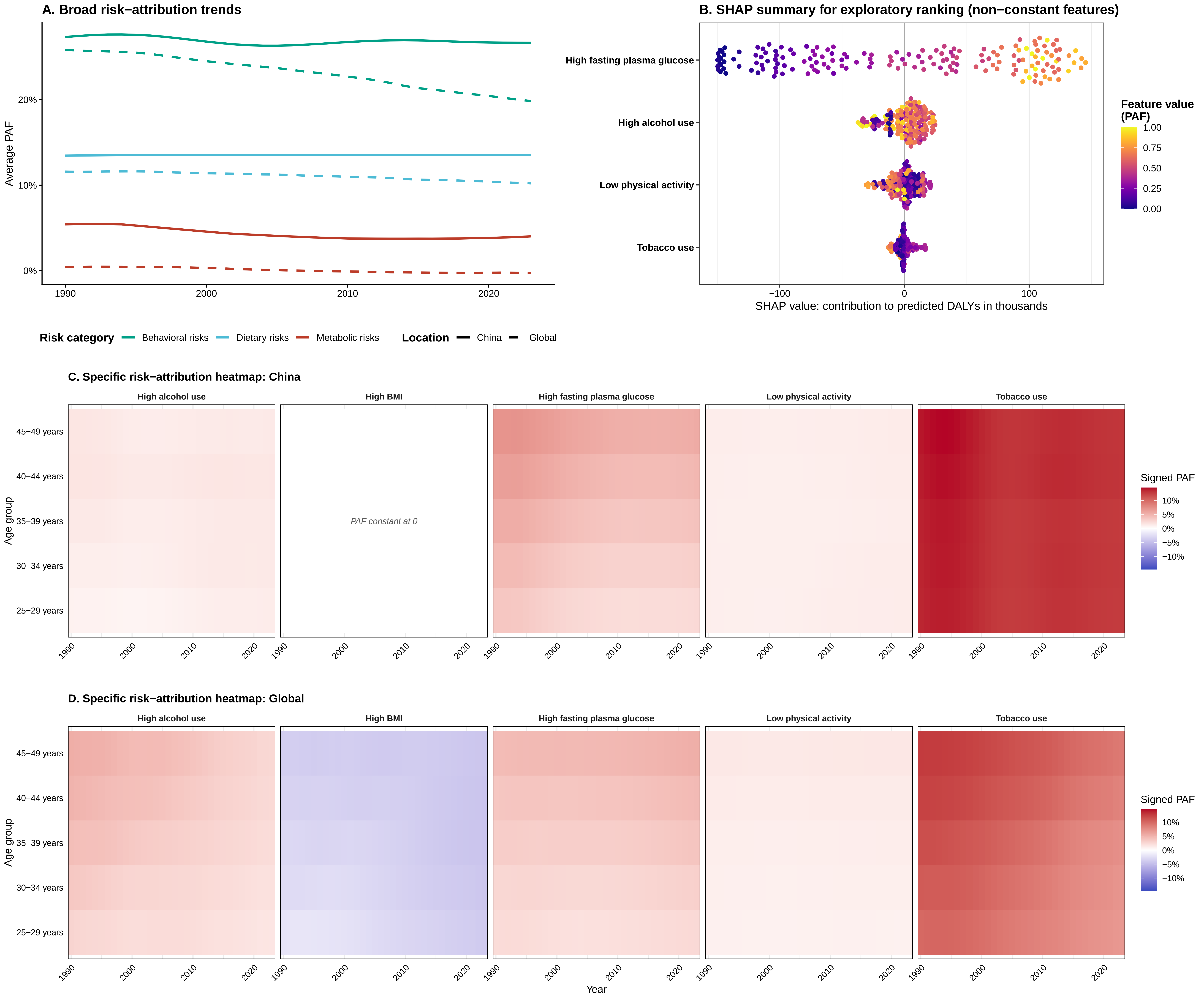
A. Broad risk−attribution trends
Average PAF
20%
10%
0%
1990
2000
2010
2020
B. SHAP summary for exploratory ranking (non−constant features)
High fasting plasma glucose
High alcohol use
Low physical activity
Tobacco use
−100
0
100
SHAP value: contribution to predicted DALYs in thousands
Feature value (PAF)
1.00
0.75
0.50
0.25
0.00
Risk category
Behavioral risks
Dietary risks
Metabolic risks
Location
China
Global
C. Specific risk−attribution heatmap: China
High alcohol use
High BMI
High fasting plasma glucose
Low physical activity
Tobacco use
45−49 years
40−44 years
35−39 years
30−34 years
25−29 years
Age group
PAF constant at 0
Signed PAF
10%
5%
0%
−5%
−10%
D. Specific risk−attribution heatmap: Global
Year
