# Supplementary material for: Breast cancer burden risk attribution and productivity loss in Chinese women aged 25 to 49
Source: iScience. 2026 Oct 14;29(11):117891. doi: 10.1016/j.isci.2026.117891 (PMC13683865; doi:10.1016/j.isci.2026.117891)
Supplement: Supplementary file 2 [file mmc2.zip › GBD_EOBC_final_release_v1.1.10_CLEAN/Results/Manuscript_Ready/Main_Figures/Figure_4_MIR_and_mortality_related_productivity_loss.pdf]

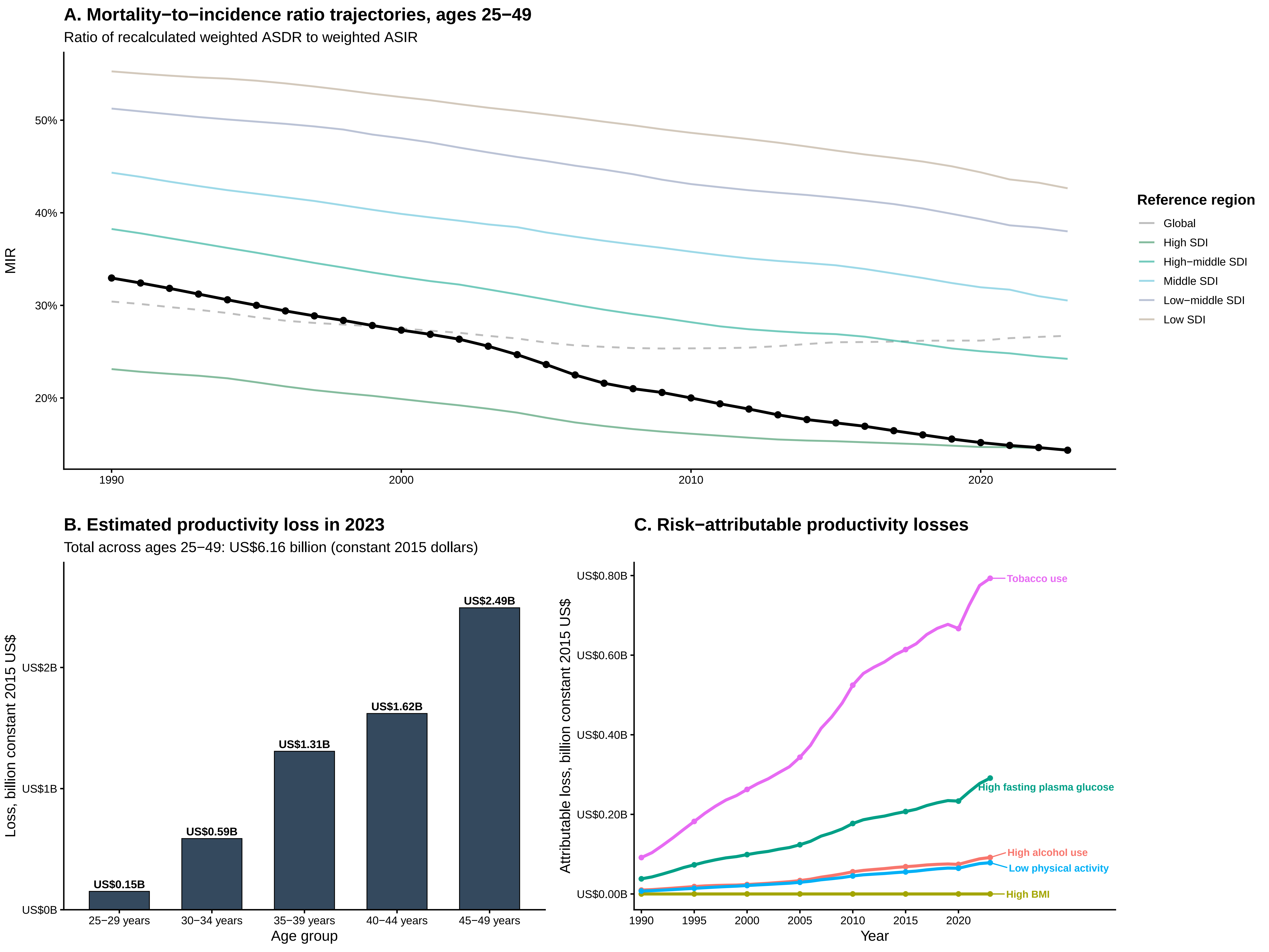

A. Mortality−to−incidence ratio trajectories, ages 25−49
Ratio of recalculated weighted ASDR to weighted ASIR
MIR
50%
40%
30%
20%
1990
2000
2010
2020
Reference region
Global
High SDI
High−middle SDI
Middle SDI
Low−middle SDI
Low SDI
B. Estimated productivity loss in 2023
Total across ages 25−49: US$6.16 billion (constant 2015 dollars)
Loss, billion constant 2015 US$
US$0B
US$1B
US$2B
US$0.15B
US$0.59B
US$1.31B
US$1.62B
US$2.49B
25−29 years
30−34 years
35−39 years
40−44 years
45−49 years
Age group
C. Risk−attributable productivity losses
Attributable loss, billion constant 2015 US$
US$0.00B
US$0.20B
US$0.40B
US$0.60B
US$0.80B
Tobacco use
High fasting plasma glucose
High alcohol use
Low physical activity
High BMI
1990
1995
2000
2005
2010
2015
2020
Year
