# Supplementary material for: Breast cancer burden risk attribution and productivity loss in Chinese women aged 25 to 49
Source: iScience. 2026 Oct 14;29(11):117891. doi: 10.1016/j.isci.2026.117891 (PMC13683865; doi:10.1016/j.isci.2026.117891)
Supplement: Supplementary file 2 [file mmc2.zip › GBD_EOBC_final_release_v1.1.10_CLEAN/Results/Manuscript_Ready/Supplementary_Figures/Supplementary_Figure_S1_SHAP_temporal_stability.pdf]

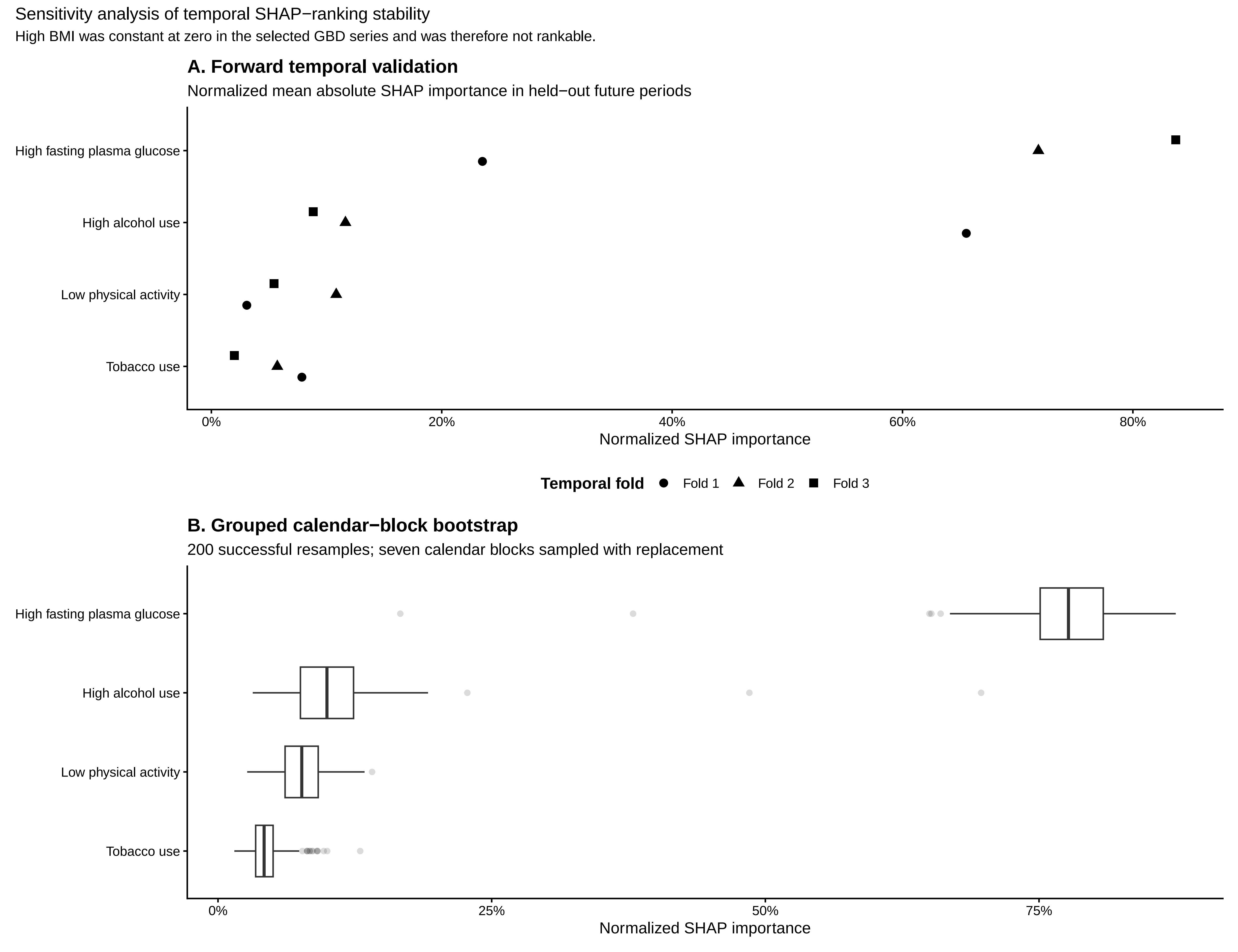
Sensitivity analysis of temporal SHAP−ranking stability
High BMI was constant at zero in the selected GBD series and was therefore not rankable.
A. Forward temporal validation
Normalized mean absolute SHAP importance in held−out future periods
High fasting plasma glucose
High alcohol use
Low physical activity
Tobacco use
0%
20%
40%
60%
80%
Normalized SHAP importance
Temporal fold
Fold 1
Fold 2
Fold 3
B. Grouped calendar−block bootstrap
200 successful resamples; seven calendar blocks sampled with replacement
High fasting plasma glucose
High alcohol use
Low physical activity
Tobacco use
0%
25%
50%
75%
Normalized SHAP importance
