# Supplementary material for: Breast cancer burden risk attribution and productivity loss in Chinese women aged 25 to 49
Source: iScience. 2026 Oct 14;29(11):117891. doi: 10.1016/j.isci.2026.117891 (PMC13683865; doi:10.1016/j.isci.2026.117891)
Supplement: Supplementary file 2 [file mmc2.zip › GBD_EOBC_final_release_v1.1.10_CLEAN/Results/Manuscript_Ready/Supplementary_Figures/Supplementary_Figure_S3_Age_specific_PAF_trends.pdf]

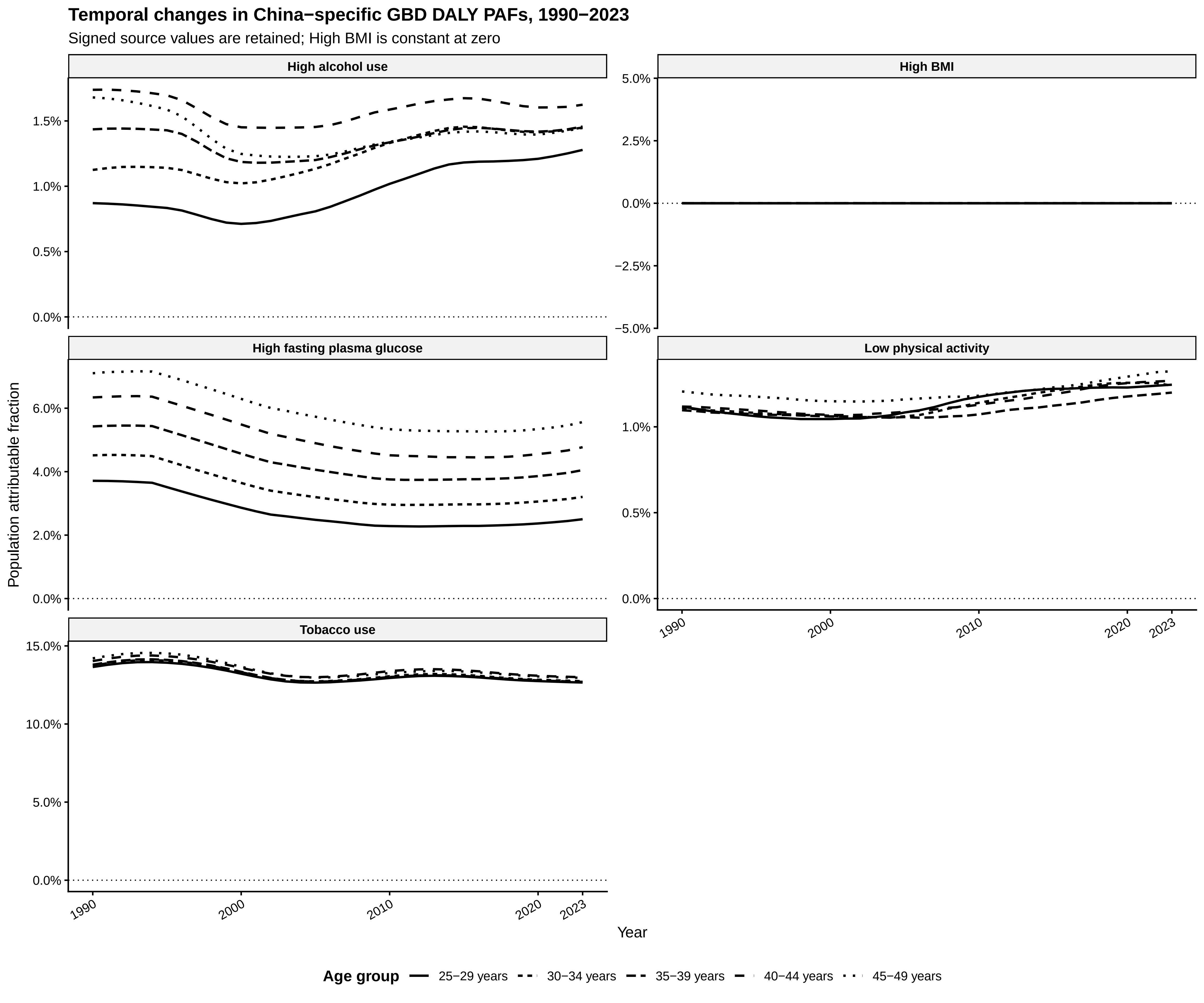

Temporal changes in China−specific GBD DALY PAFs, 1990−2023
Signed source values are retained; High BMI is constant at zero
High alcohol use
High BMI
High fasting plasma glucose
Low physical activity
Tobacco use
Population attributable fraction
Year
1990
2000
2010
2020
2023
0.0%
0.5%
1.0%
1.5%
2.0%
4.0%
6.0%
5.0%
10.0%
15.0%
−5.0%
−2.5%
2.5%
Age group
25−29 years
30−34 years
35−39 years
40−44 years
45−49 years
