# Supplementary material for: Breast cancer burden risk attribution and productivity loss in Chinese women aged 25 to 49
Source: iScience. 2026 Oct 14;29(11):117891. doi: 10.1016/j.isci.2026.117891 (PMC13683865; doi:10.1016/j.isci.2026.117891)

Sensitivity analysis using rates derived from GLOBOCAN 2022 case estimates as an external incidence baseline

A. China age−specific baseline−scaling factors derived from GLOBOCAN 2022 case estimates

Labels show the percentage change implied by the case−estimate−derived/GBD baseline ratio

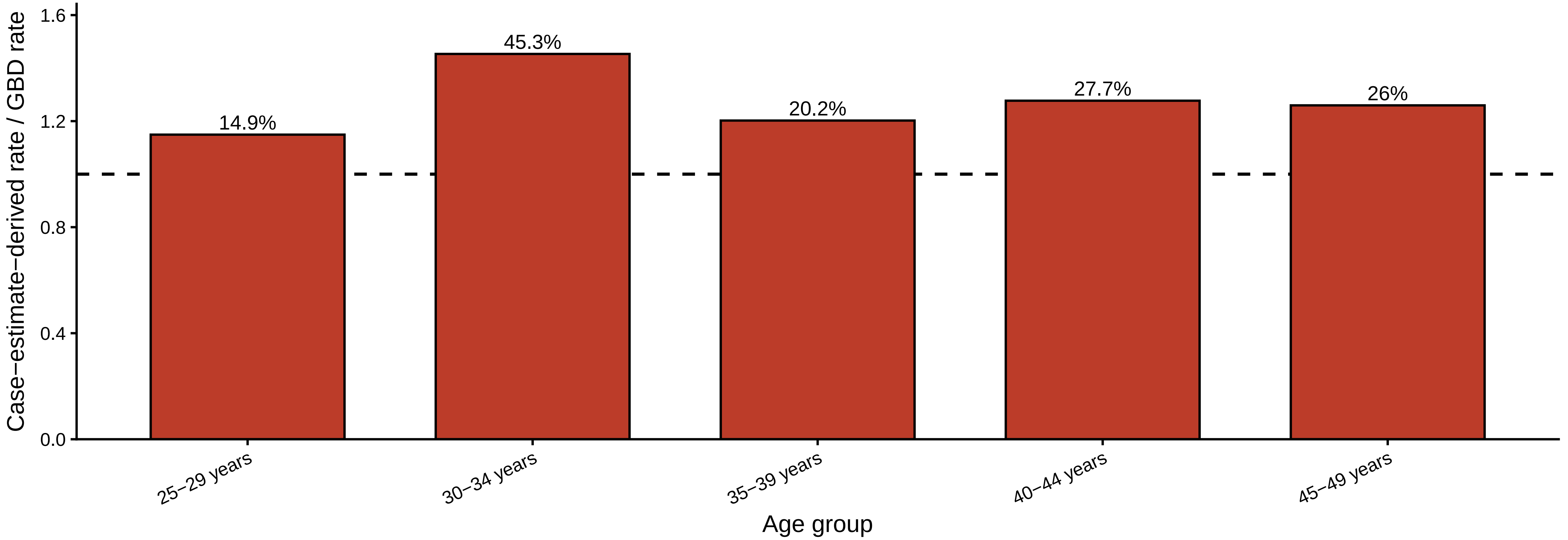

B. China overall incidence projection under external−baseline scaling

Each 2022 age−specific ratio is held constant through 2050; scaling−factor uncertainty is not propagated

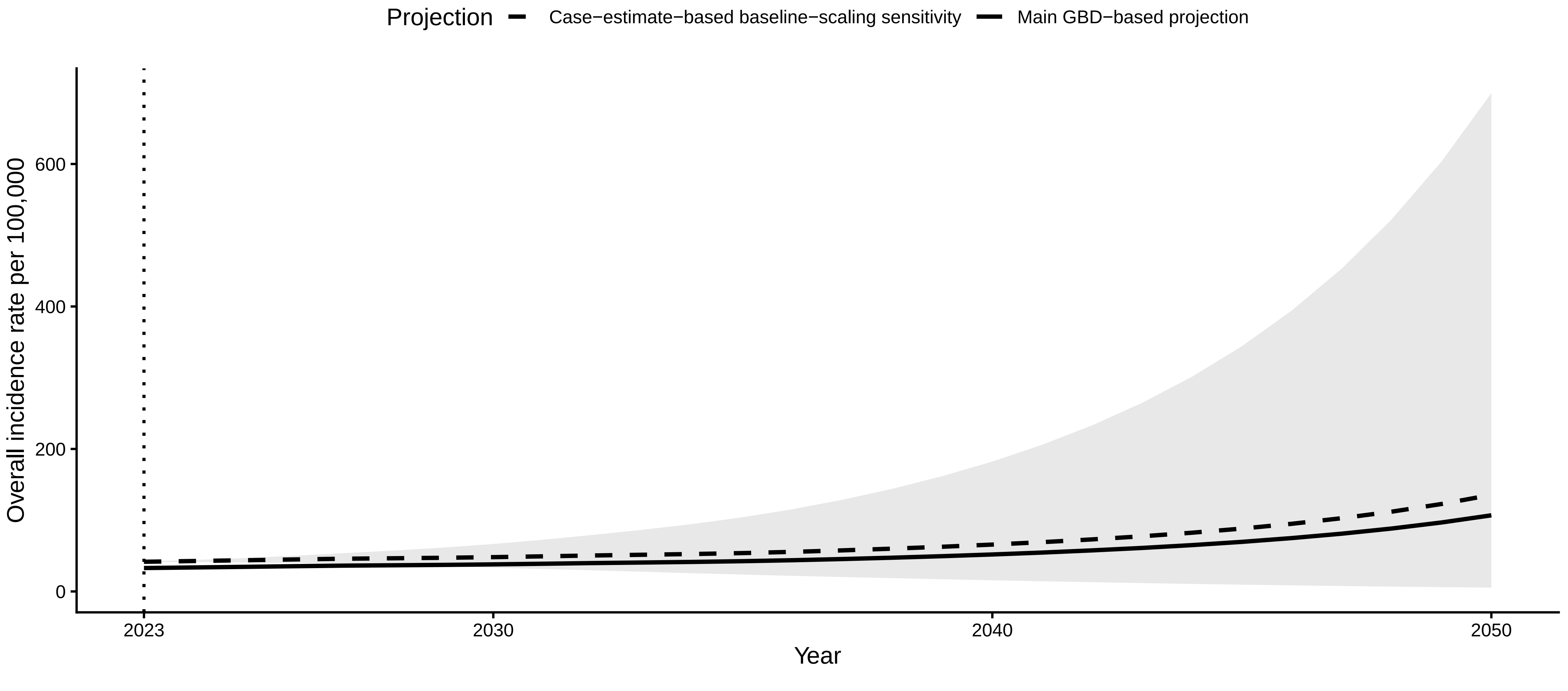

Supplement: Supplementary file 2 [file mmc2.zip › GBD_EOBC_final_release_v1.1.10_CLEAN/Results/Manuscript_Ready/Supplementary_Figures/Supplementary_Figure_S5_GLOBOCAN_baseline_sensitivity.pdf]
