# Supplementary material for: Breast cancer burden risk attribution and productivity loss in Chinese women aged 25 to 49
Source: iScience. 2026 Oct 14;29(11):117891. doi: 10.1016/j.isci.2026.117891 (PMC13683865; doi:10.1016/j.isci.2026.117891)
Supplement: Supplementary file 2 [file mmc2.zip › GBD_EOBC_final_release_v1.1.10_CLEAN/Results/Primary_Results/Figures_PDF/Figure 2.pdf]

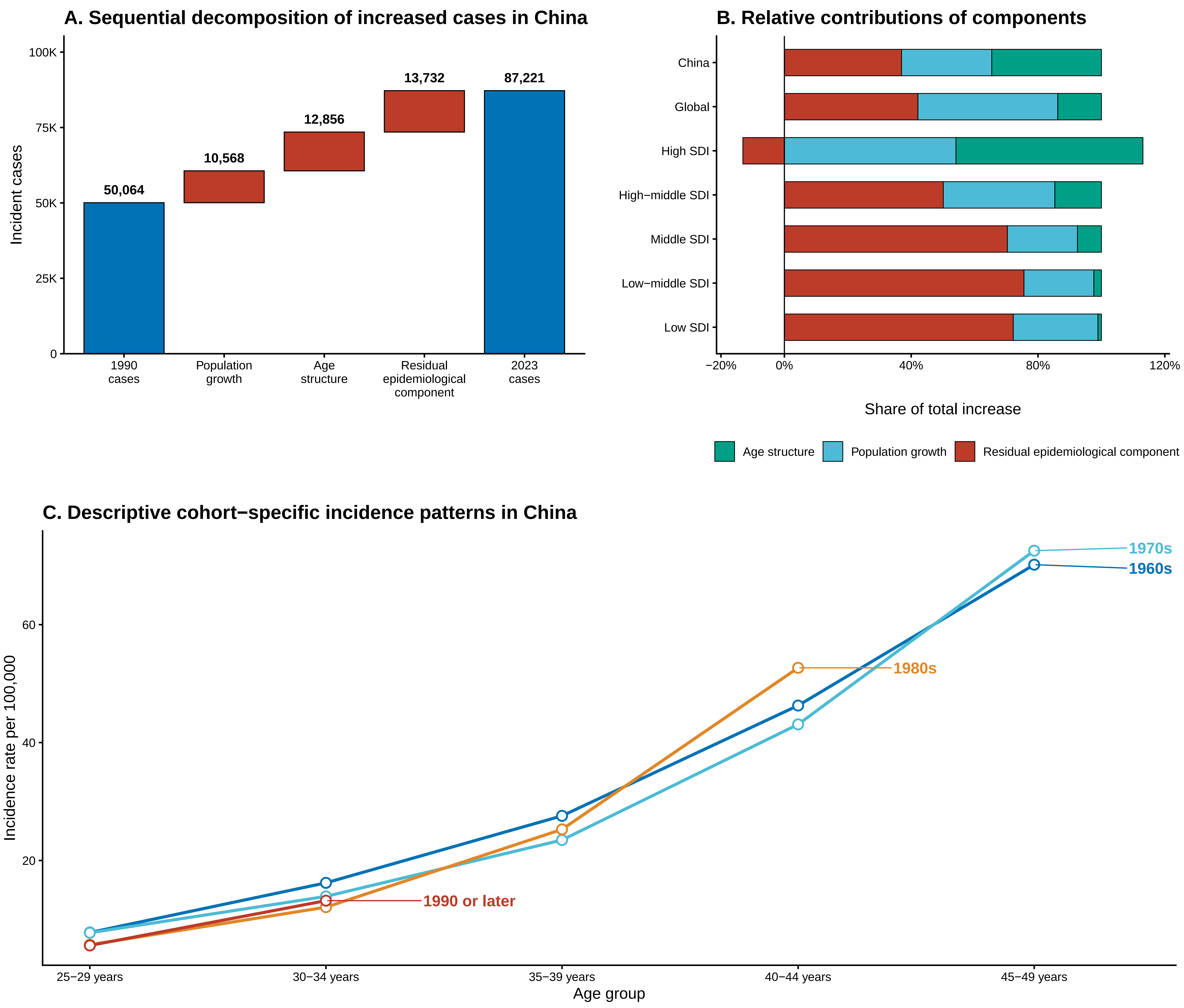
A. Sequential decomposition of increased cases in China
Incident cases
100K
75K
50K
25K
0
50,064
10,568
12,856
13,732
87,221
1990 cases
Population growth
Age structure
Residual epidemiological component
2023 cases
B. Relative contributions of components
China
Global
High SDI
High−middle SDI
Middle SDI
Low−middle SDI
Low SDI
−20%
0%
40%
80%
120%
Share of total increase
Age structure
Population growth
Residual epidemiological component
C. Descriptive cohort−specific incidence patterns in China
Incidence rate per 100,000
60
40
20
1970s
1960s
1980s
1990 or later
25−29 years
30−34 years
35−39 years
40−44 years
45−49 years
Age group
