# Supplementary material for: Breast cancer burden risk attribution and productivity loss in Chinese women aged 25 to 49
Source: iScience. 2026 Oct 14;29(11):117891. doi: 10.1016/j.isci.2026.117891 (PMC13683865; doi:10.1016/j.isci.2026.117891)
Supplement: Supplementary file 2 [file mmc2.zip › GBD_EOBC_final_release_v1.1.10_CLEAN/Results/Primary_Results/Figures_PDF/Figure 5.pdf]

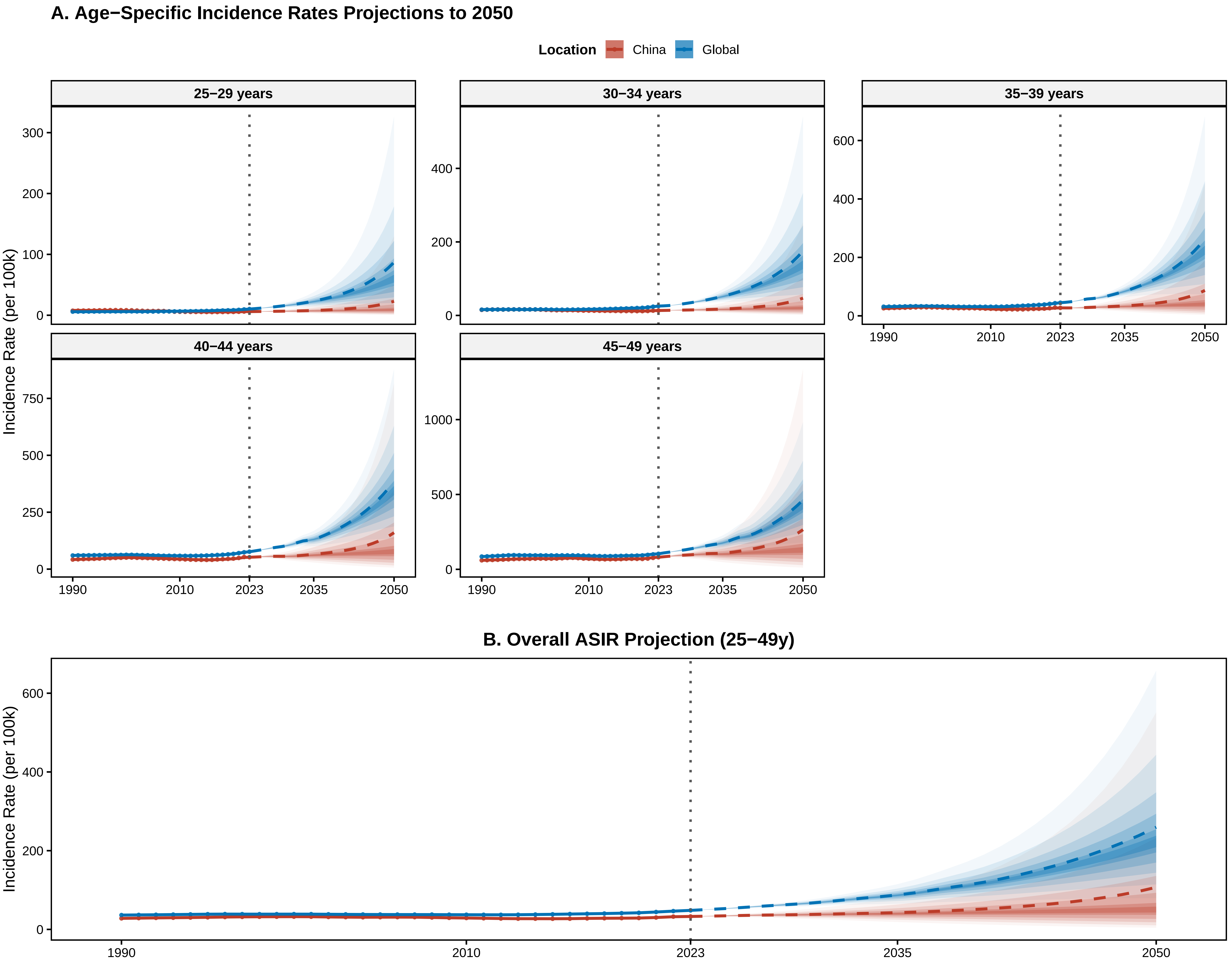
A. Age−Specific Incidence Rates Projections to 2050
Location
China
Global
25−29 years
30−34 years
35−39 years
40−44 years
45−49 years
Incidence Rate (per 100k)
1990
2010
2023
2035
2050
B. Overall ASIR Projection (25−49y)
Incidence Rate (per 100k)
