# Supplementary material for: Breast cancer burden risk attribution and productivity loss in Chinese women aged 25 to 49
Source: iScience. 2026 Oct 14;29(11):117891. doi: 10.1016/j.isci.2026.117891 (PMC13683865; doi:10.1016/j.isci.2026.117891)
Supplement: Supplementary file 2 [file mmc2.zip › GBD_EOBC_final_release_v1.1.10_CLEAN/Results/Primary_Results/Figures_PDF/Figure 6.pdf]

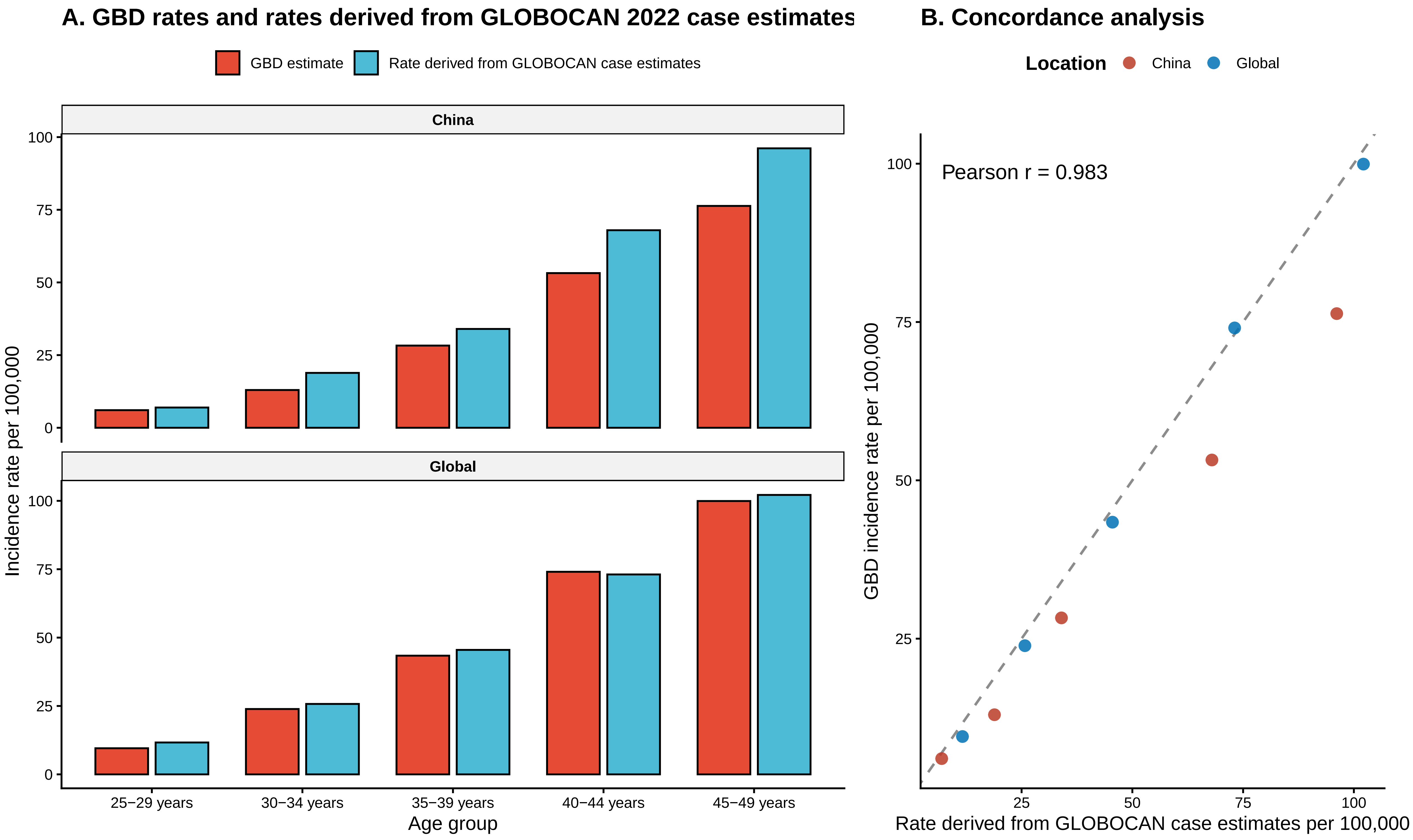
A. GBD rates and rates derived from GLOBOCAN 2022 case estimates
GBD estimate
Rate derived from GLOBOCAN case estimates
China
Global
Incidence rate per 100,000
0
25
50
75
100
25−29 years
30−34 years
35−39 years
40−44 years
45−49 years
Age group
B. Concordance analysis
Location
China
Global
Pearson r = 0.983
GBD incidence rate per 100,000
Rate derived from GLOBOCAN case estimates per 100,000
